# Supplementary material for: Informed Consent for Academic Surgeons: A Curriculum-Based Update
Source: MedEdPORTAL. 2020 Oct 1;16:10985. doi: 10.15766/mep_2374-8265.10985 (PMC7528671; doi:10.15766/mep_2374-8265.10985)
Supplement: Supplementary file 1 — Informed Consent Update Slide Deck.pptxFacilitator Guide.docxInformed Consent Update Evaluation.docxKnowledge Posttest Questions.docx [file mep_2374-8265.10985-s001.zip › A. Informed Consent Update Slide Deck.pptx]

## Slide 1
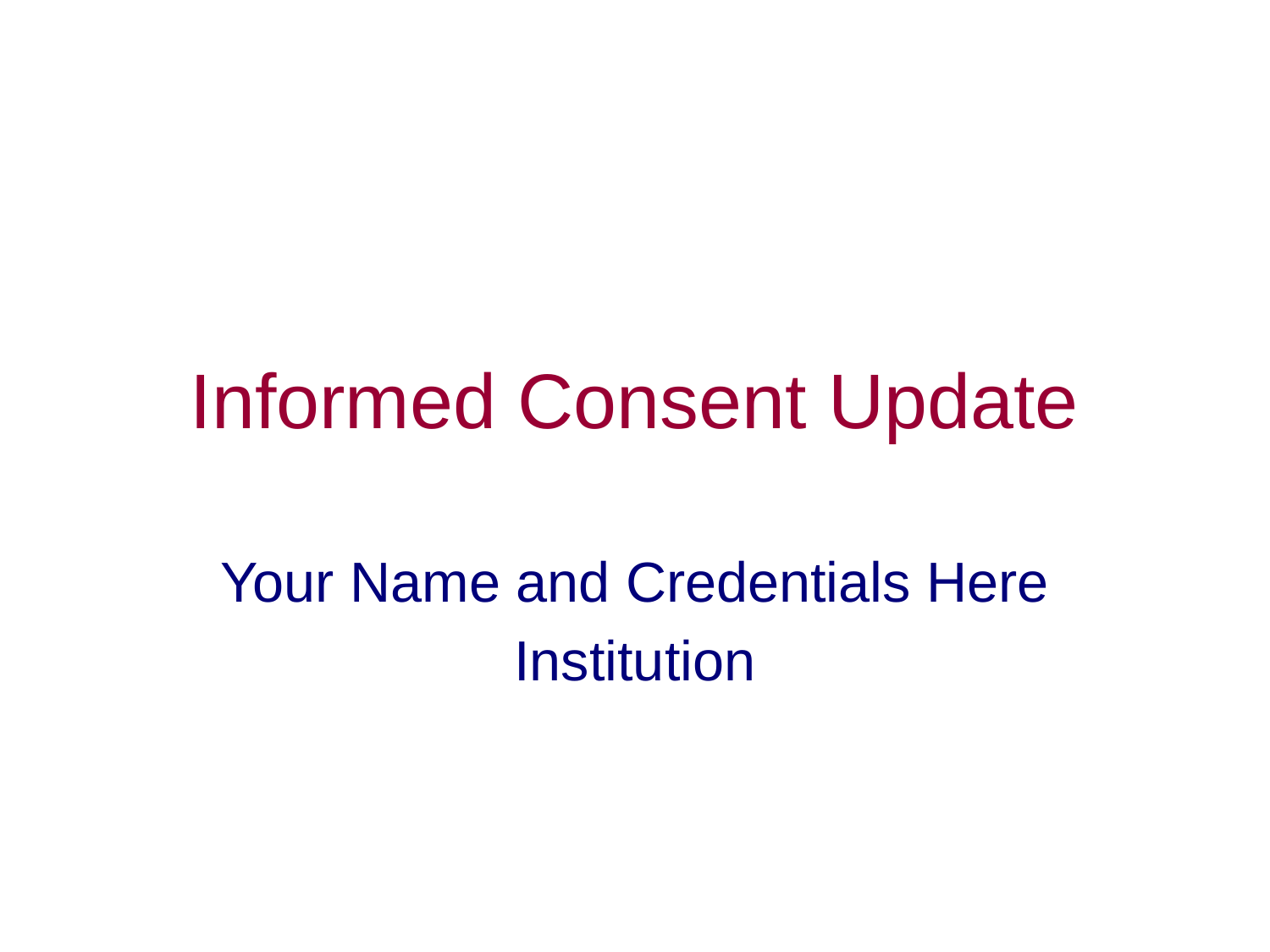

# Informed Consent Update
Your Name and Credentials Here
Institution

## Slide 2
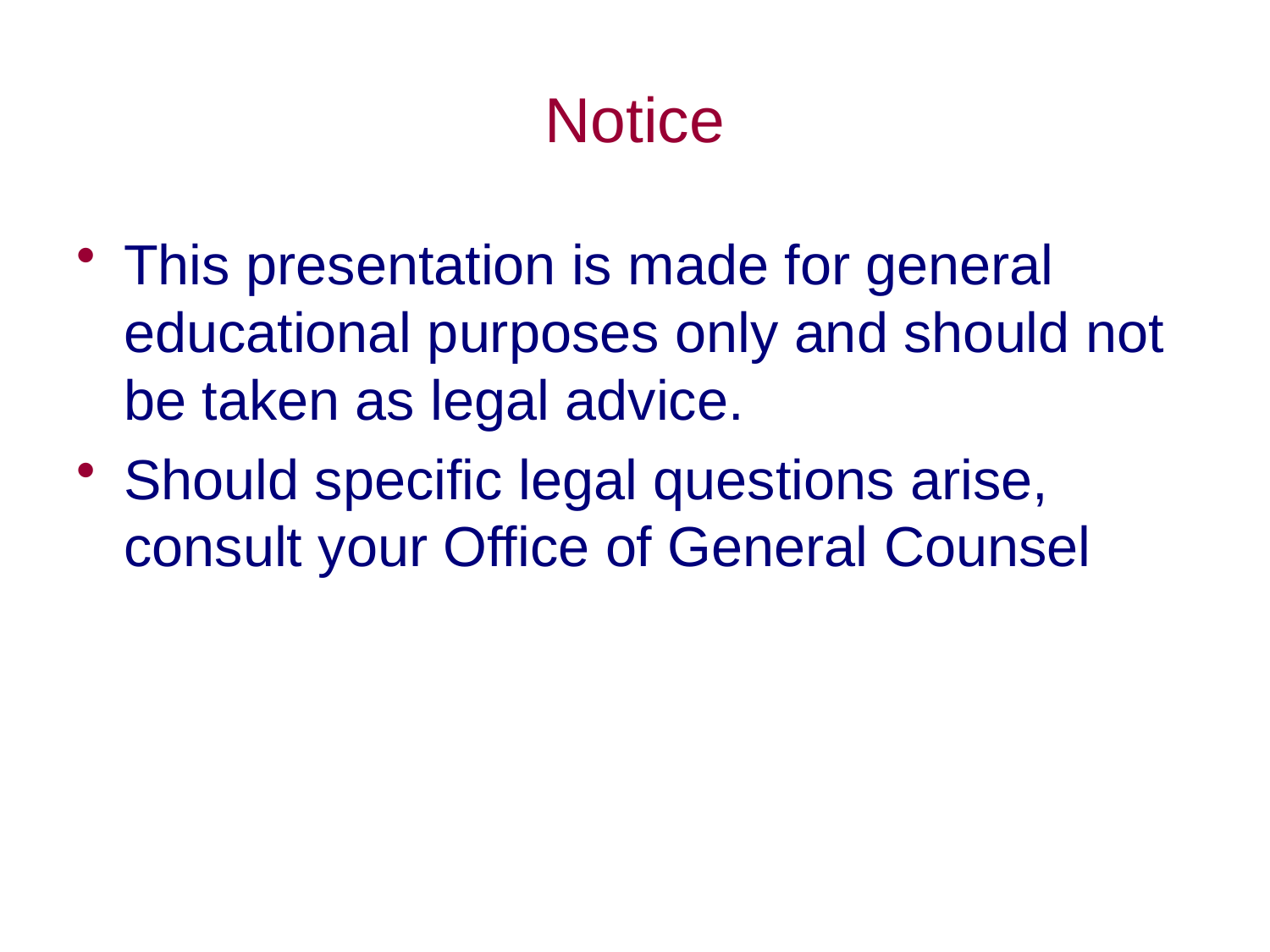

# Notice
This presentation is made for general educational purposes only and should not be taken as legal advice.
Should specific legal questions arise, consult your Office of General Counsel

## Slide 3
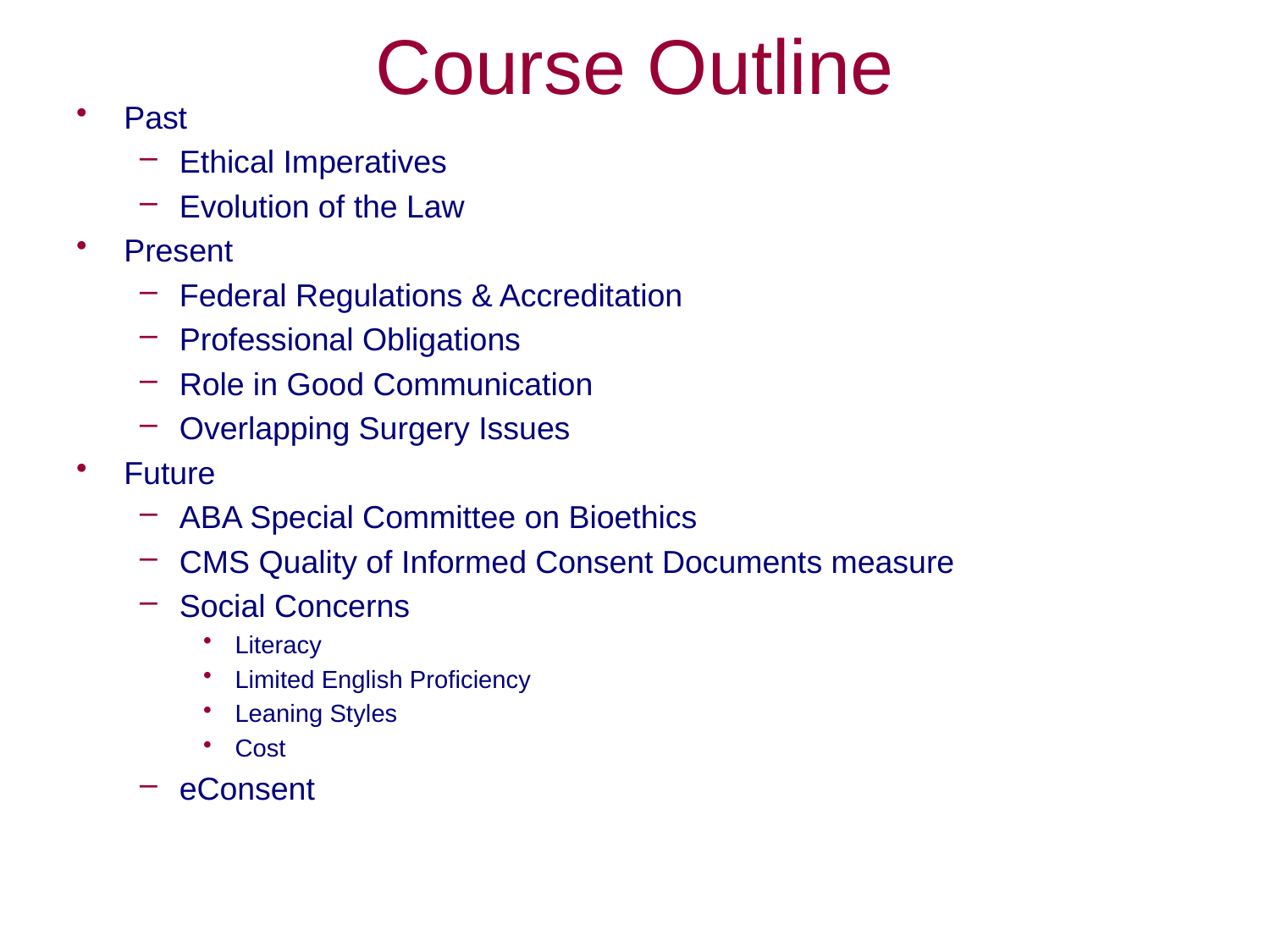

# Course Outline
Past
Ethical Imperatives
Evolution of the Law
Present
Federal Regulations & Accreditation
Professional Obligations
Role in Good Communication
Overlapping Surgery Issues
Future
ABA Special Committee on Bioethics
CMS Quality of Informed Consent Documents measure
Social Concerns
Literacy
Limited English Proficiency
Leaning Styles
Cost
eConsent

## Slide 4
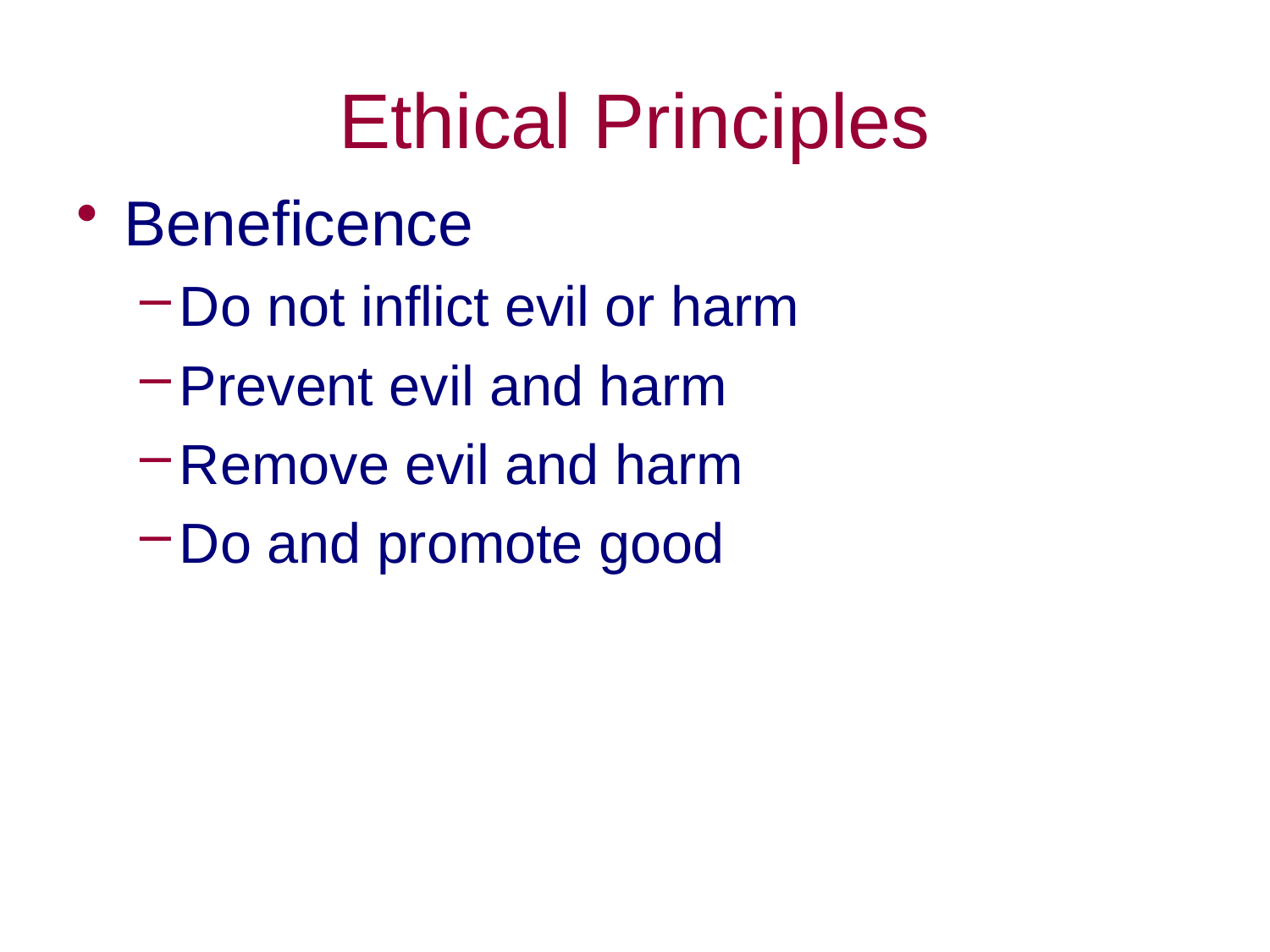

# Ethical Principles
Beneficence
Do not inflict evil or harm
Prevent evil and harm
Remove evil and harm
Do and promote good

## Slide 5
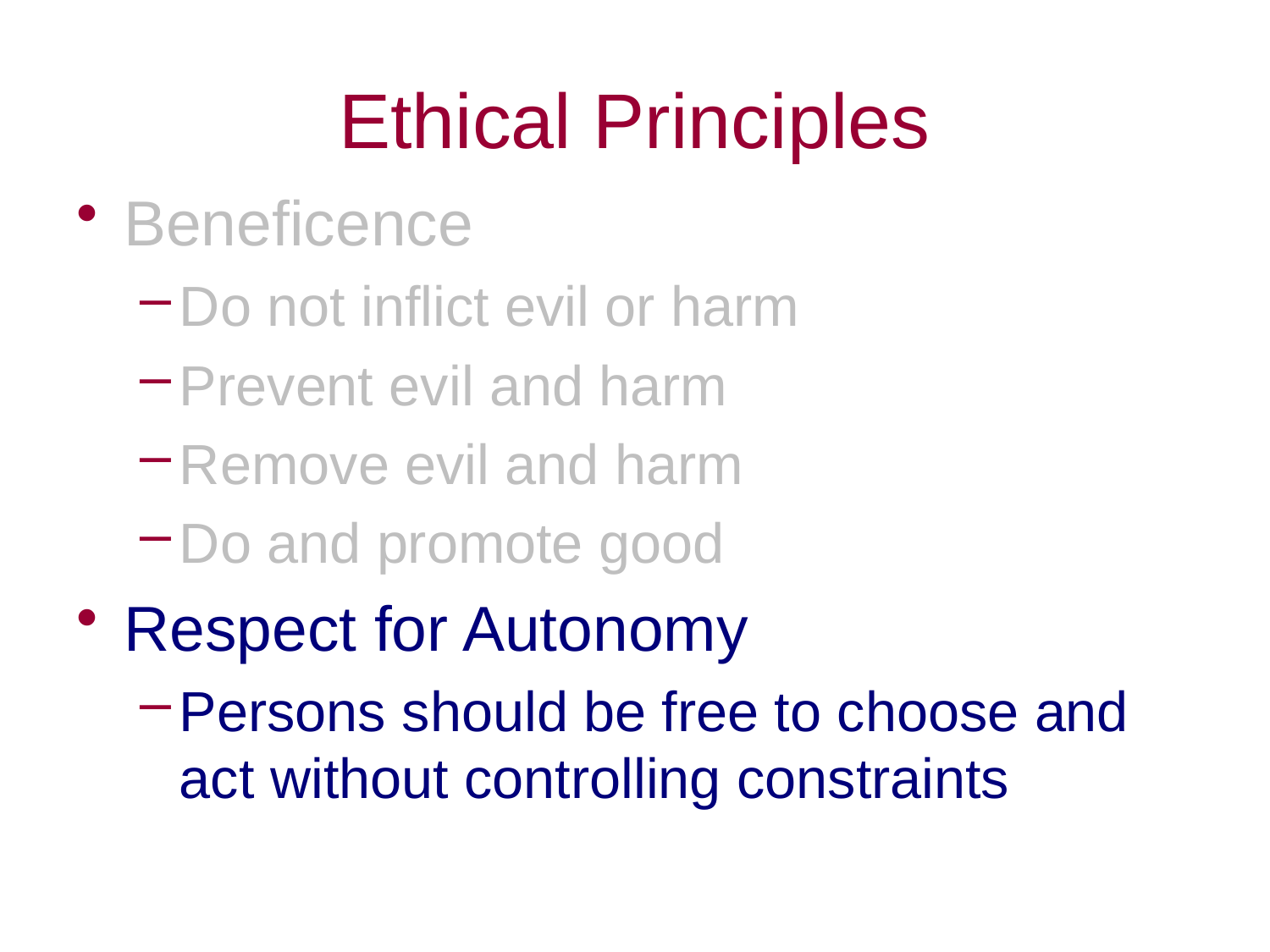

# Ethical Principles
Beneficence
Do not inflict evil or harm
Prevent evil and harm
Remove evil and harm
Do and promote good
Respect for Autonomy
Persons should be free to choose and act without controlling constraints

## Slide 6
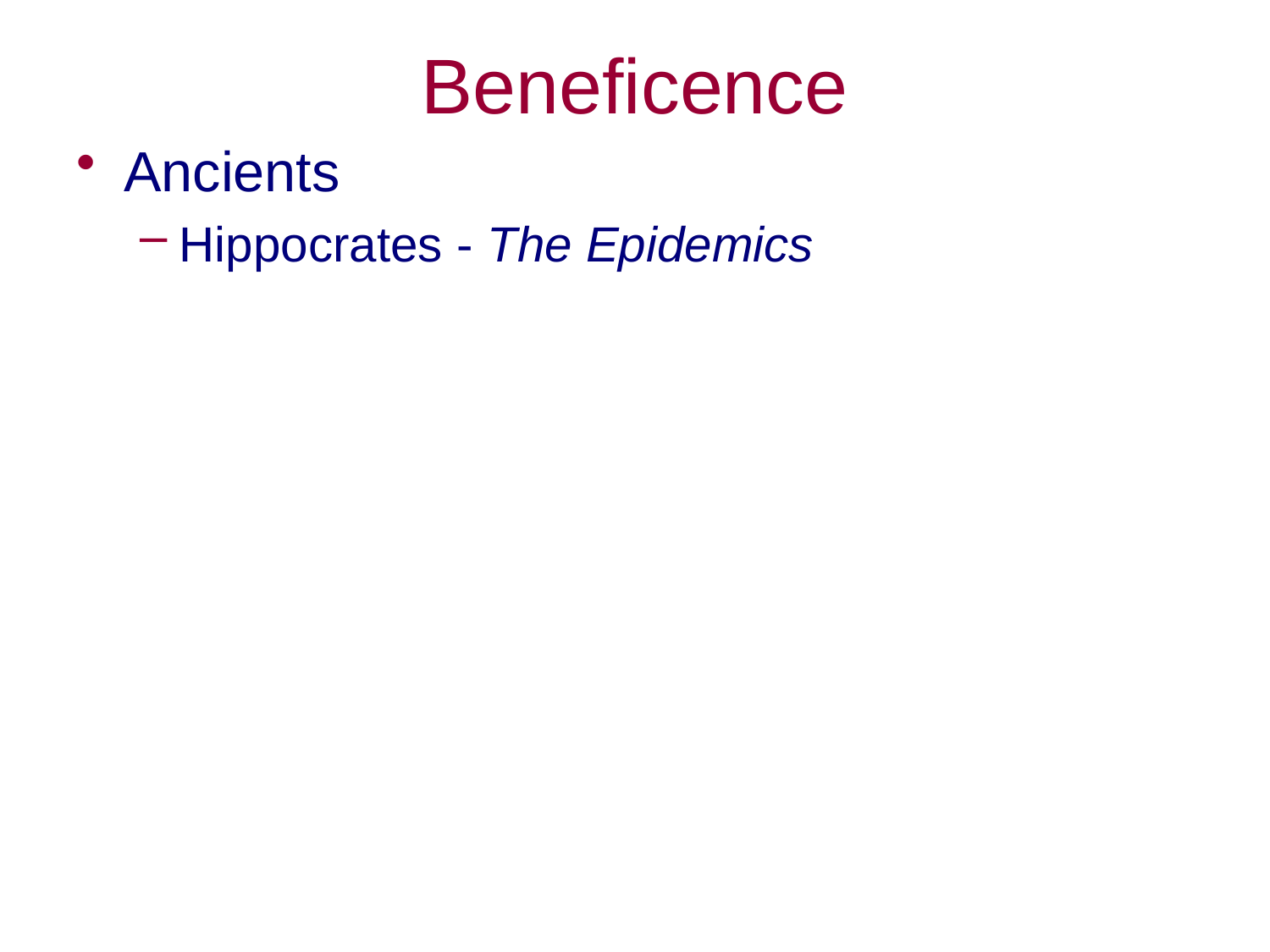

# Beneficence
Ancients
Hippocrates - The Epidemics

## Slide 7
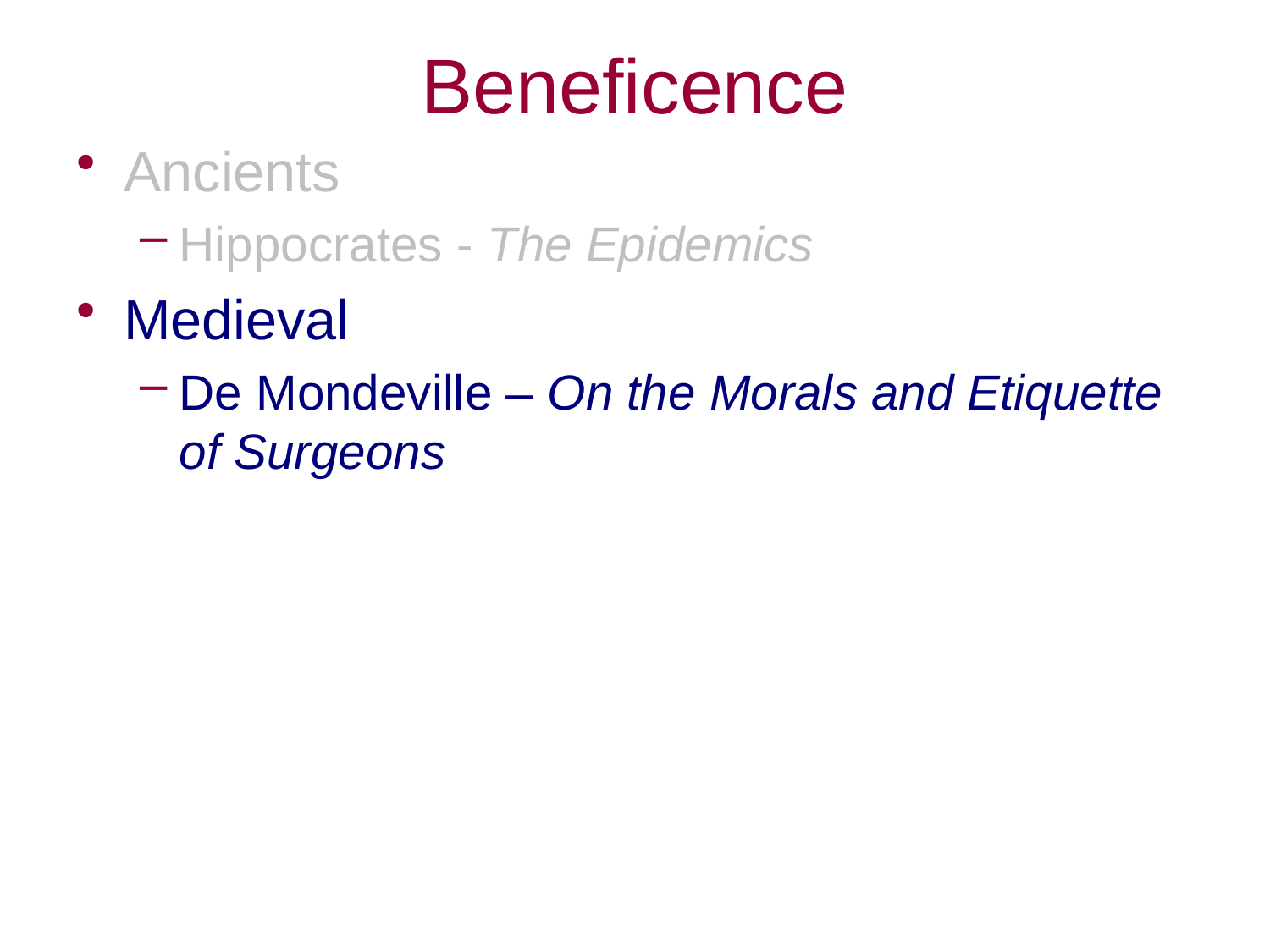

# Beneficence
Ancients
Hippocrates - The Epidemics
Medieval
De Mondeville – On the Morals and Etiquette of Surgeons

## Slide 8
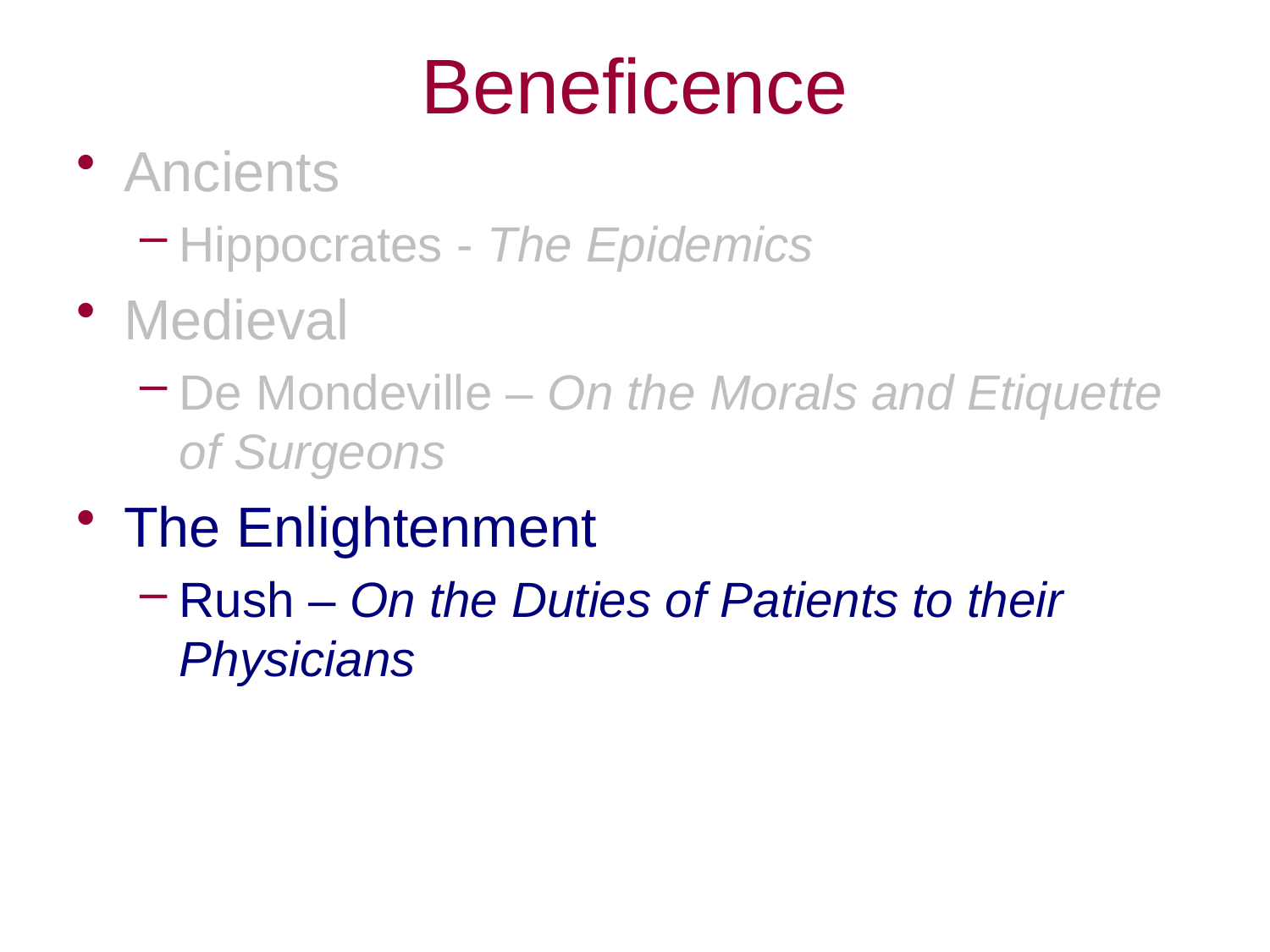

# Beneficence
Ancients
Hippocrates - The Epidemics
Medieval
De Mondeville – On the Morals and Etiquette of Surgeons
The Enlightenment
Rush – On the Duties of Patients to their Physicians

## Slide 9
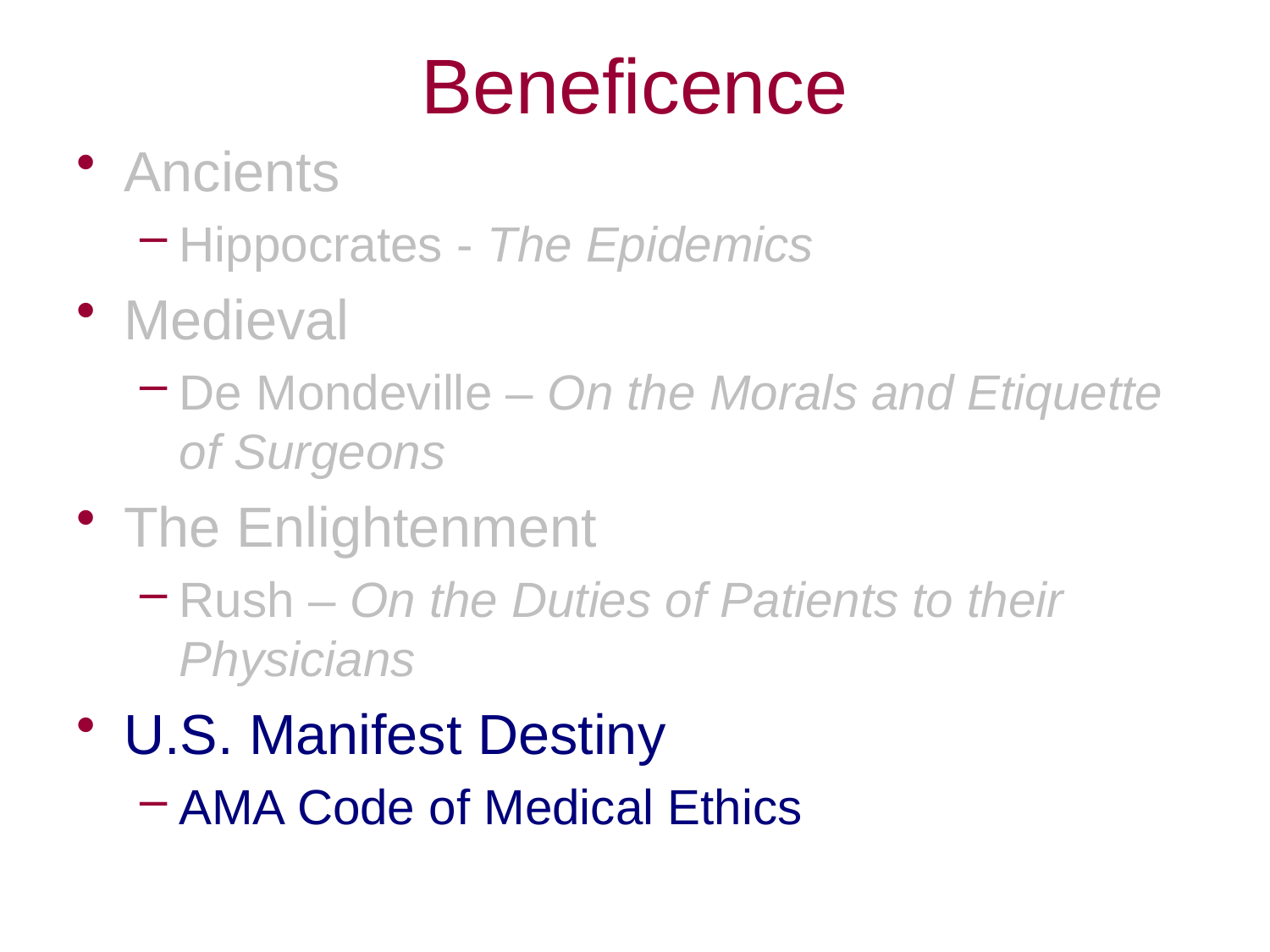

# Beneficence
Ancients
Hippocrates - The Epidemics
Medieval
De Mondeville – On the Morals and Etiquette of Surgeons
The Enlightenment
Rush – On the Duties of Patients to their Physicians
U.S. Manifest Destiny
AMA Code of Medical Ethics

## Slide 10
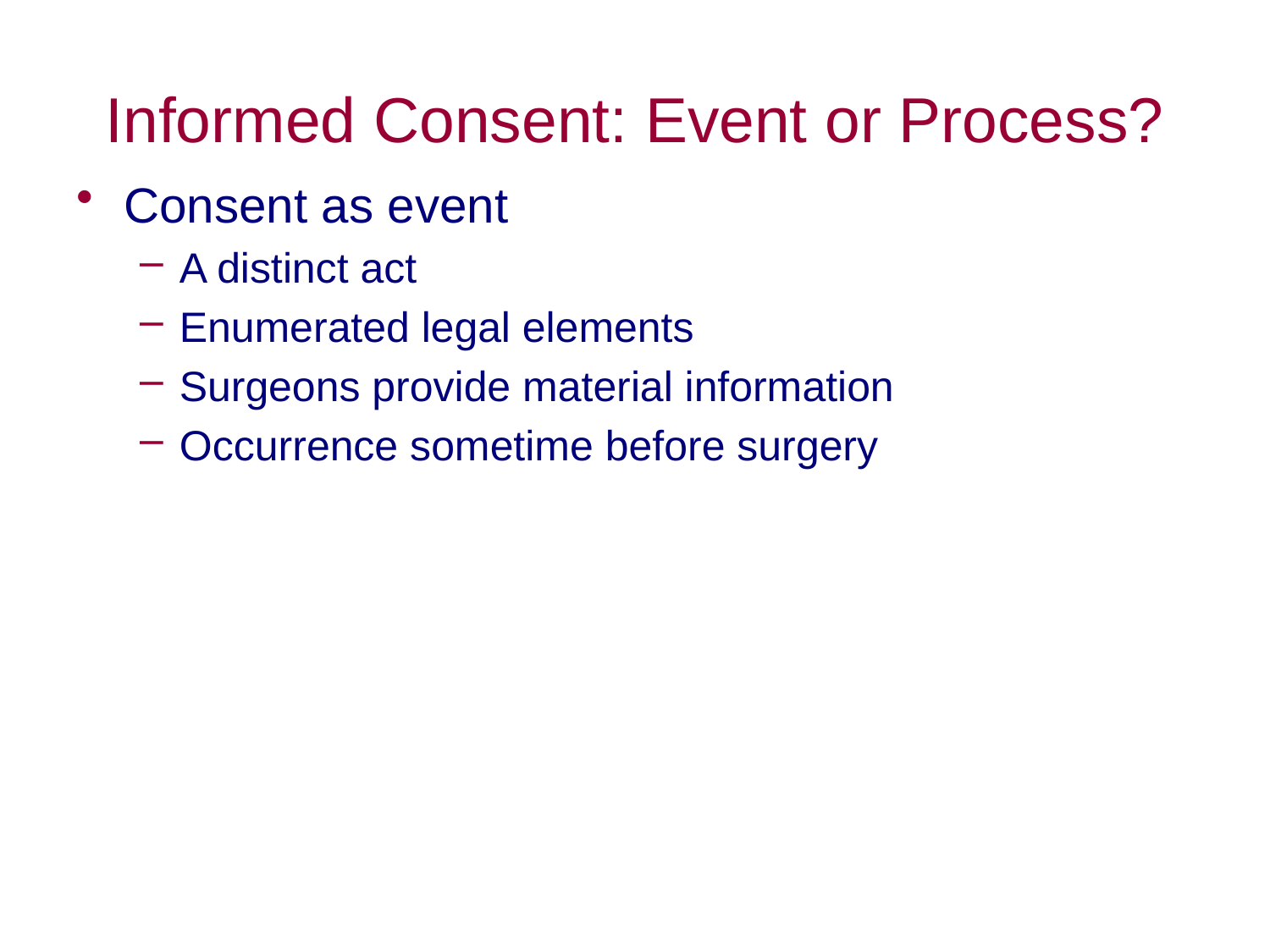

# Informed Consent: Event or Process?
Consent as event
A distinct act
Enumerated legal elements
Surgeons provide material information
Occurrence sometime before surgery

## Slide 11
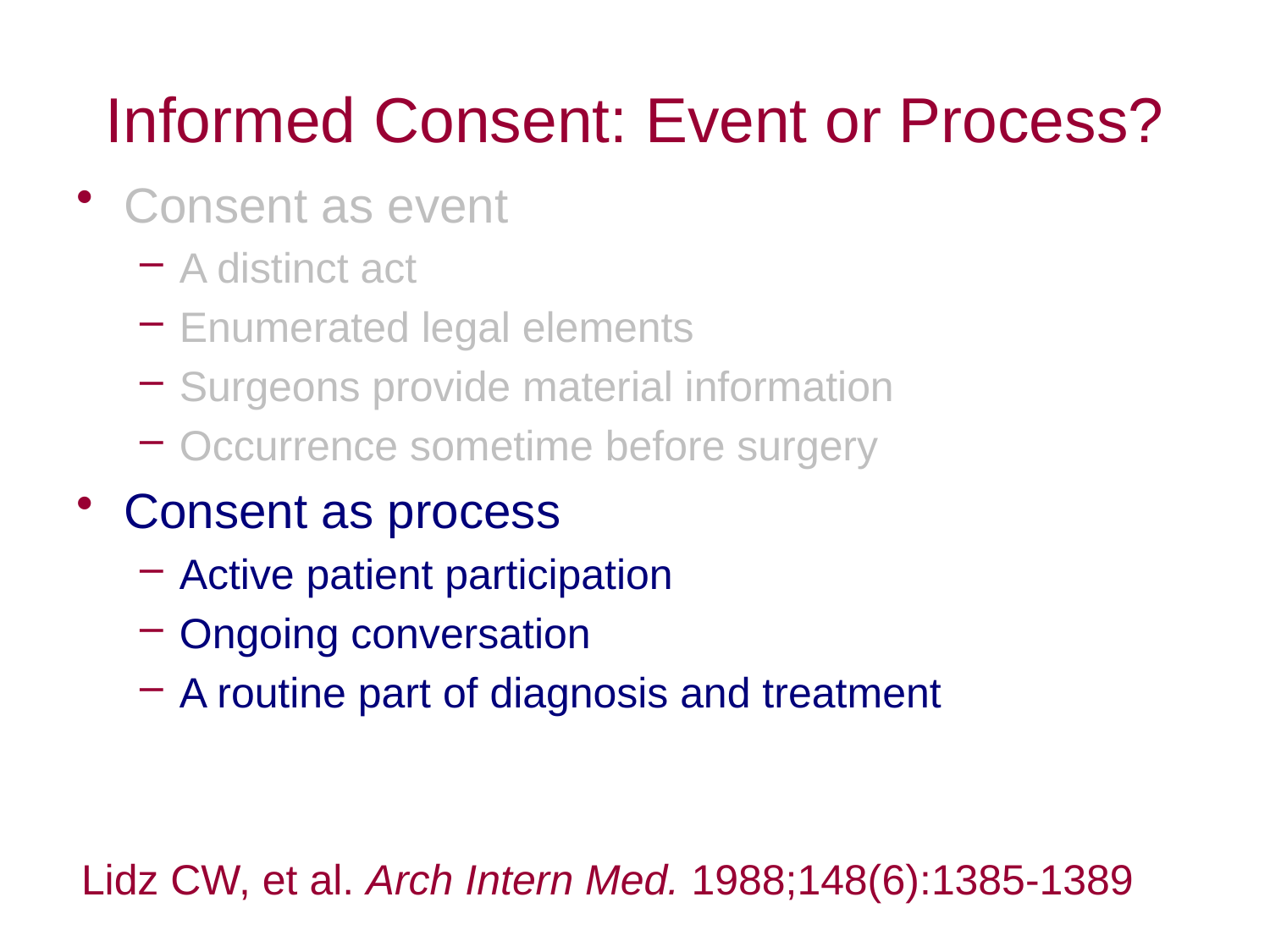

# Informed Consent: Event or Process?
Consent as event
A distinct act
Enumerated legal elements
Surgeons provide material information
Occurrence sometime before surgery
Consent as process
Active patient participation
Ongoing conversation
A routine part of diagnosis and treatment
Lidz CW, et al. Arch Intern Med. 1988;148(6):1385-1389

## Slide 12
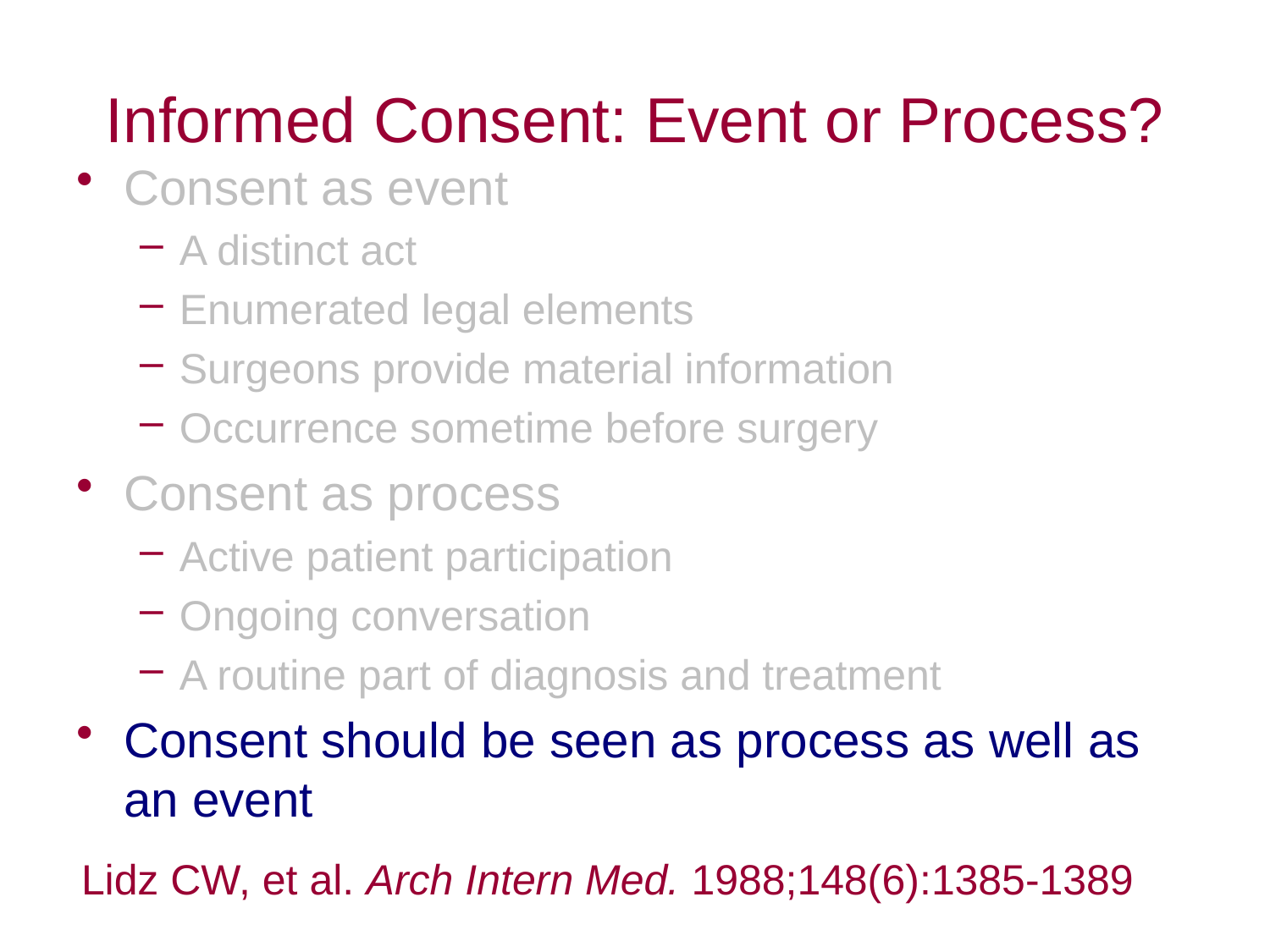

# Informed Consent: Event or Process?
Consent as event
A distinct act
Enumerated legal elements
Surgeons provide material information
Occurrence sometime before surgery
Consent as process
Active patient participation
Ongoing conversation
A routine part of diagnosis and treatment
Consent should be seen as process as well as an event
Lidz CW, et al. Arch Intern Med. 1988;148(6):1385-1389

## Slide 13
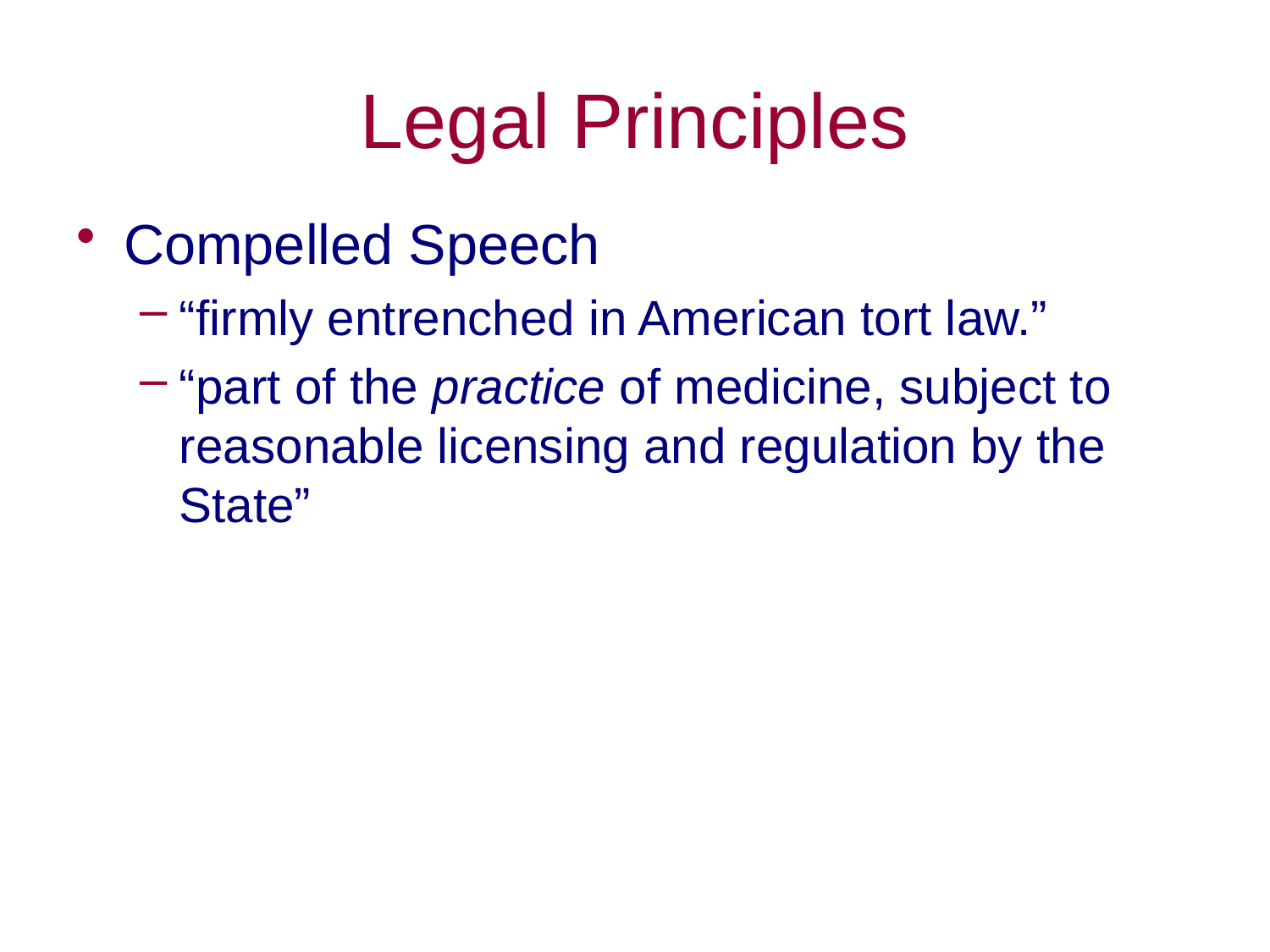

# Legal Principles
Compelled Speech
“firmly entrenched in American tort law.”
“part of the practice of medicine, subject to reasonable licensing and regulation by the State”

## Slide 14
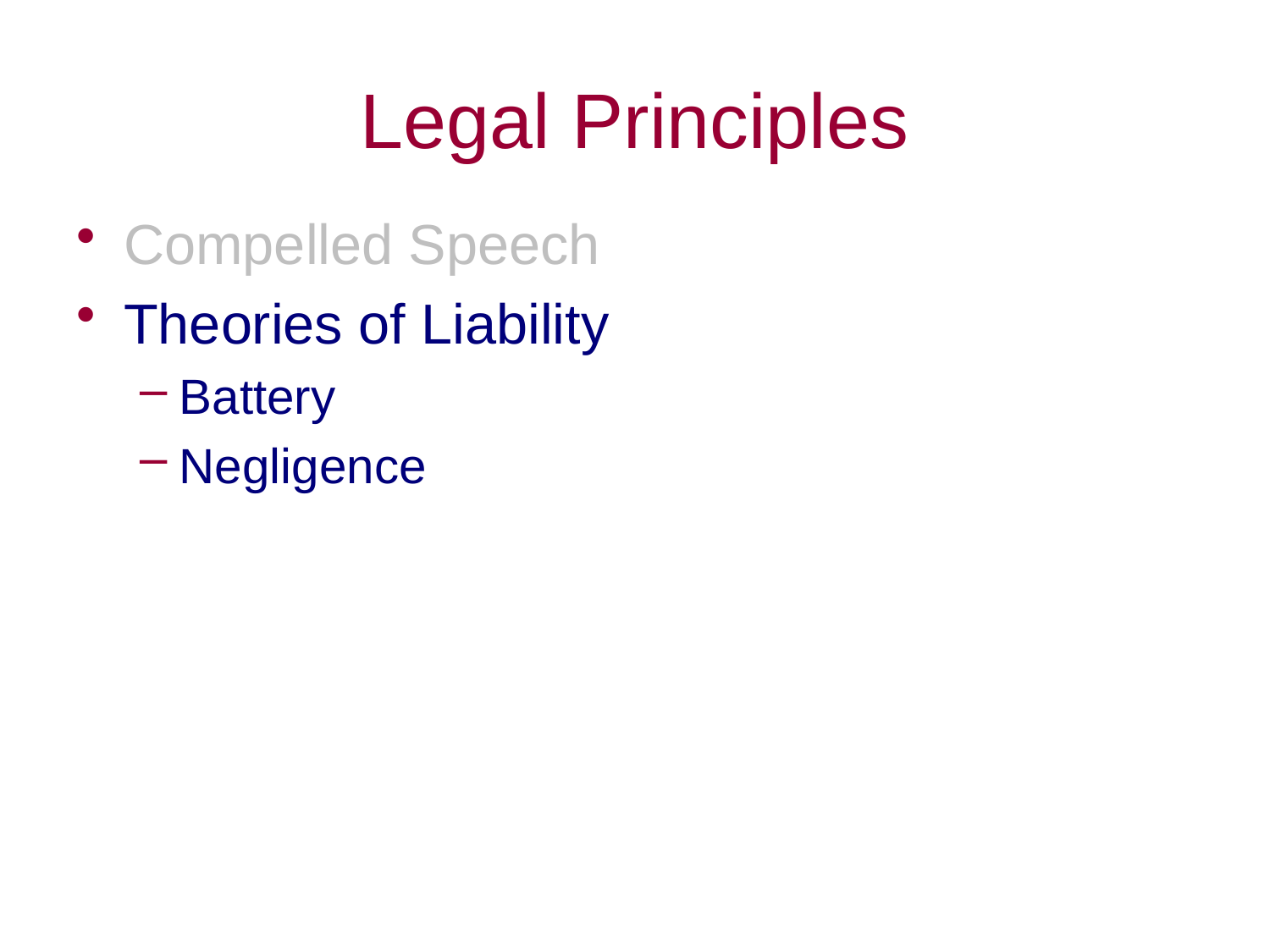

# Legal Principles
Compelled Speech
Theories of Liability
Battery
Negligence

## Slide 15
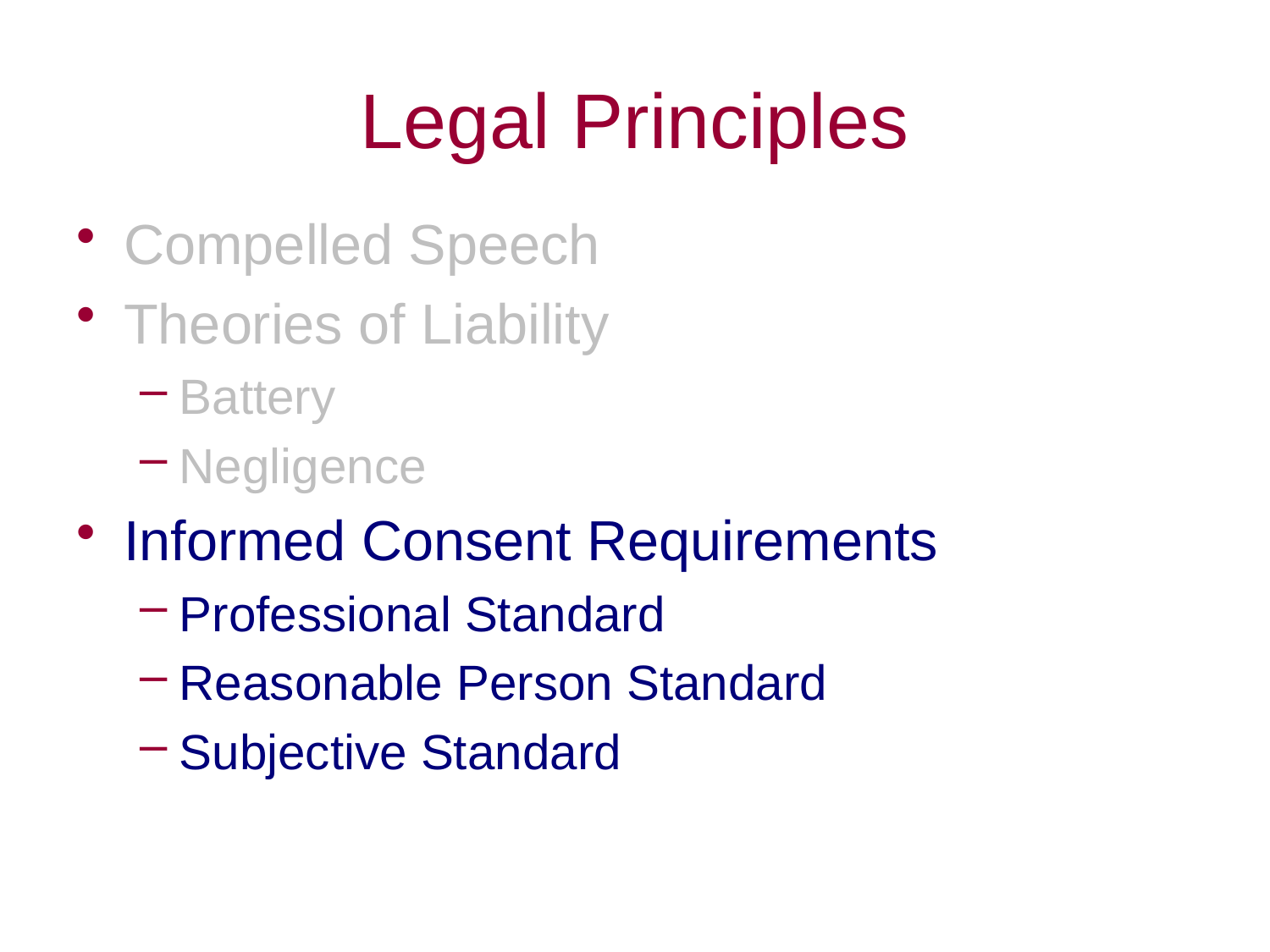

# Legal Principles
Compelled Speech
Theories of Liability
Battery
Negligence
Informed Consent Requirements
Professional Standard
Reasonable Person Standard
Subjective Standard

## Slide 16
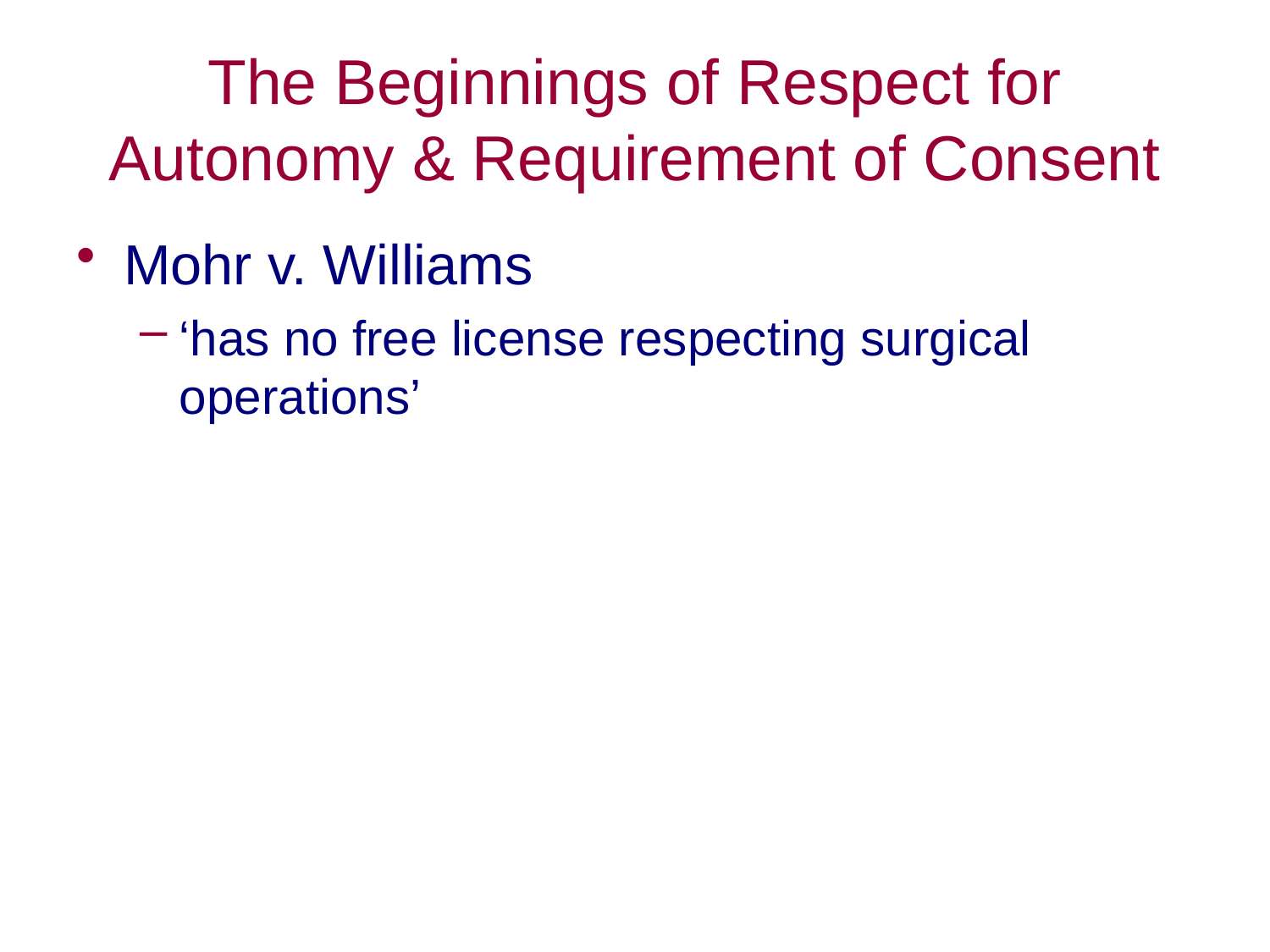

# The Beginnings of Respect for Autonomy & Requirement of Consent
Mohr v. Williams
‘has no free license respecting surgical operations’

## Slide 17
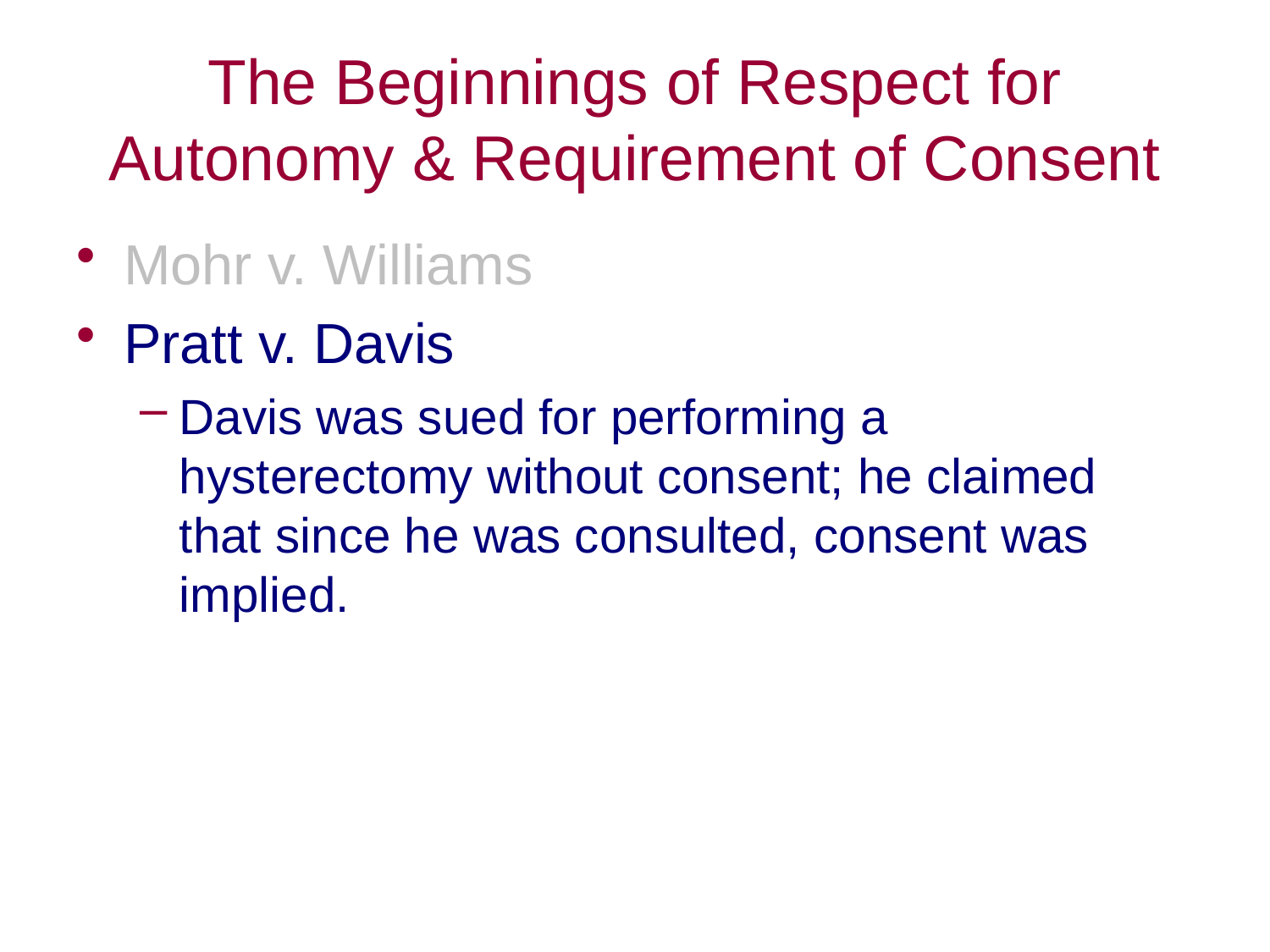

# The Beginnings of Respect for Autonomy & Requirement of Consent
Mohr v. Williams
Pratt v. Davis
Davis was sued for performing a hysterectomy without consent; he claimed that since he was consulted, consent was implied.

## Slide 18
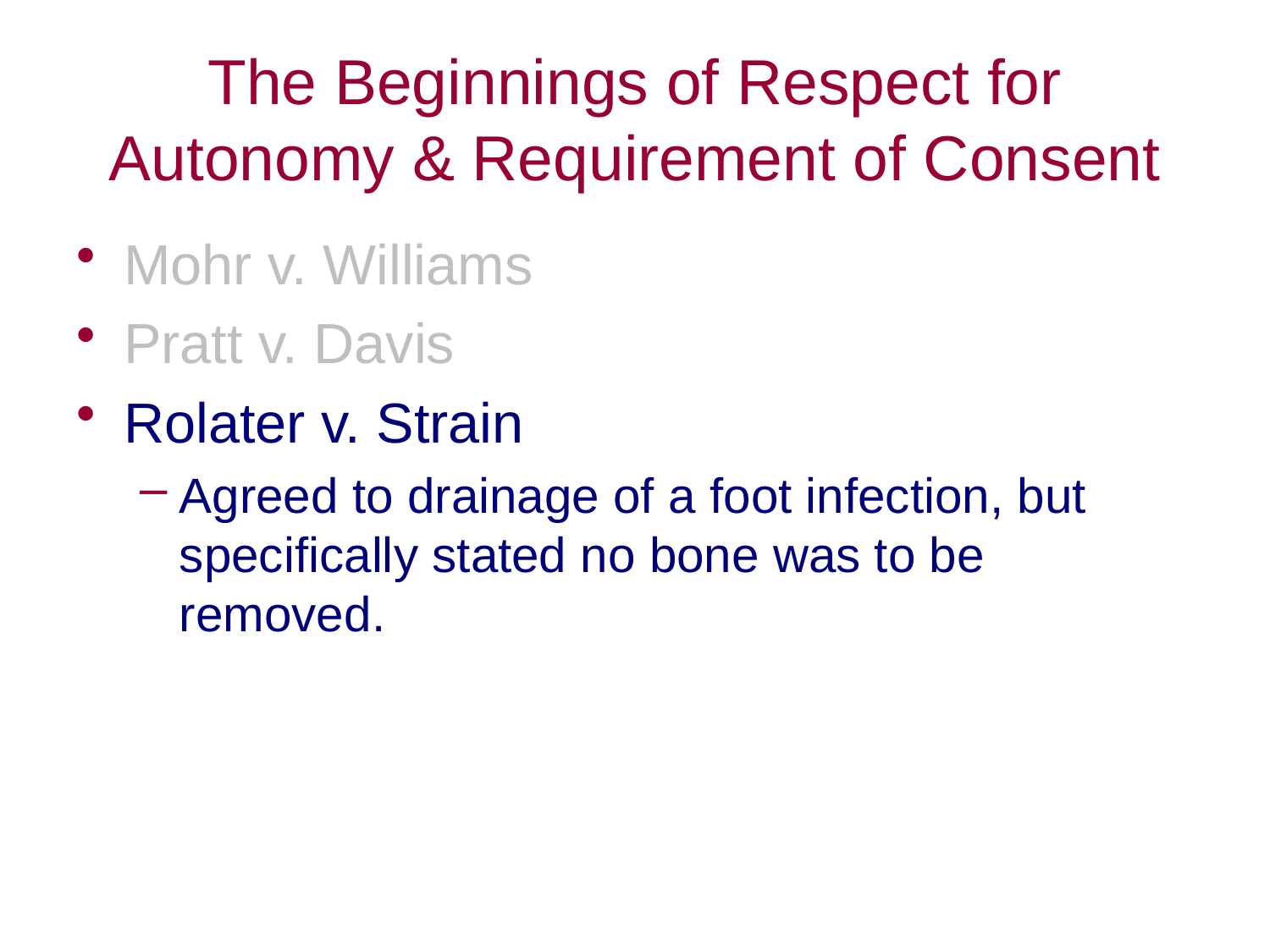

# The Beginnings of Respect for Autonomy & Requirement of Consent
Mohr v. Williams
Pratt v. Davis
Rolater v. Strain
Agreed to drainage of a foot infection, but specifically stated no bone was to be removed.

## Slide 19
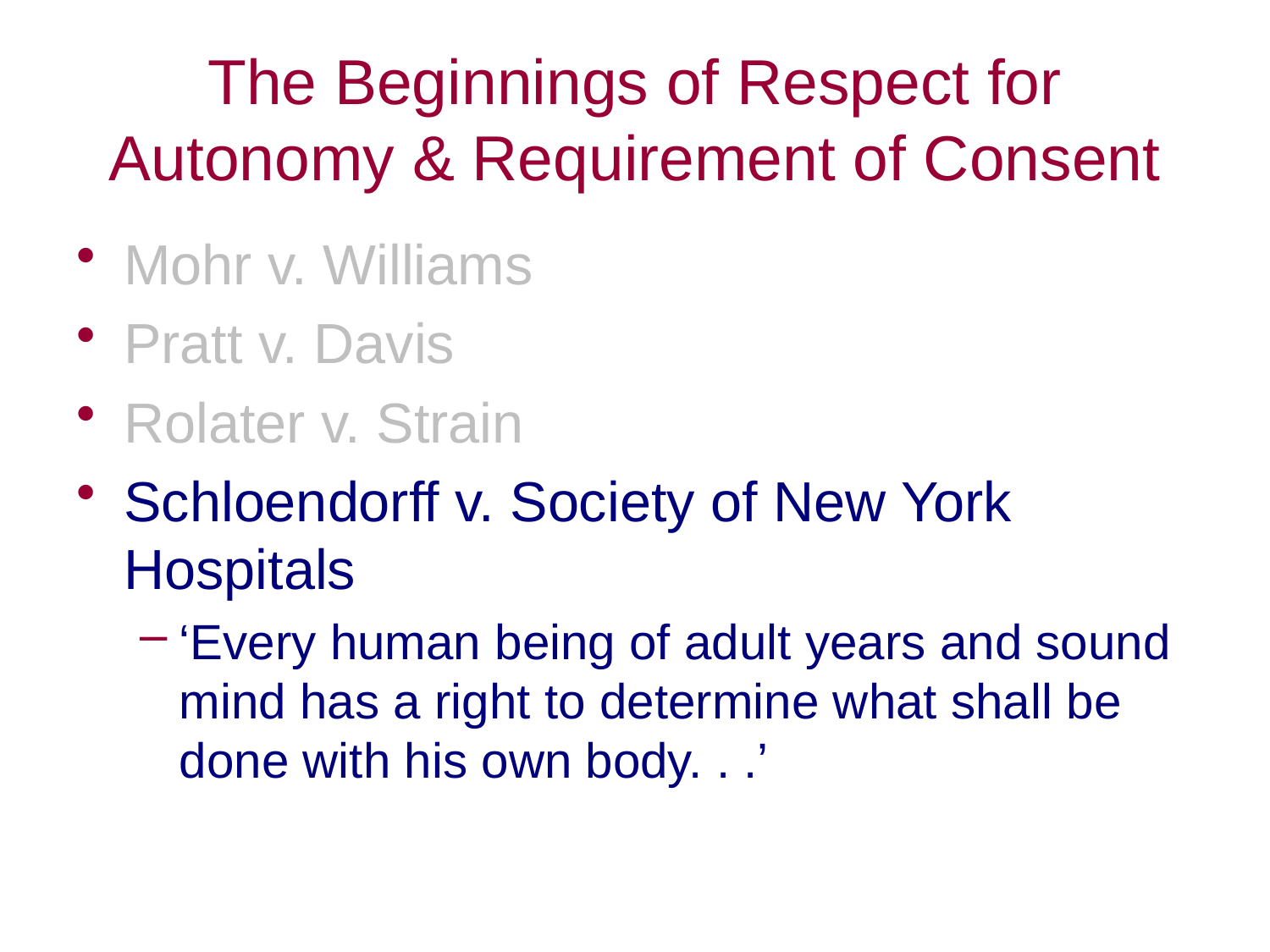

# The Beginnings of Respect for Autonomy & Requirement of Consent
Mohr v. Williams
Pratt v. Davis
Rolater v. Strain
Schloendorff v. Society of New York Hospitals
‘Every human being of adult years and sound mind has a right to determine what shall be done with his own body. . .’

## Slide 20
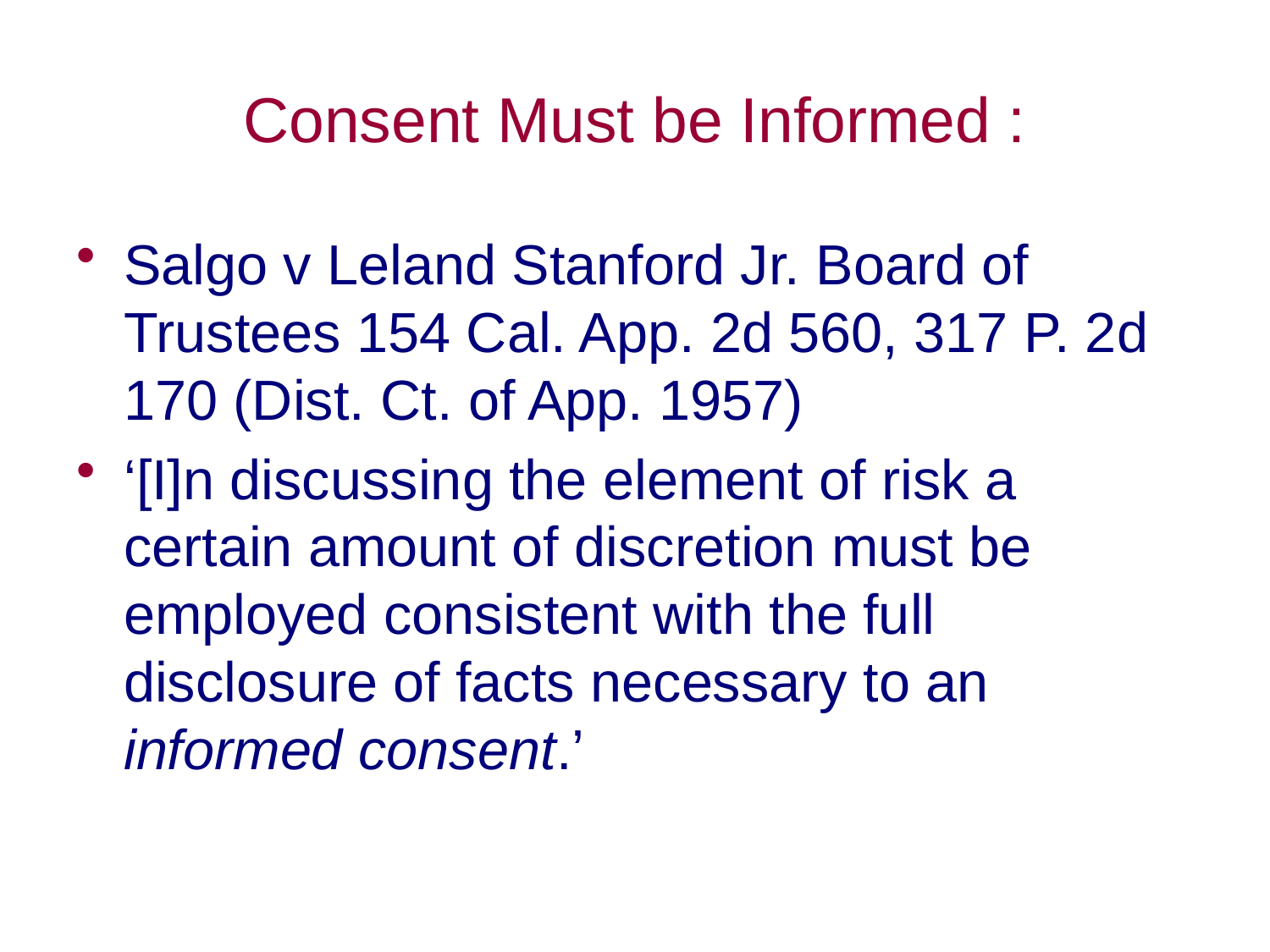

# Consent Must be Informed :
Salgo v Leland Stanford Jr. Board of Trustees 154 Cal. App. 2d 560, 317 P. 2d 170 (Dist. Ct. of App. 1957)
‘[I]n discussing the element of risk a certain amount of discretion must be employed consistent with the full disclosure of facts necessary to an informed consent.’

## Slide 21
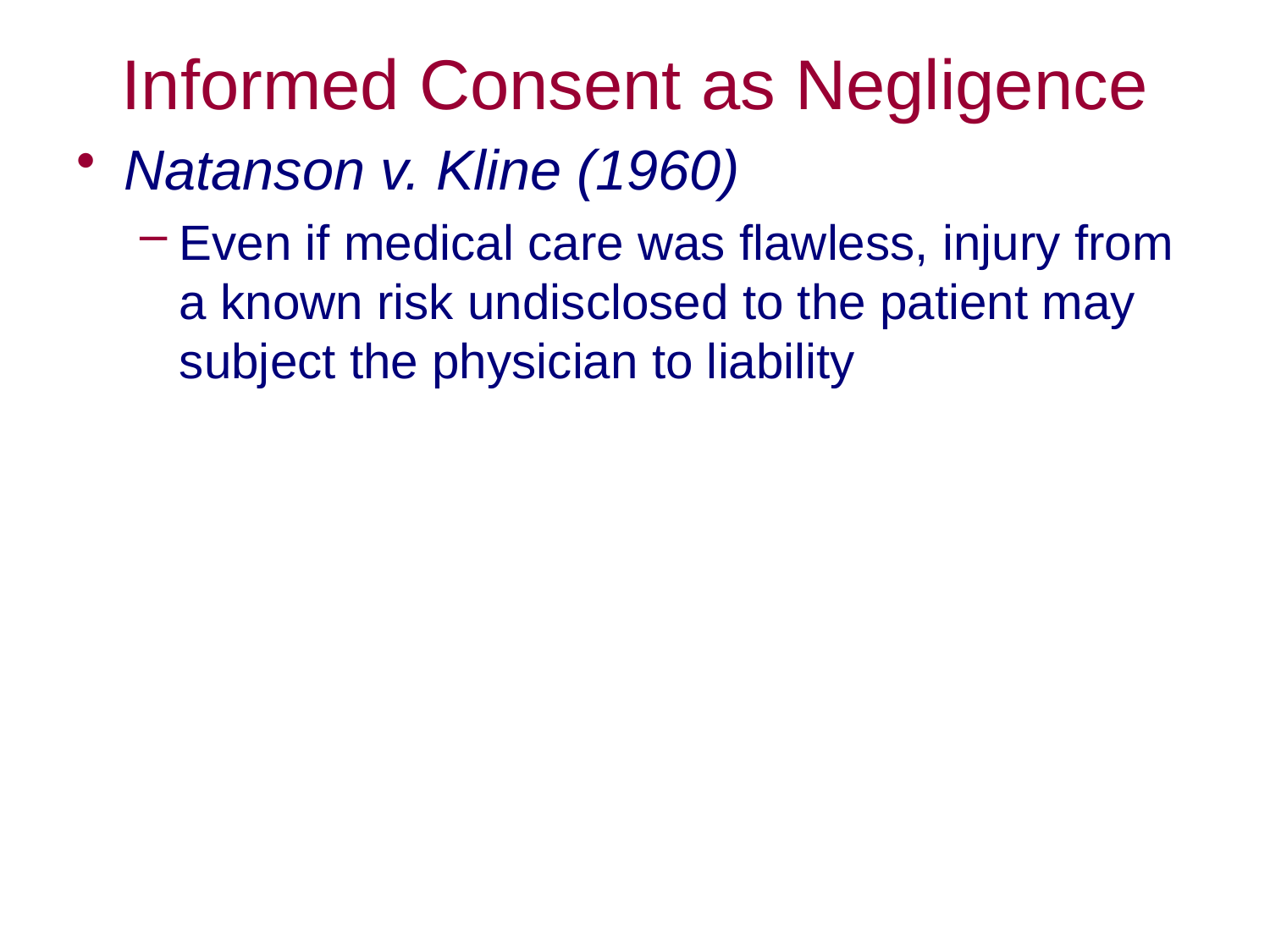

# Informed Consent as Negligence
Natanson v. Kline (1960)
Even if medical care was flawless, injury from a known risk undisclosed to the patient may subject the physician to liability

## Slide 22
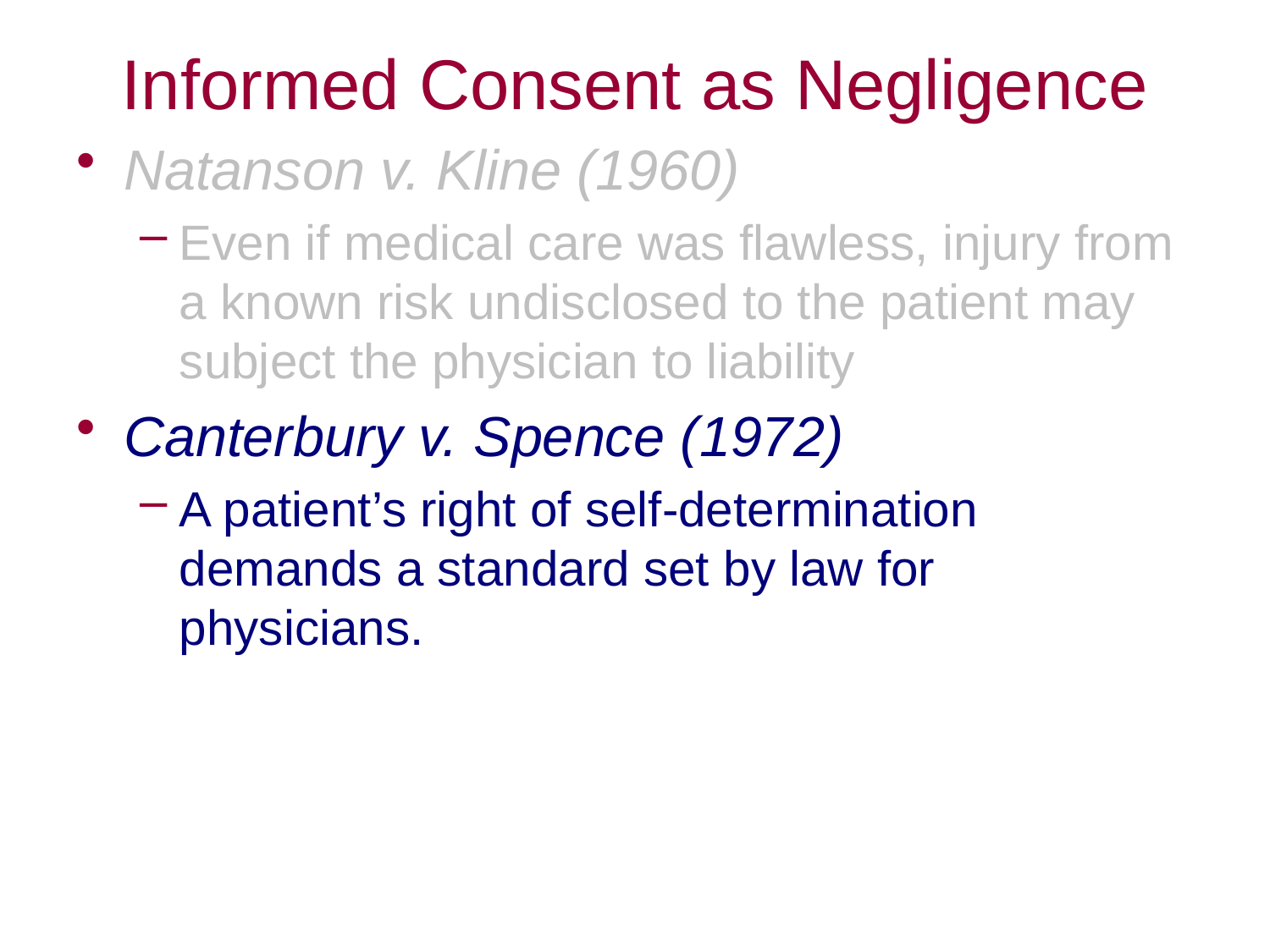

# Informed Consent as Negligence
Natanson v. Kline (1960)
Even if medical care was flawless, injury from a known risk undisclosed to the patient may subject the physician to liability
Canterbury v. Spence (1972)
A patient’s right of self-determination demands a standard set by law for physicians.

## Slide 23
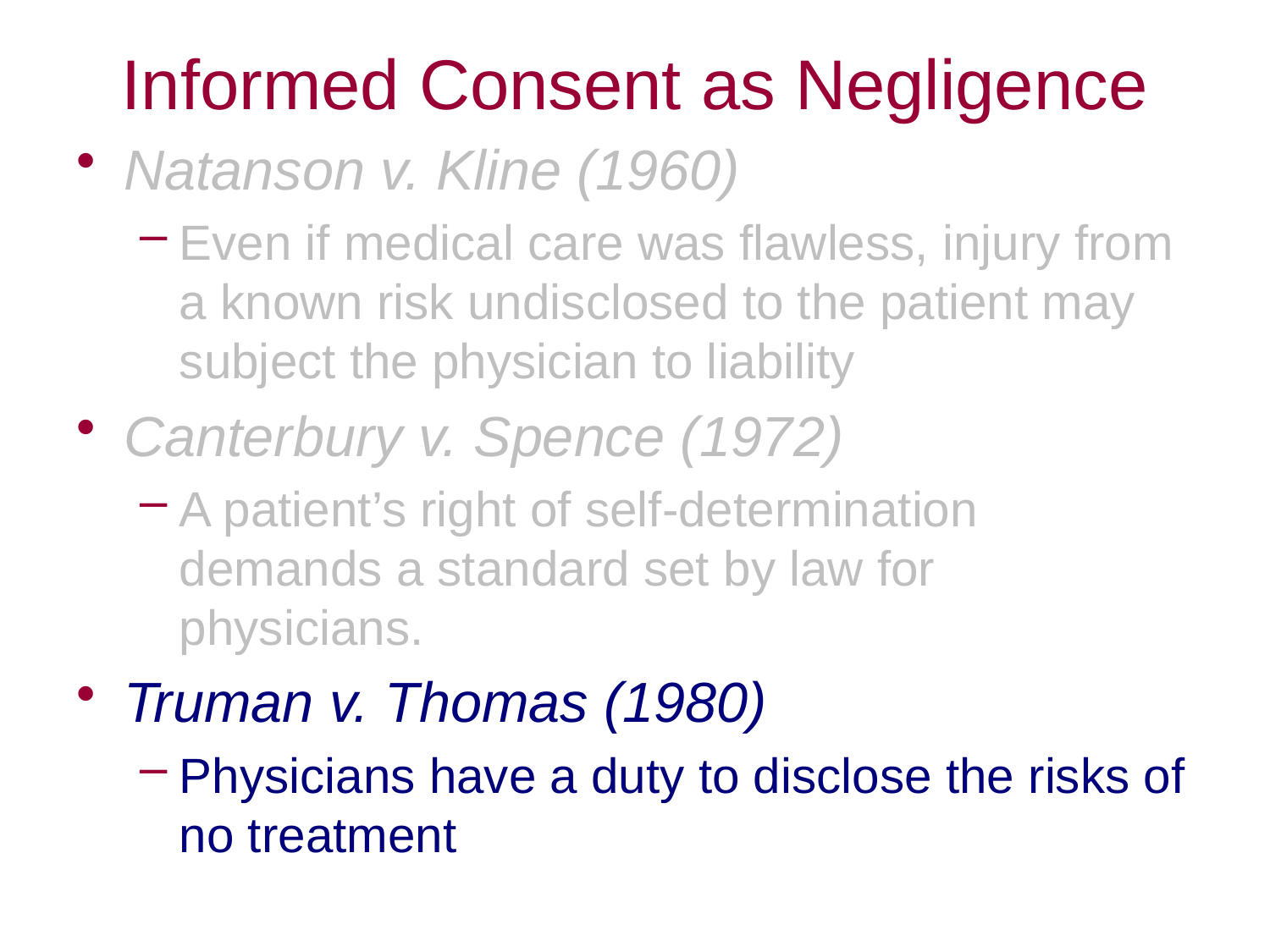

# Informed Consent as Negligence
Natanson v. Kline (1960)
Even if medical care was flawless, injury from a known risk undisclosed to the patient may subject the physician to liability
Canterbury v. Spence (1972)
A patient’s right of self-determination demands a standard set by law for physicians.
Truman v. Thomas (1980)
Physicians have a duty to disclose the risks of no treatment

## Slide 24
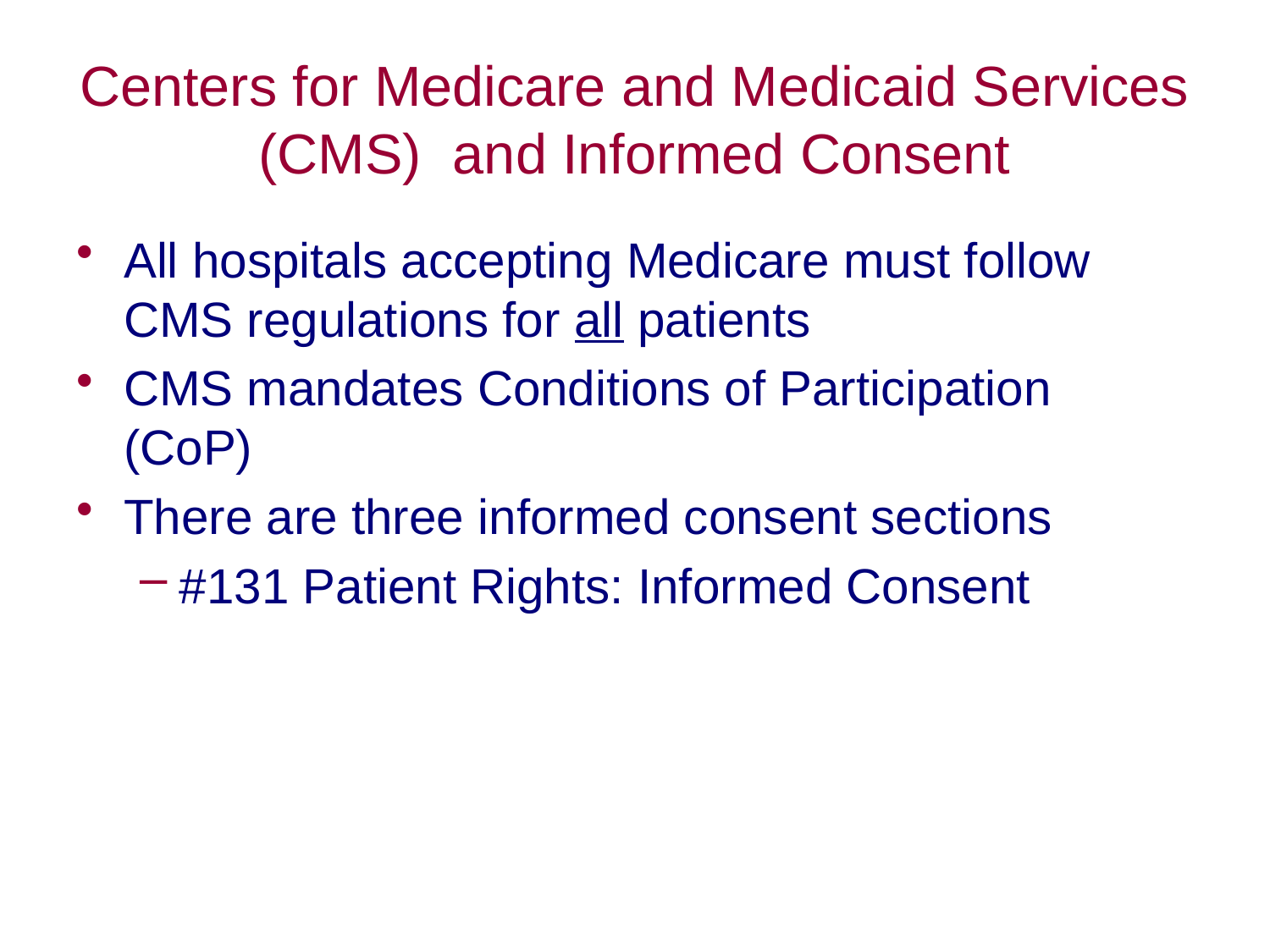

# Centers for Medicare and Medicaid Services (CMS) and Informed Consent
All hospitals accepting Medicare must follow CMS regulations for all patients
CMS mandates Conditions of Participation (CoP)
There are three informed consent sections
#131 Patient Rights: Informed Consent

## Slide 25
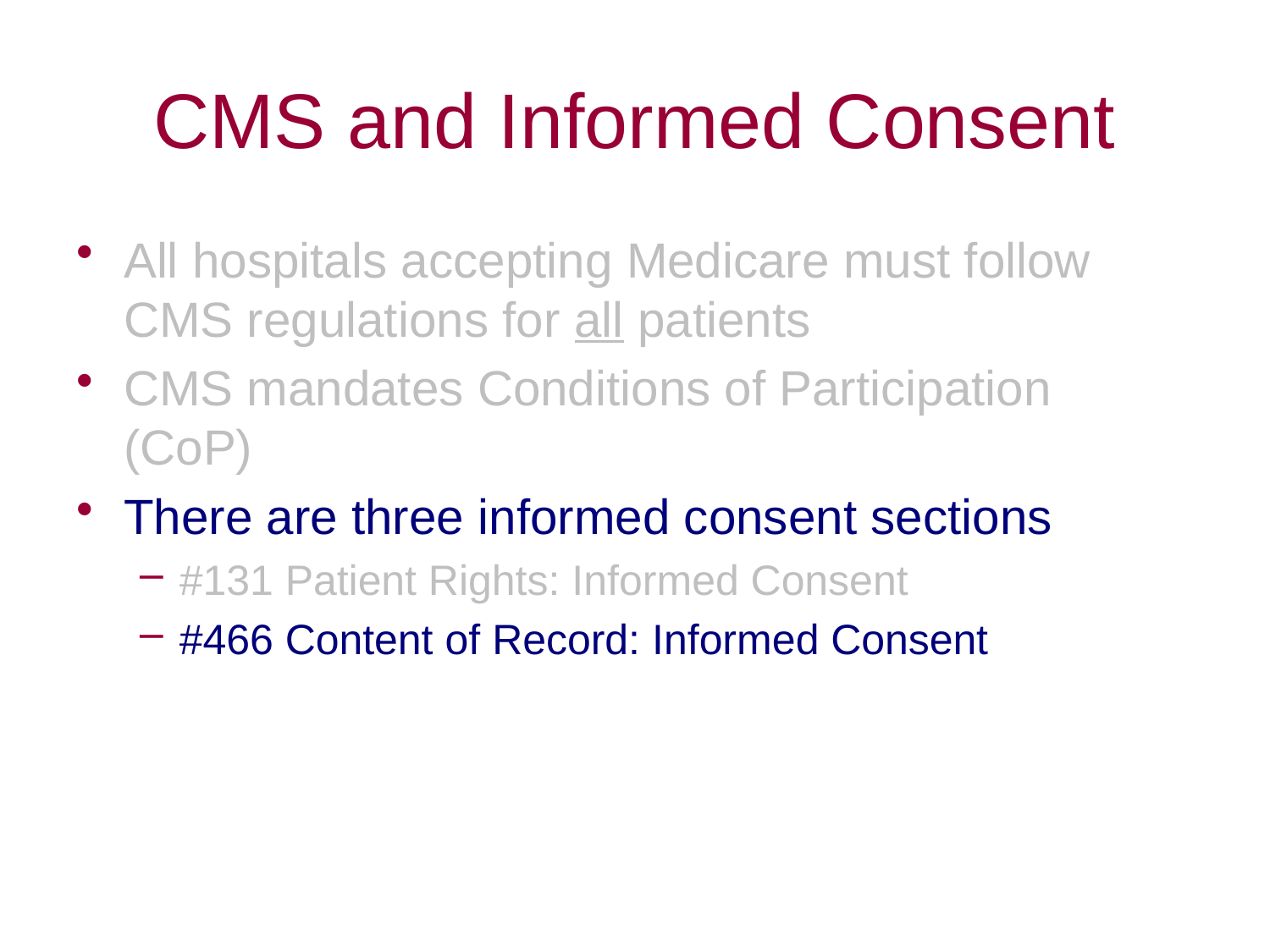

# CMS and Informed Consent
All hospitals accepting Medicare must follow CMS regulations for all patients
CMS mandates Conditions of Participation (CoP)
There are three informed consent sections
#131 Patient Rights: Informed Consent
#466 Content of Record: Informed Consent

## Slide 26
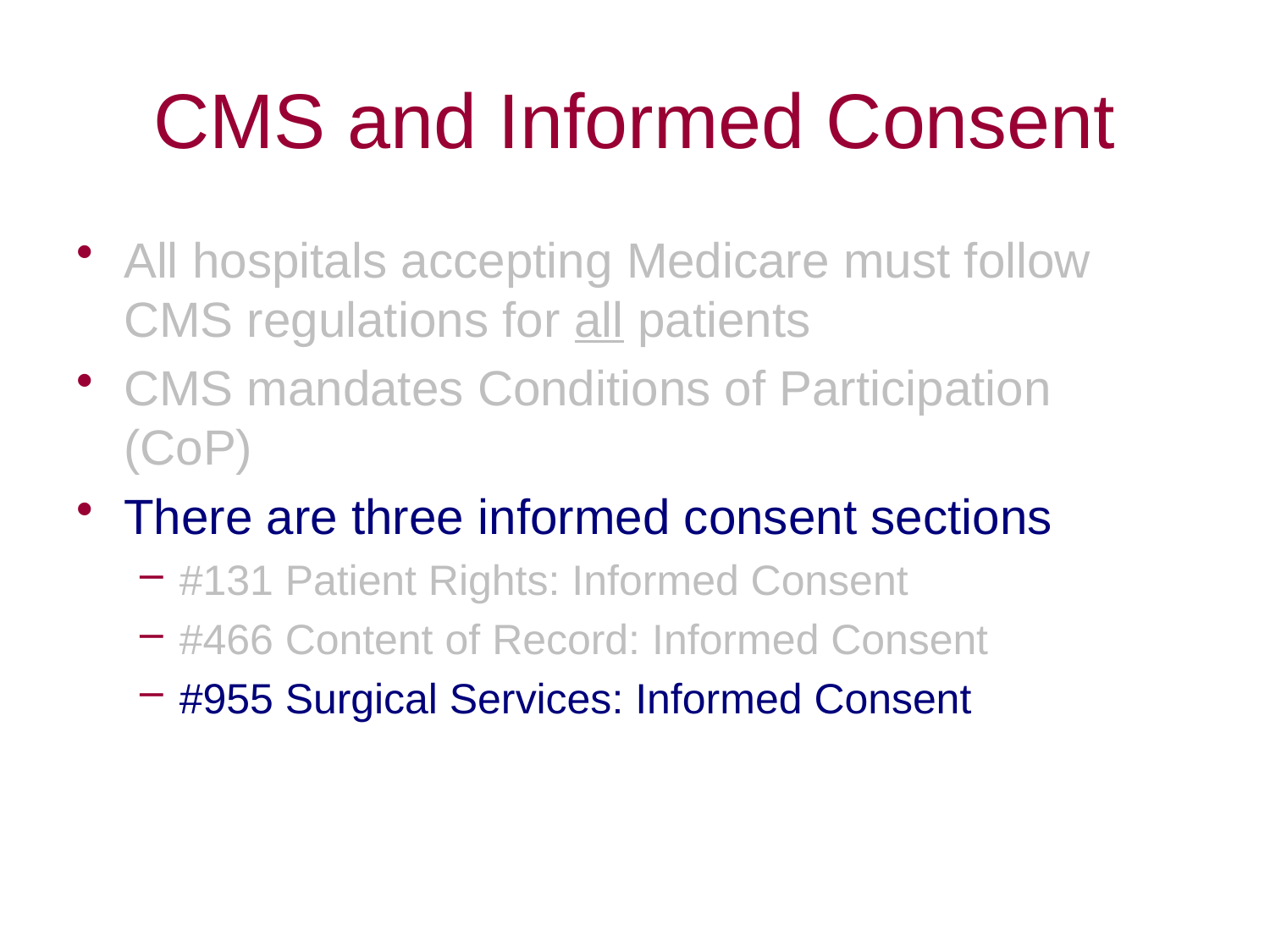

# CMS and Informed Consent
All hospitals accepting Medicare must follow CMS regulations for all patients
CMS mandates Conditions of Participation (CoP)
There are three informed consent sections
#131 Patient Rights: Informed Consent
#466 Content of Record: Informed Consent
#955 Surgical Services: Informed Consent

## Slide 27
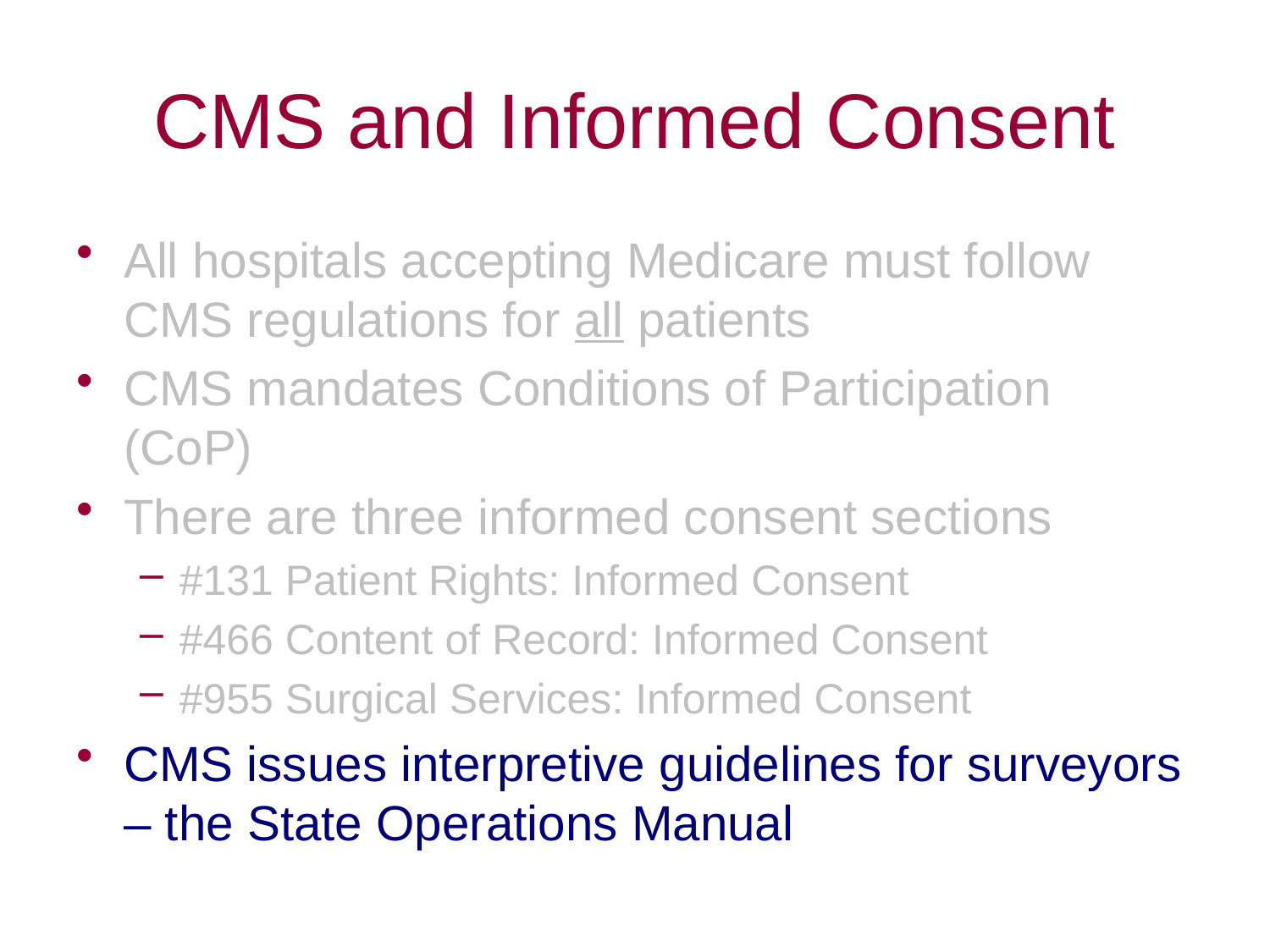

# CMS and Informed Consent
All hospitals accepting Medicare must follow CMS regulations for all patients
CMS mandates Conditions of Participation (CoP)
There are three informed consent sections
#131 Patient Rights: Informed Consent
#466 Content of Record: Informed Consent
#955 Surgical Services: Informed Consent
CMS issues interpretive guidelines for surveyors – the State Operations Manual

## Slide 28
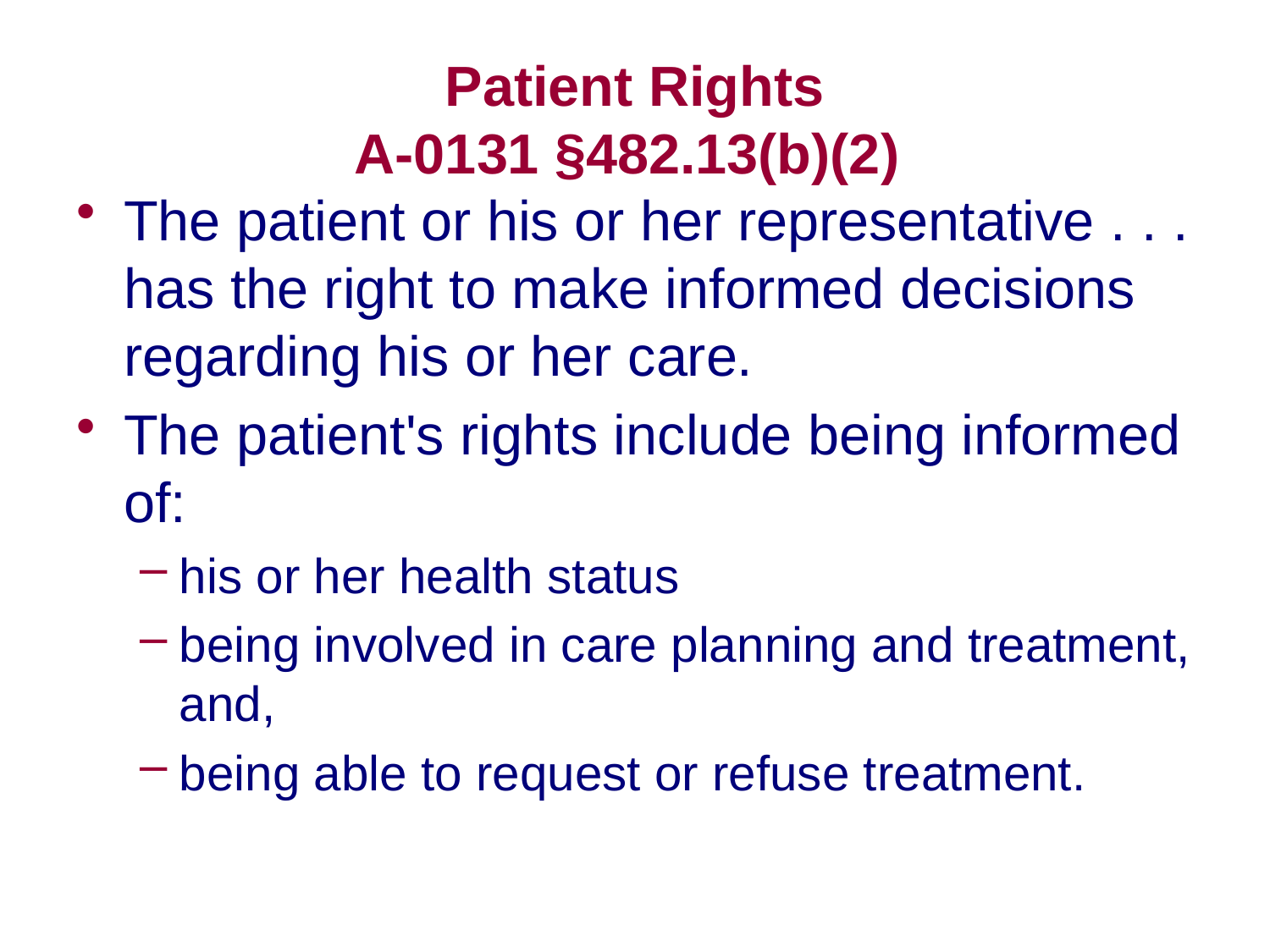

# Patient Rights A-0131 §482.13(b)(2)
The patient or his or her representative . . . has the right to make informed decisions regarding his or her care.
The patient's rights include being informed of:
his or her health status
being involved in care planning and treatment, and,
being able to request or refuse treatment.

## Slide 29
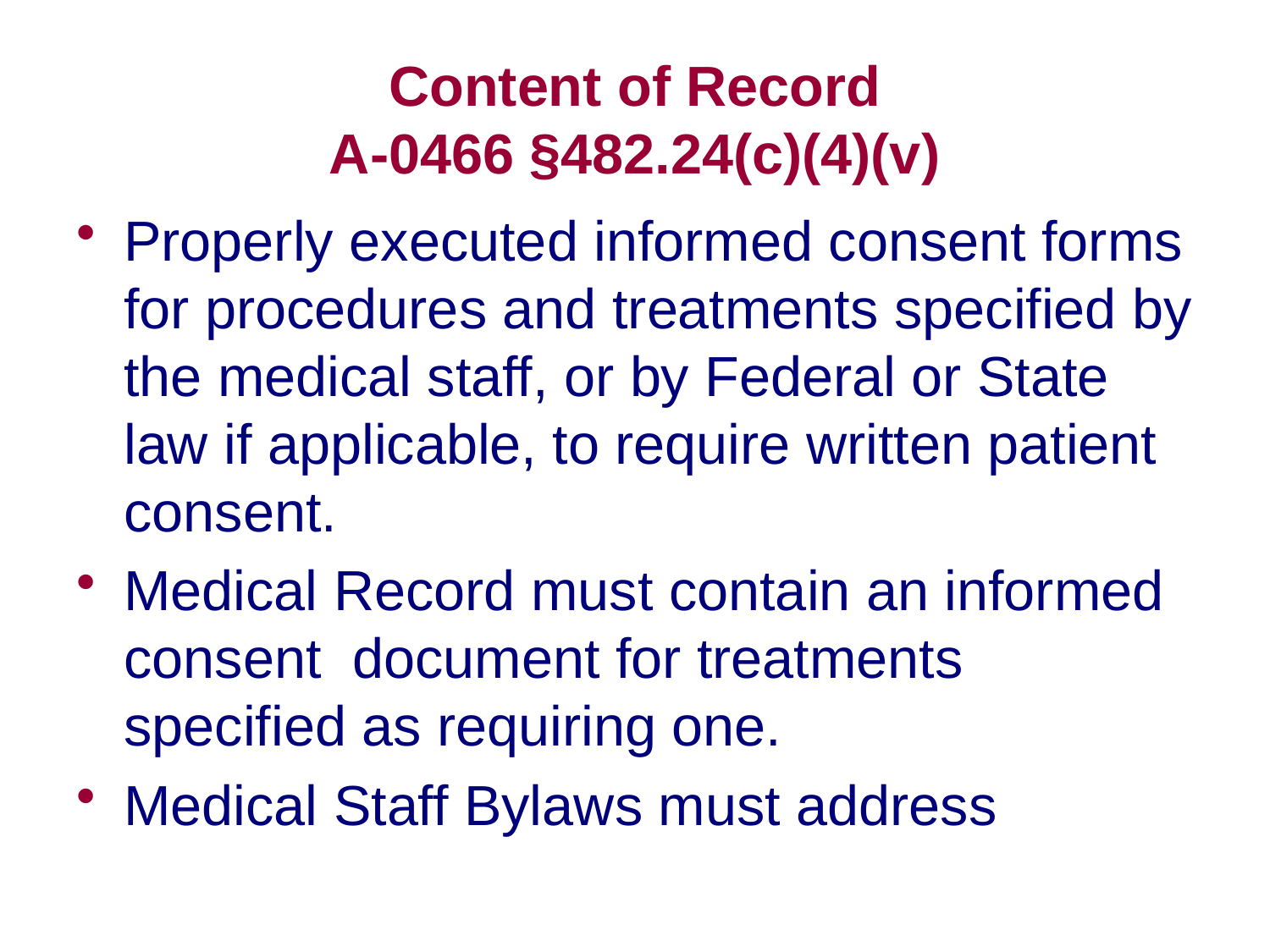

# Content of Record A-0466 §482.24(c)(4)(v)
Properly executed informed consent forms for procedures and treatments specified by the medical staff, or by Federal or State law if applicable, to require written patient consent.
Medical Record must contain an informed consent document for treatments specified as requiring one.
Medical Staff Bylaws must address

## Slide 30
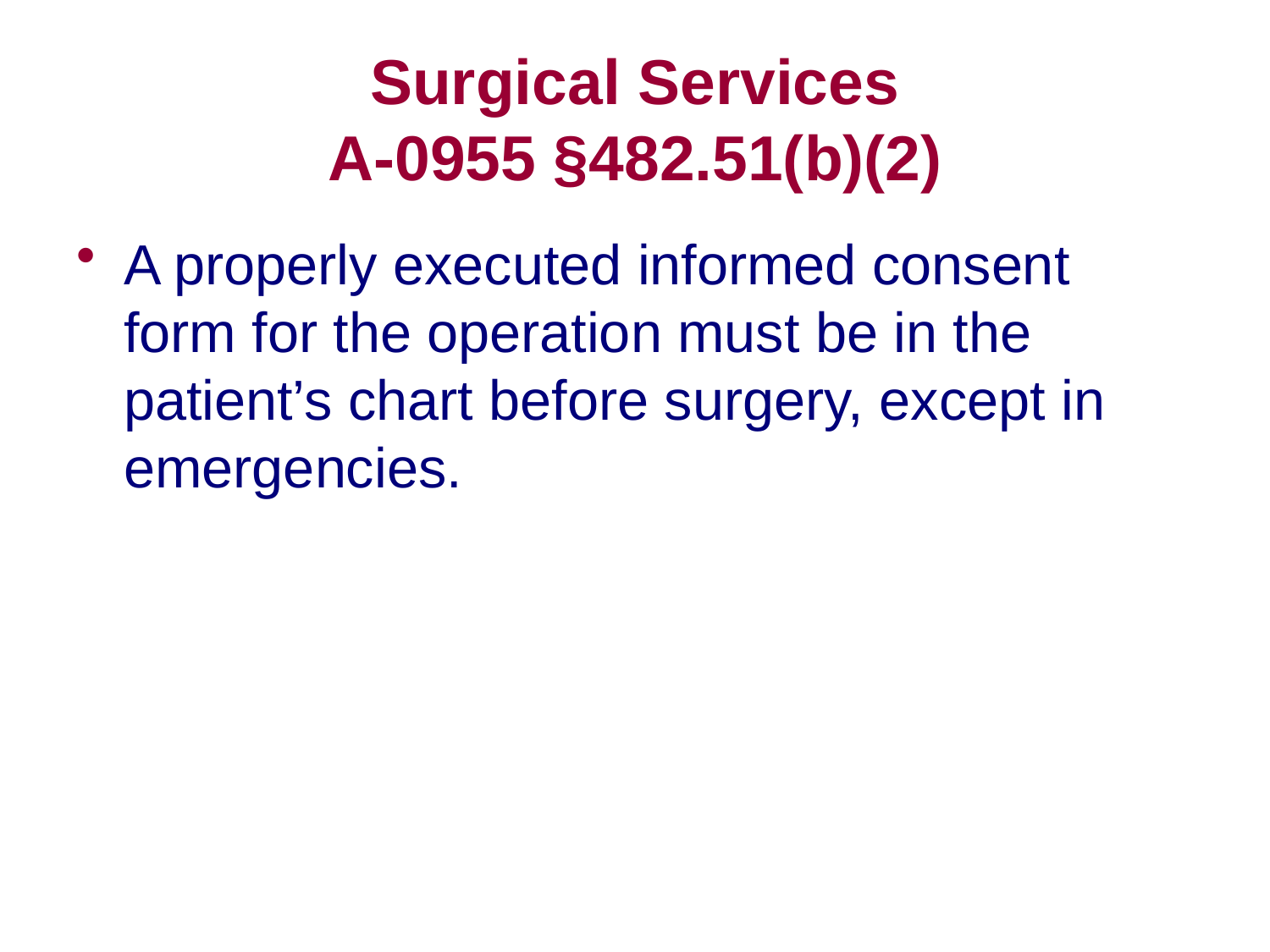

# Surgical Services A-0955 §482.51(b)(2)
A properly executed informed consent form for the operation must be in the patient’s chart before surgery, except in emergencies.

## Slide 31
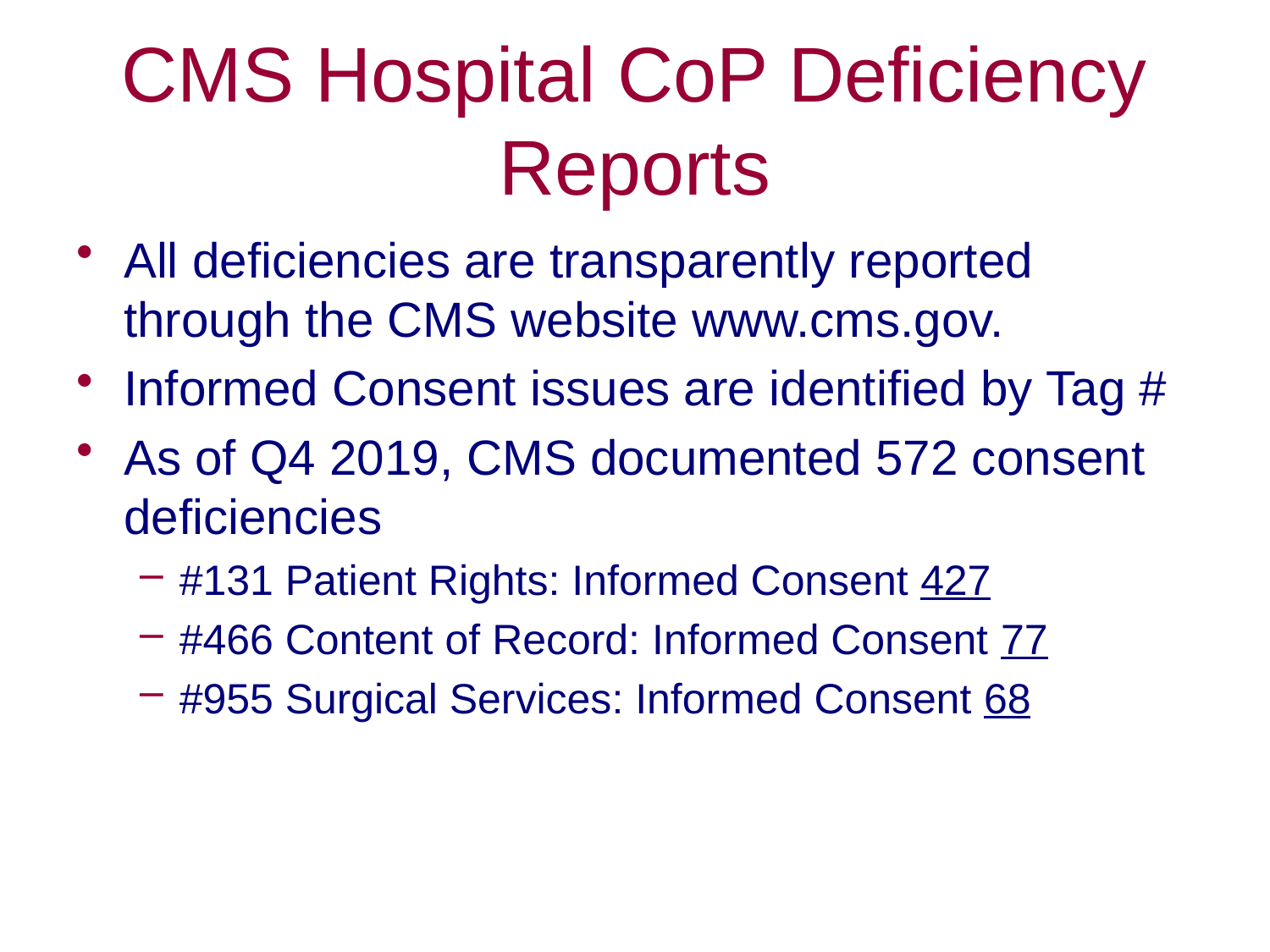

# CMS Hospital CoP Deficiency Reports
All deficiencies are transparently reported through the CMS website www.cms.gov.
Informed Consent issues are identified by Tag #
As of Q4 2019, CMS documented 572 consent deficiencies
#131 Patient Rights: Informed Consent 427
#466 Content of Record: Informed Consent 77
#955 Surgical Services: Informed Consent 68

## Slide 32
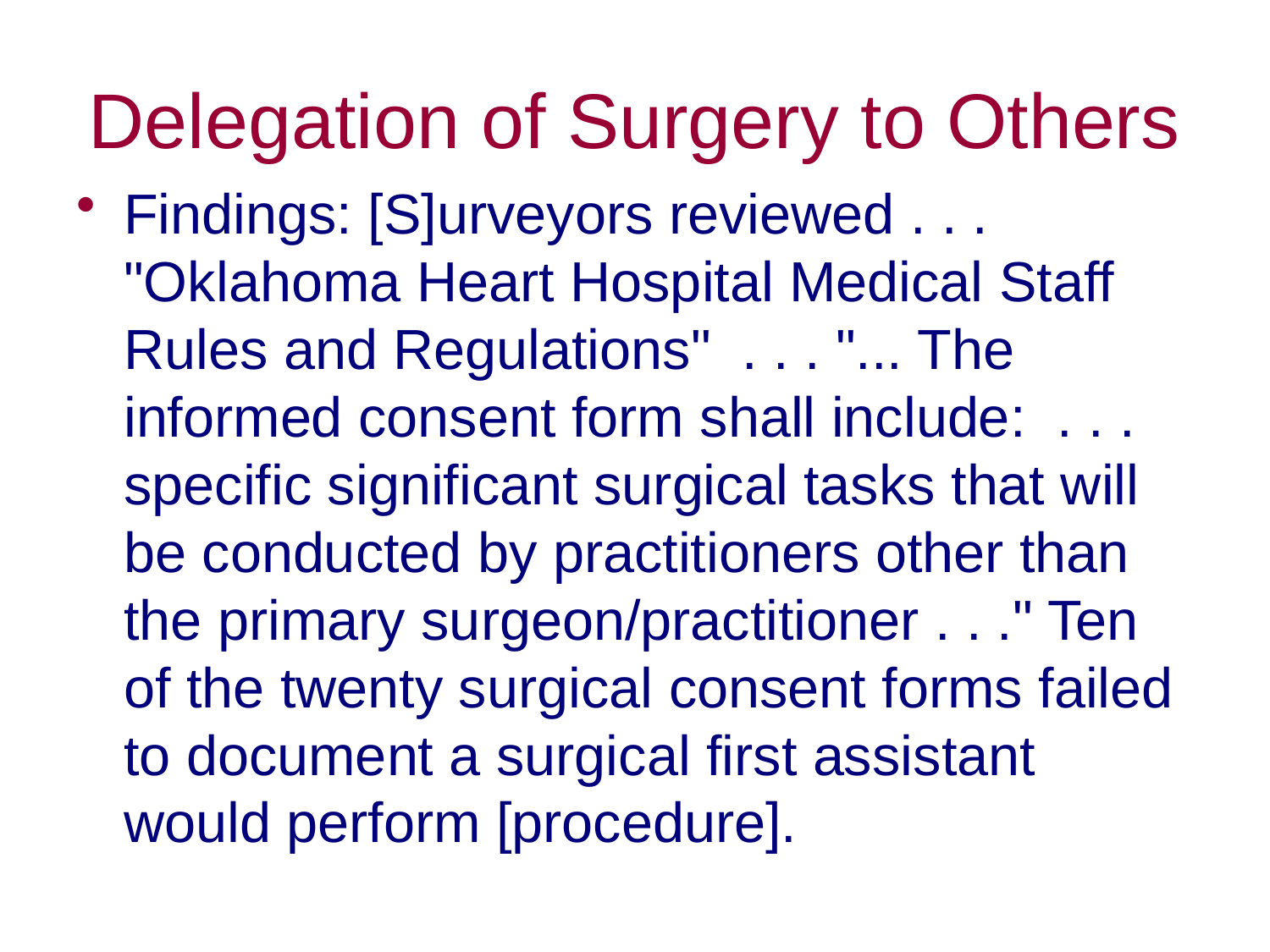

# Delegation of Surgery to Others
Findings: [S]urveyors reviewed . . . "Oklahoma Heart Hospital Medical Staff Rules and Regulations" . . . "... The informed consent form shall include: . . . specific significant surgical tasks that will be conducted by practitioners other than the primary surgeon/practitioner . . ." Ten of the twenty surgical consent forms failed to document a surgical first assistant would perform [procedure].

## Slide 33
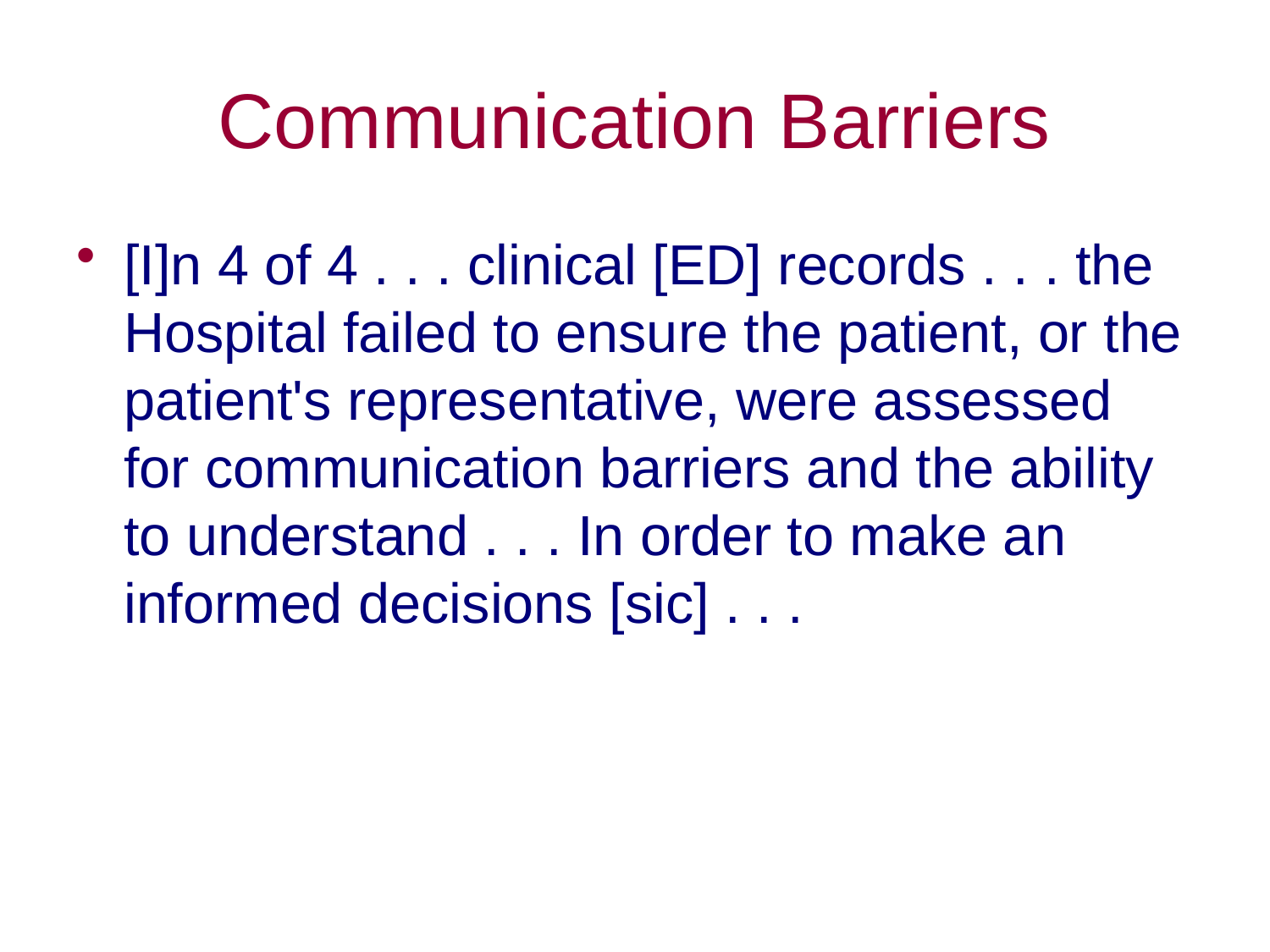

# Communication Barriers
[I]n 4 of 4 . . . clinical [ED] records . . . the Hospital failed to ensure the patient, or the patient's representative, were assessed for communication barriers and the ability to understand . . . In order to make an informed decisions [sic] . . .

## Slide 34
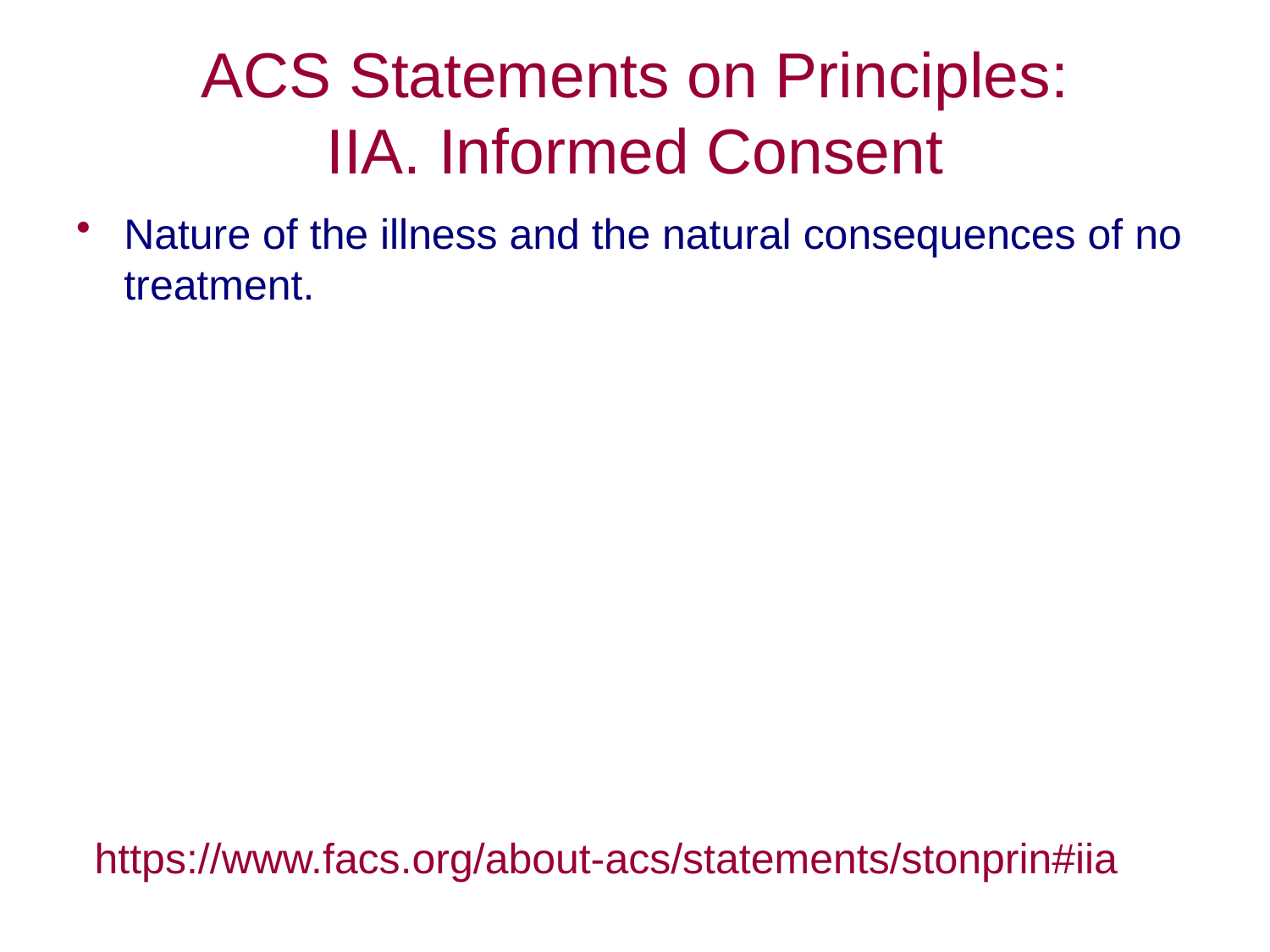

# ACS Statements on Principles: IIA. Informed Consent
Nature of the illness and the natural consequences of no treatment.
https://www.facs.org/about-acs/statements/stonprin#iia

## Slide 35
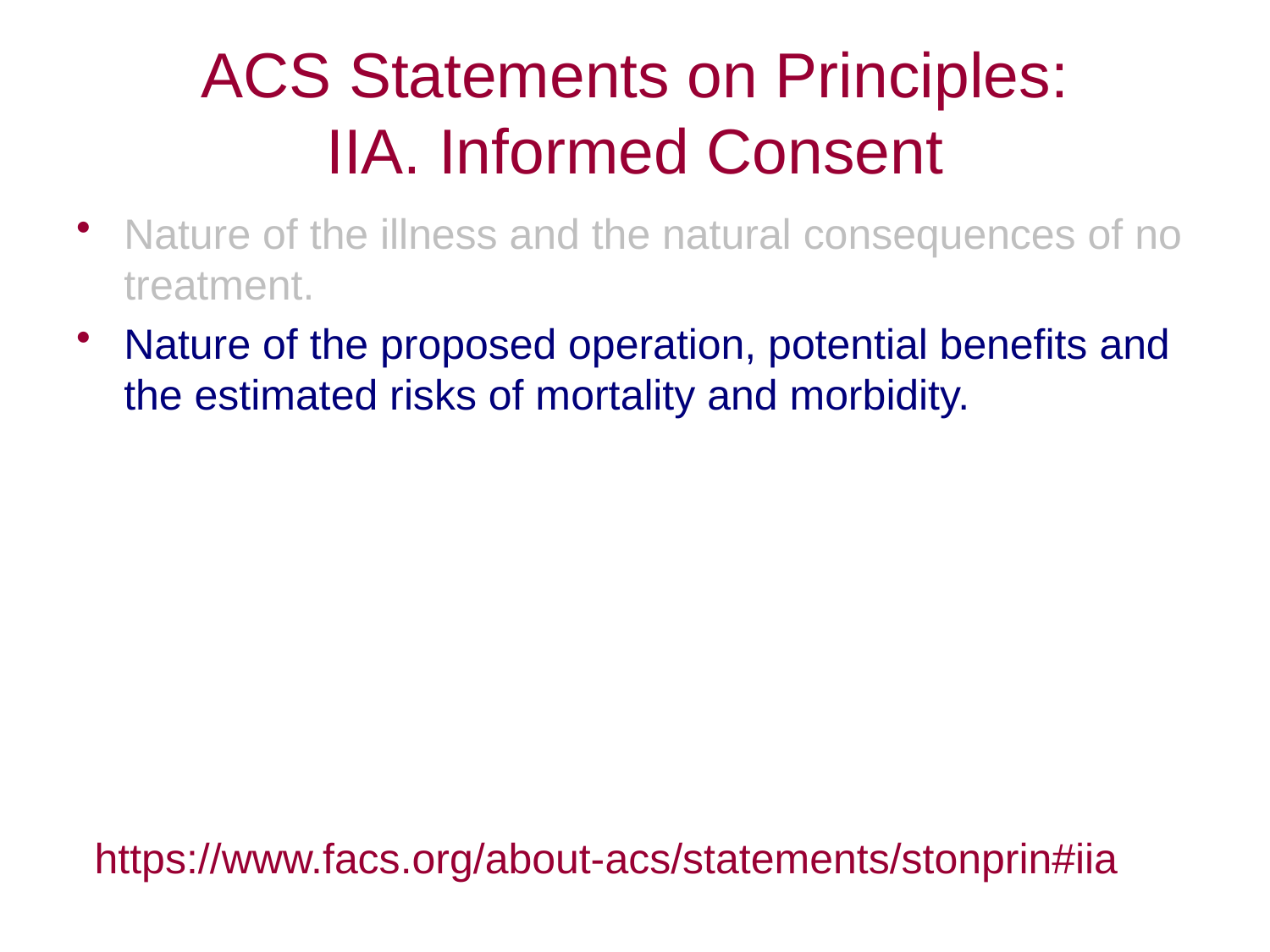

# ACS Statements on Principles: IIA. Informed Consent
Nature of the illness and the natural consequences of no treatment.
Nature of the proposed operation, potential benefits and the estimated risks of mortality and morbidity.
https://www.facs.org/about-acs/statements/stonprin#iia

## Slide 36
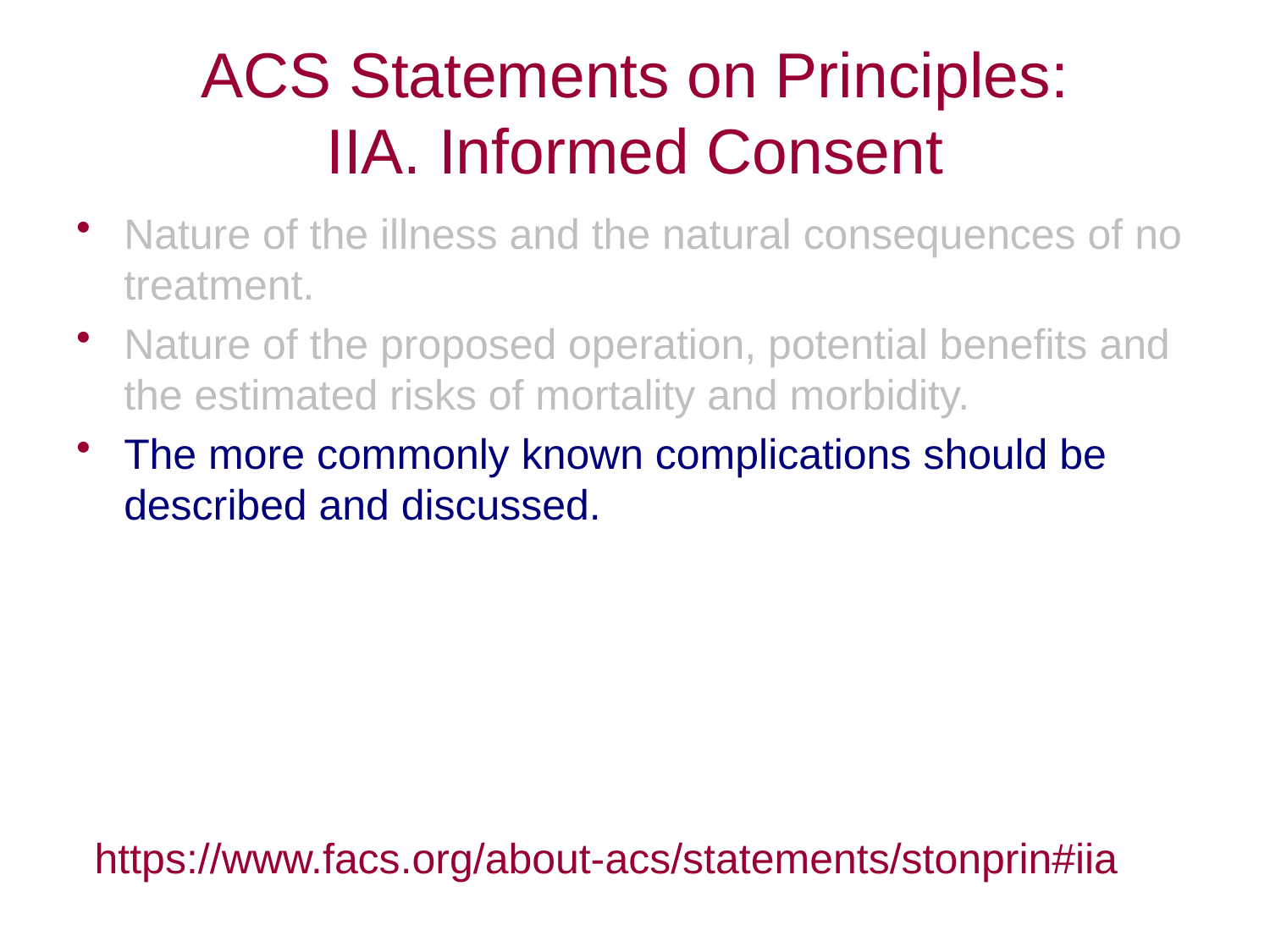

# ACS Statements on Principles: IIA. Informed Consent
Nature of the illness and the natural consequences of no treatment.
Nature of the proposed operation, potential benefits and the estimated risks of mortality and morbidity.
The more commonly known complications should be described and discussed.
https://www.facs.org/about-acs/statements/stonprin#iia

## Slide 37
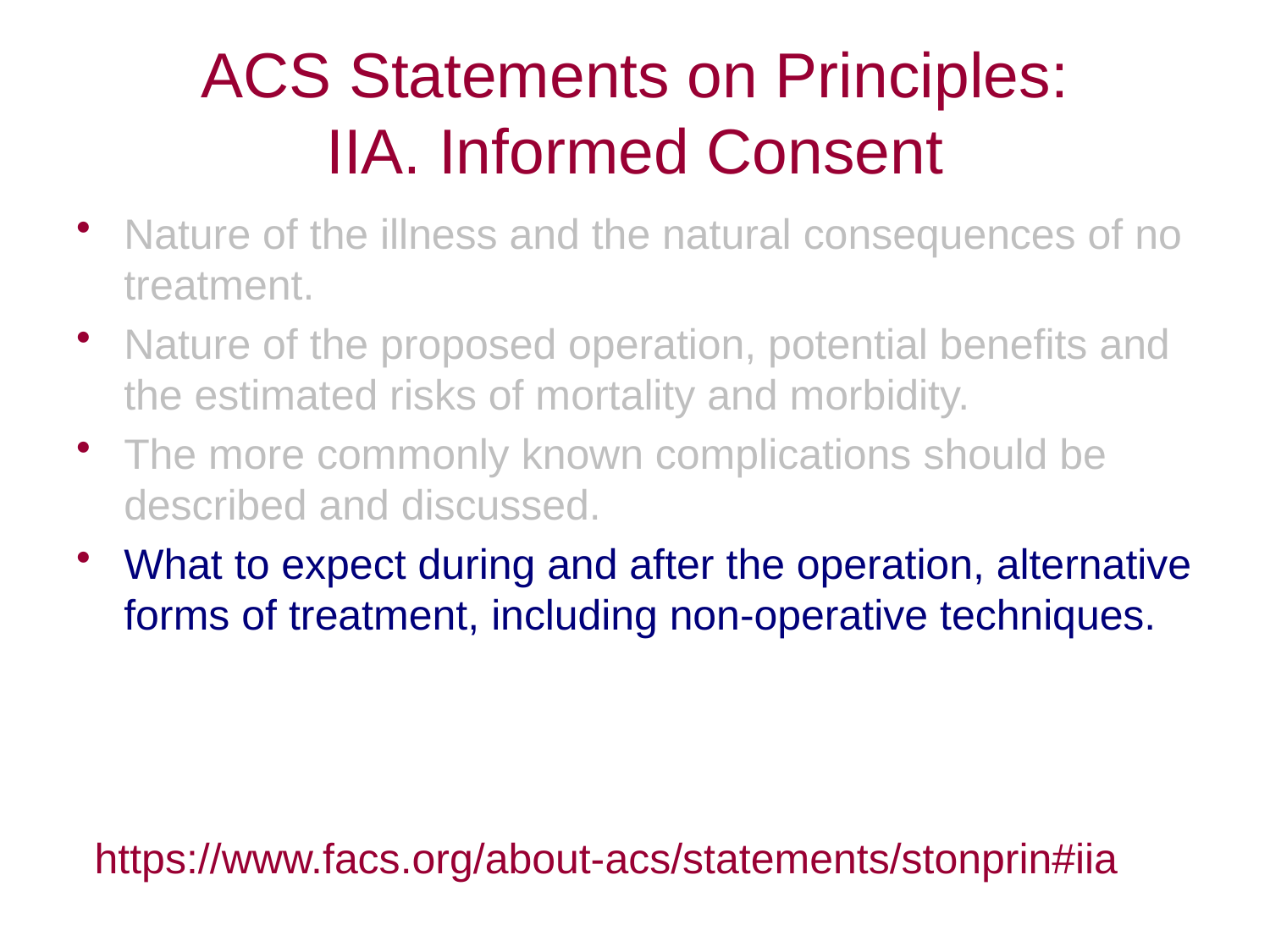

# ACS Statements on Principles: IIA. Informed Consent
Nature of the illness and the natural consequences of no treatment.
Nature of the proposed operation, potential benefits and the estimated risks of mortality and morbidity.
The more commonly known complications should be described and discussed.
What to expect during and after the operation, alternative forms of treatment, including non-operative techniques.
https://www.facs.org/about-acs/statements/stonprin#iia

## Slide 38
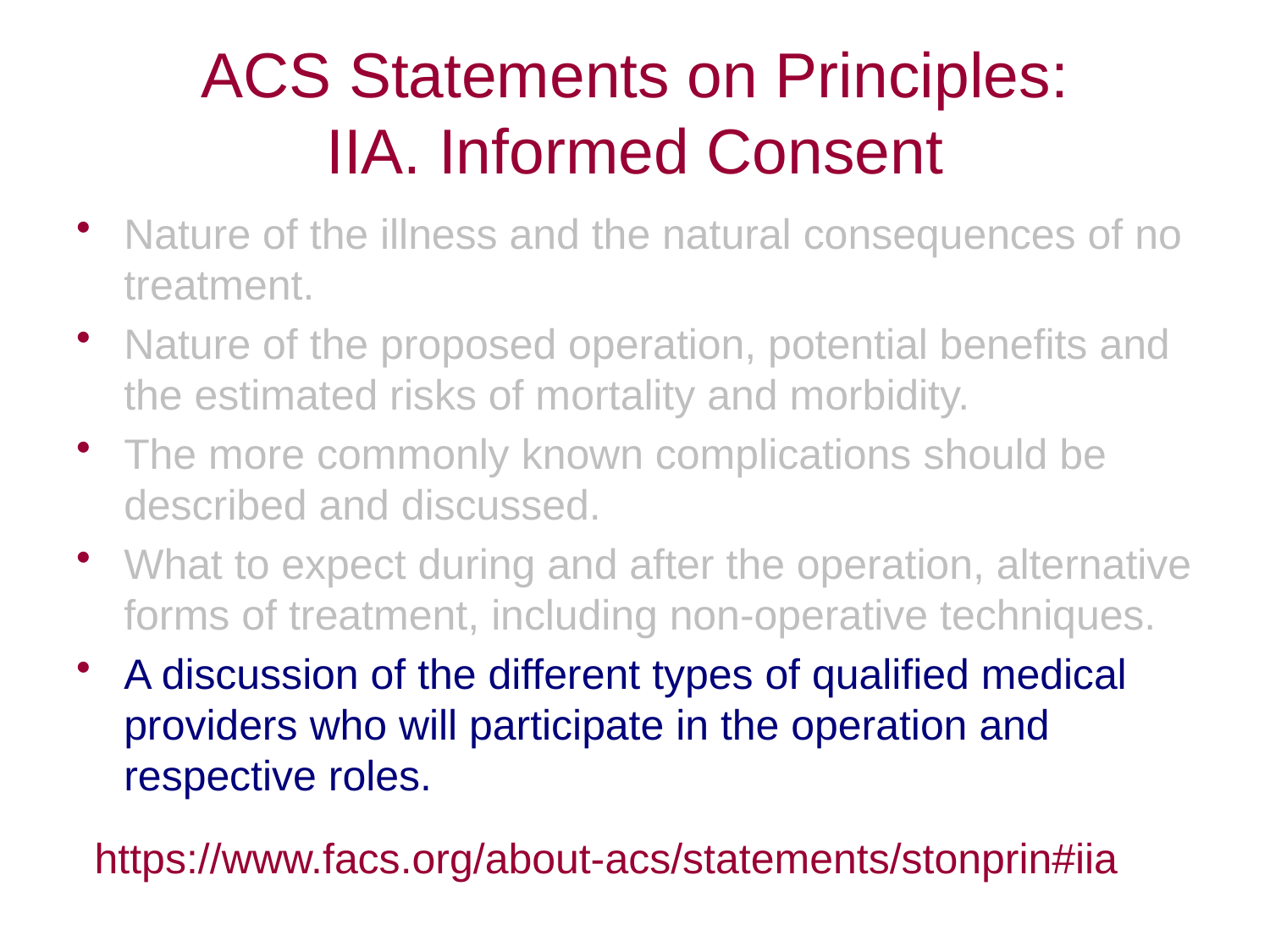

# ACS Statements on Principles: IIA. Informed Consent
Nature of the illness and the natural consequences of no treatment.
Nature of the proposed operation, potential benefits and the estimated risks of mortality and morbidity.
The more commonly known complications should be described and discussed.
What to expect during and after the operation, alternative forms of treatment, including non-operative techniques.
A discussion of the different types of qualified medical providers who will participate in the operation and respective roles.
https://www.facs.org/about-acs/statements/stonprin#iia

## Slide 39
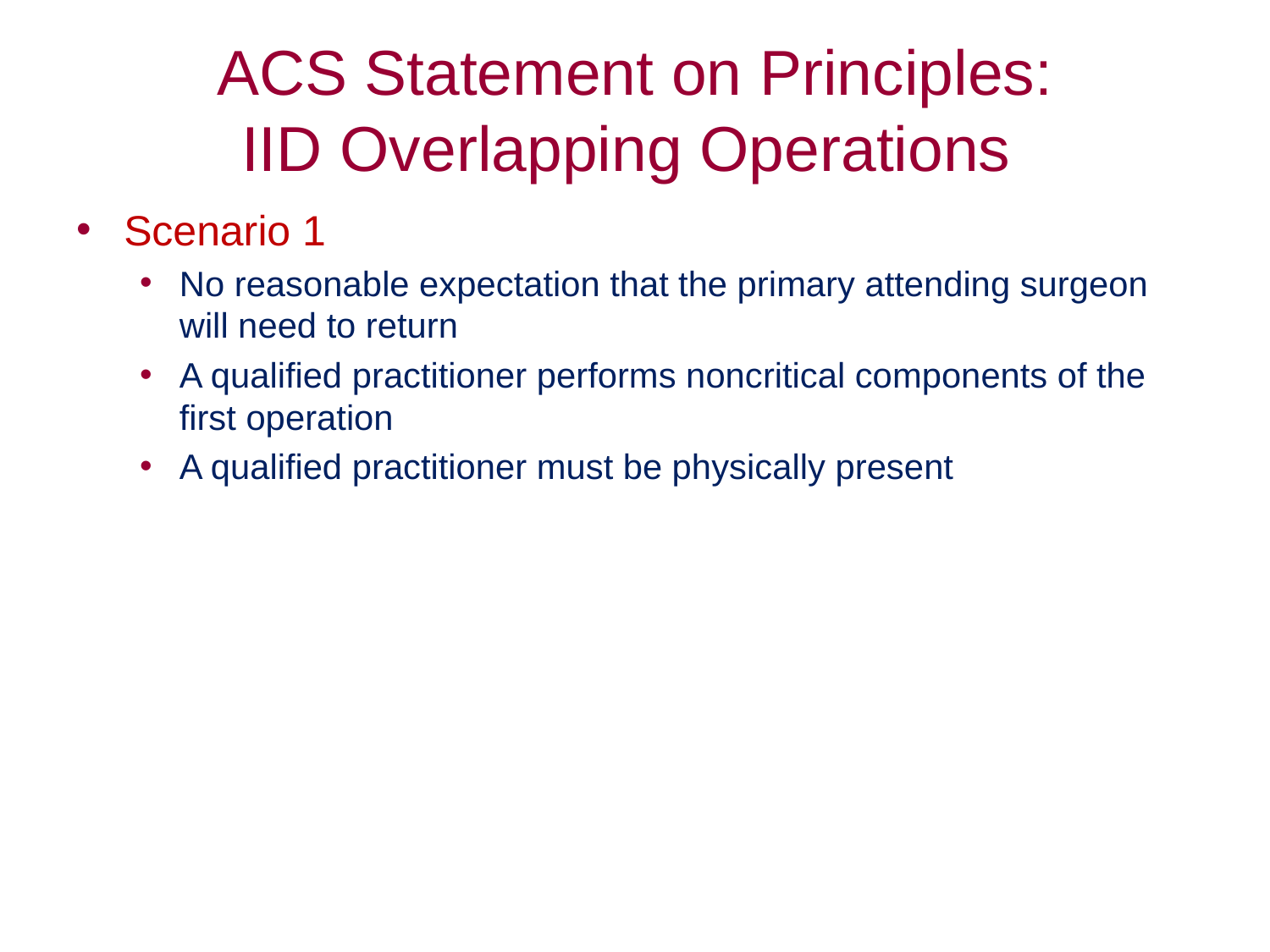

# ACS Statement on Principles: IID Overlapping Operations
Scenario 1
No reasonable expectation that the primary attending surgeon will need to return
A qualified practitioner performs noncritical components of the first operation
A qualified practitioner must be physically present

## Slide 40
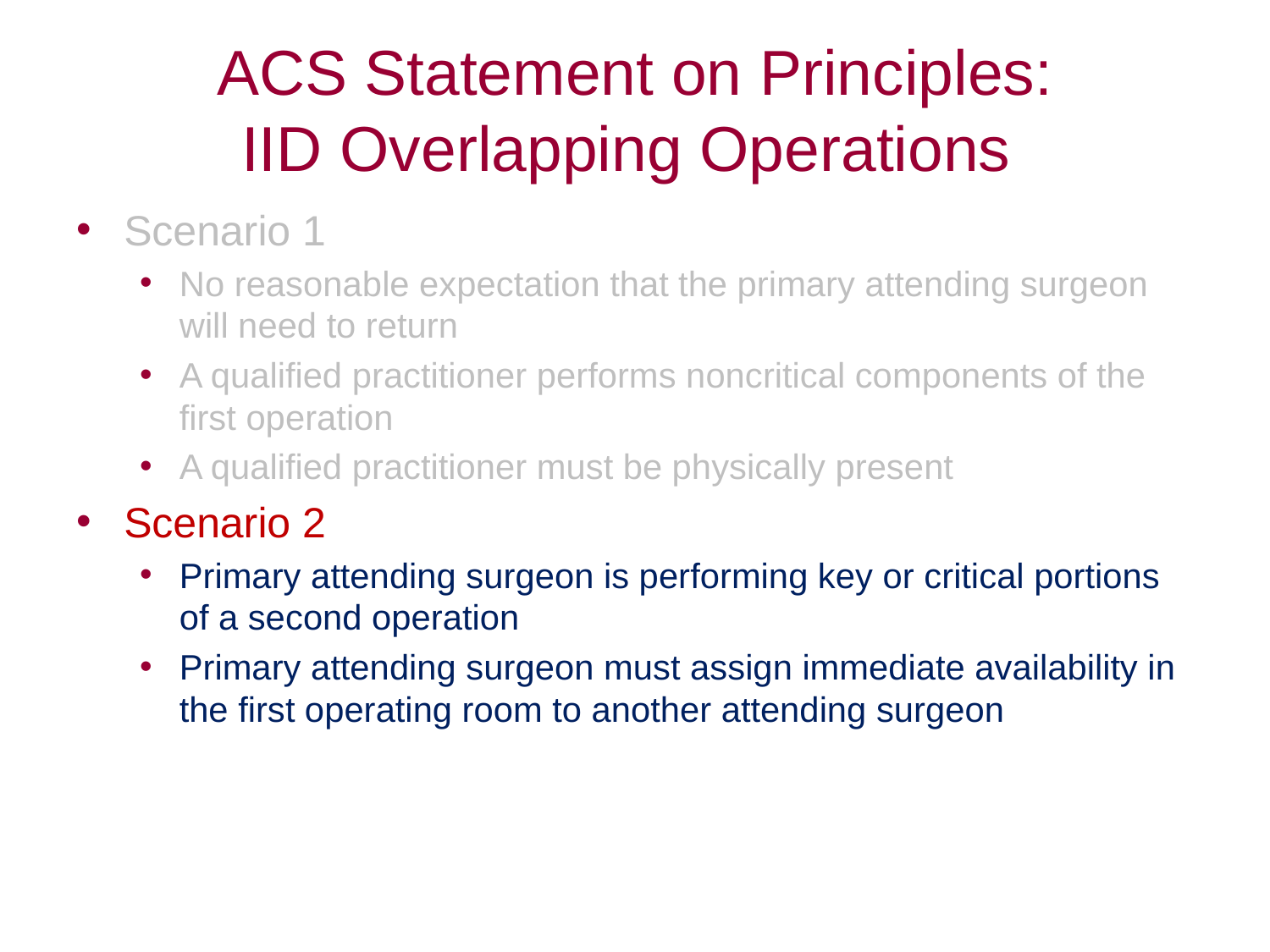

# ACS Statement on Principles: IID Overlapping Operations
Scenario 1
No reasonable expectation that the primary attending surgeon will need to return
A qualified practitioner performs noncritical components of the first operation
A qualified practitioner must be physically present
Scenario 2
Primary attending surgeon is performing key or critical portions of a second operation
Primary attending surgeon must assign immediate availability in the first operating room to another attending surgeon

## Slide 41
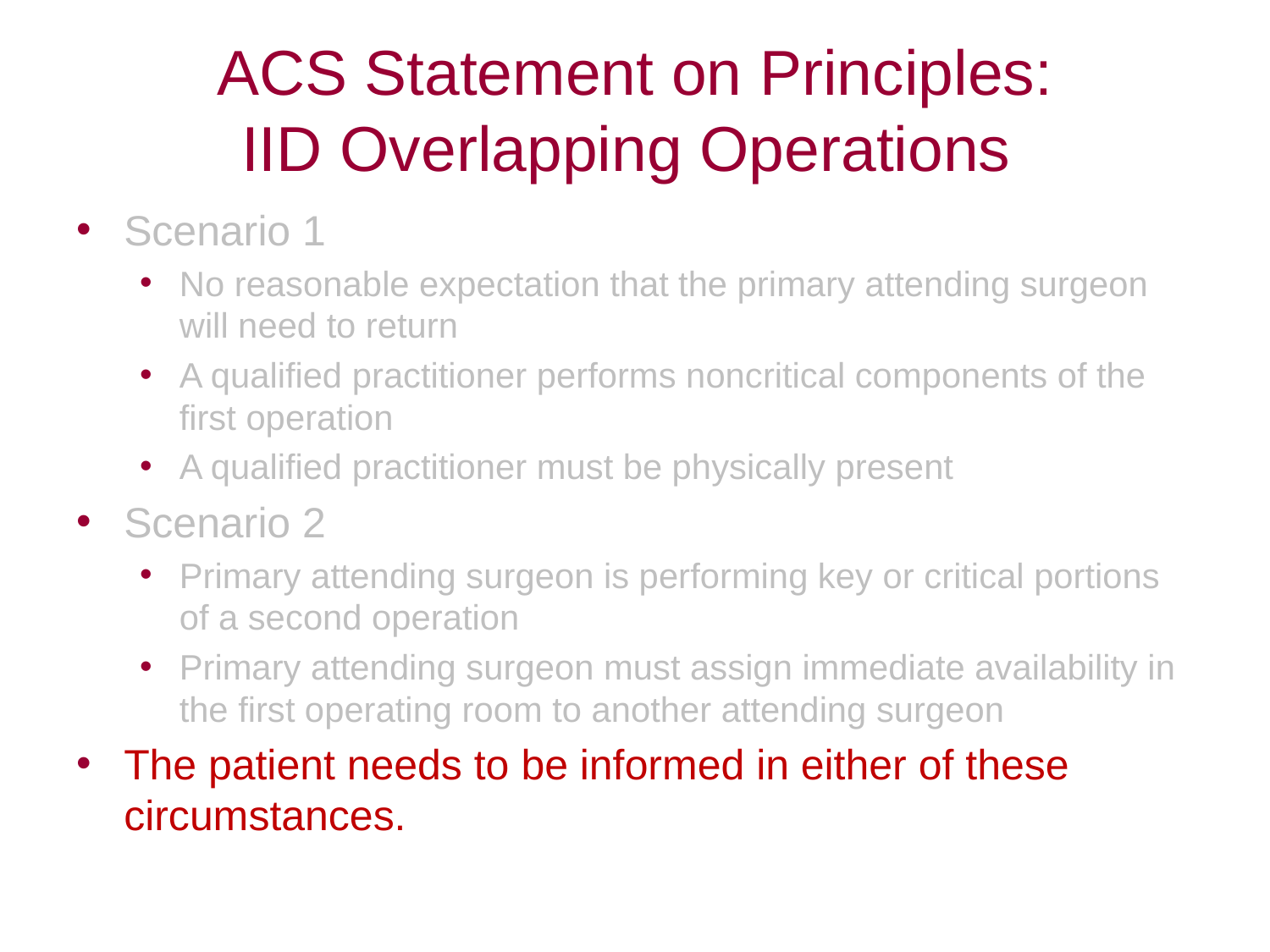

# ACS Statement on Principles: IID Overlapping Operations
Scenario 1
No reasonable expectation that the primary attending surgeon will need to return
A qualified practitioner performs noncritical components of the first operation
A qualified practitioner must be physically present
Scenario 2
Primary attending surgeon is performing key or critical portions of a second operation
Primary attending surgeon must assign immediate availability in the first operating room to another attending surgeon
The patient needs to be informed in either of these circumstances.

## Slide 42
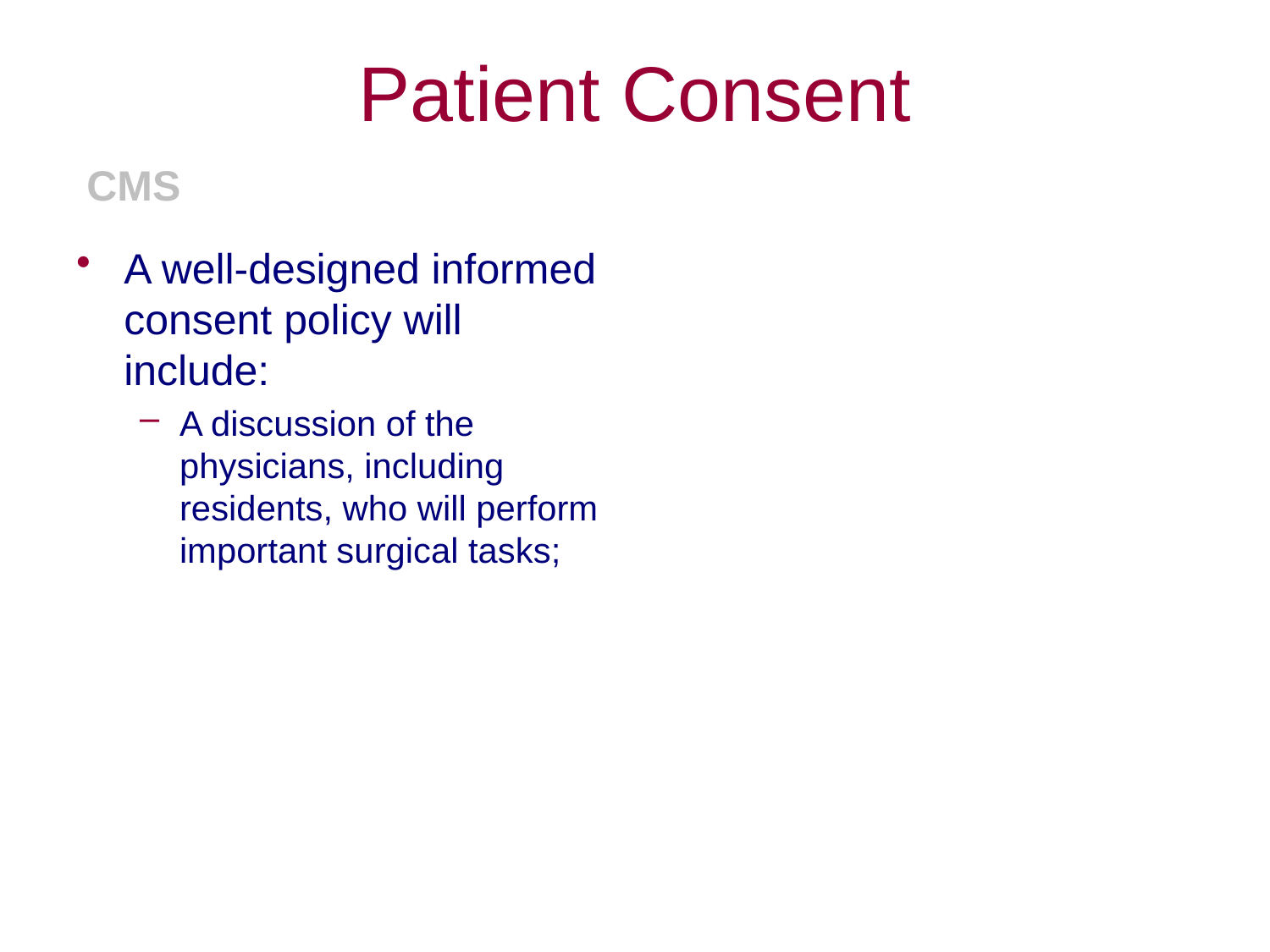

# Patient Consent
CMS
A well-designed informed consent policy will include:
A discussion of the physicians, including residents, who will perform important surgical tasks;

## Slide 43
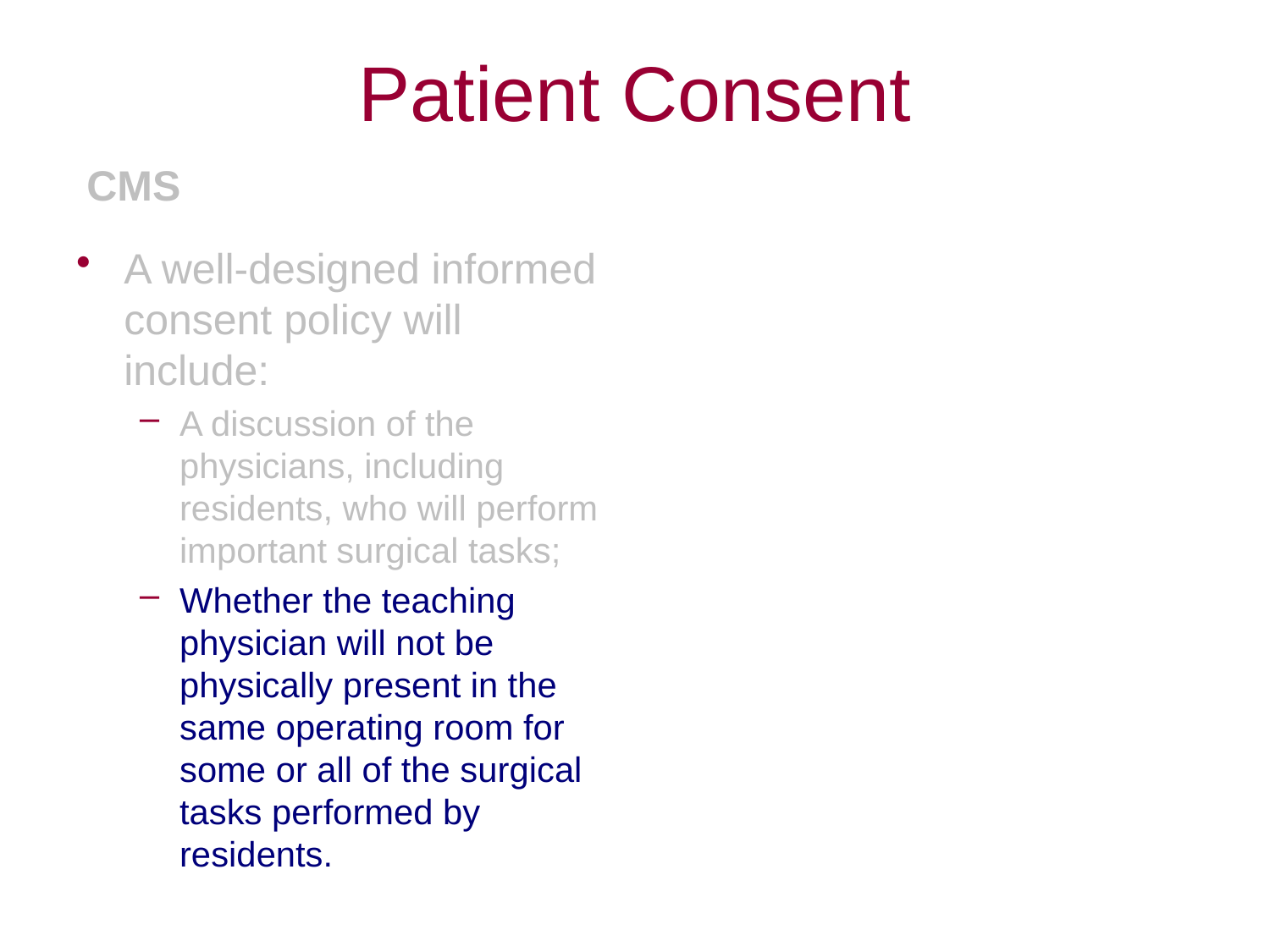

# Patient Consent
CMS
A well-designed informed consent policy will include:
A discussion of the physicians, including residents, who will perform important surgical tasks;
Whether the teaching physician will not be physically present in the same operating room for some or all of the surgical tasks performed by residents.

## Slide 44
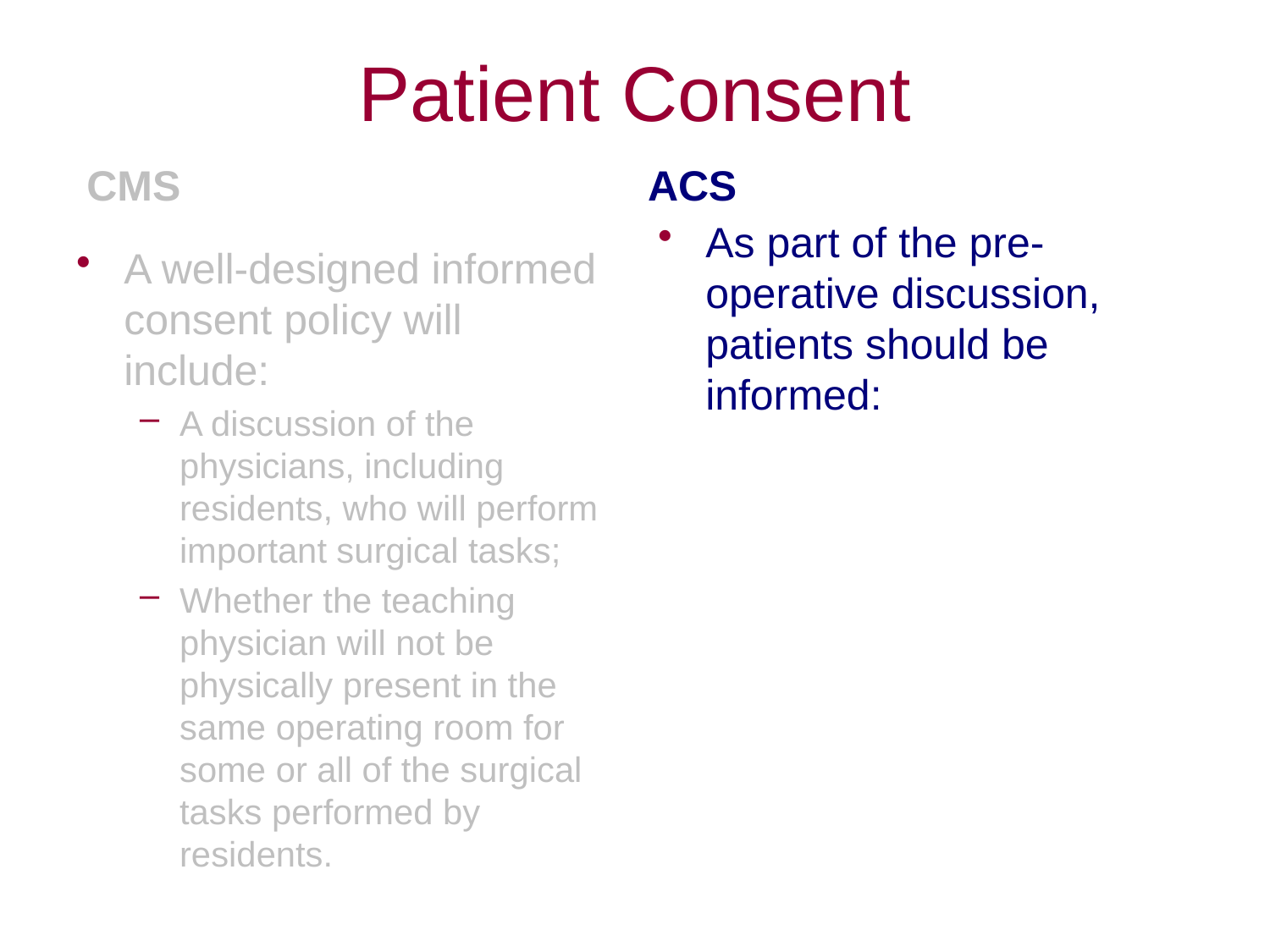

# Patient Consent
CMS
ACS
As part of the pre-operative discussion, patients should be informed:
A well-designed informed consent policy will include:
A discussion of the physicians, including residents, who will perform important surgical tasks;
Whether the teaching physician will not be physically present in the same operating room for some or all of the surgical tasks performed by residents.

## Slide 45
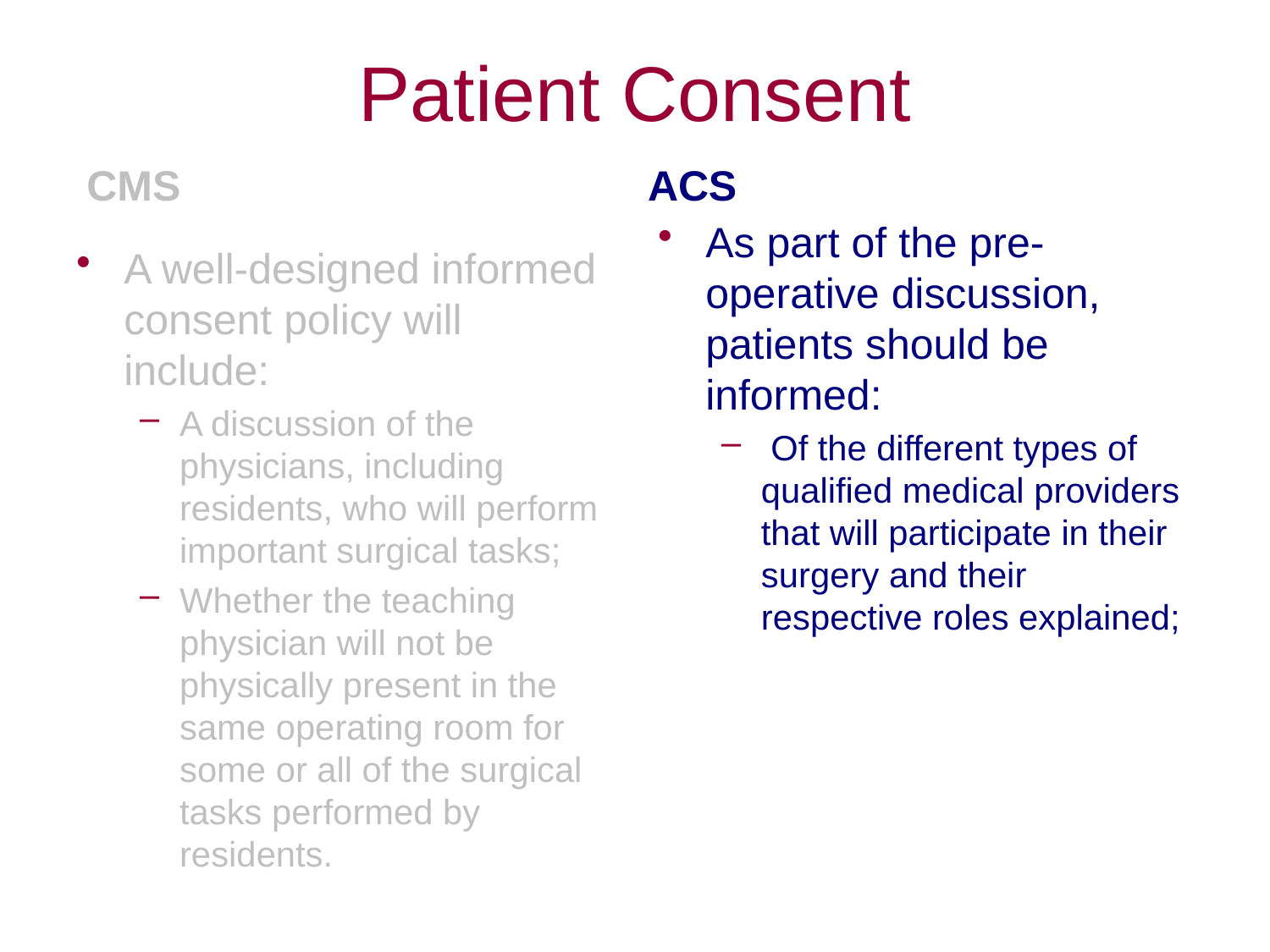

# Patient Consent
CMS
ACS
As part of the pre-operative discussion, patients should be informed:
 Of the different types of qualified medical providers that will participate in their surgery and their respective roles explained;
A well-designed informed consent policy will include:
A discussion of the physicians, including residents, who will perform important surgical tasks;
Whether the teaching physician will not be physically present in the same operating room for some or all of the surgical tasks performed by residents.

## Slide 46
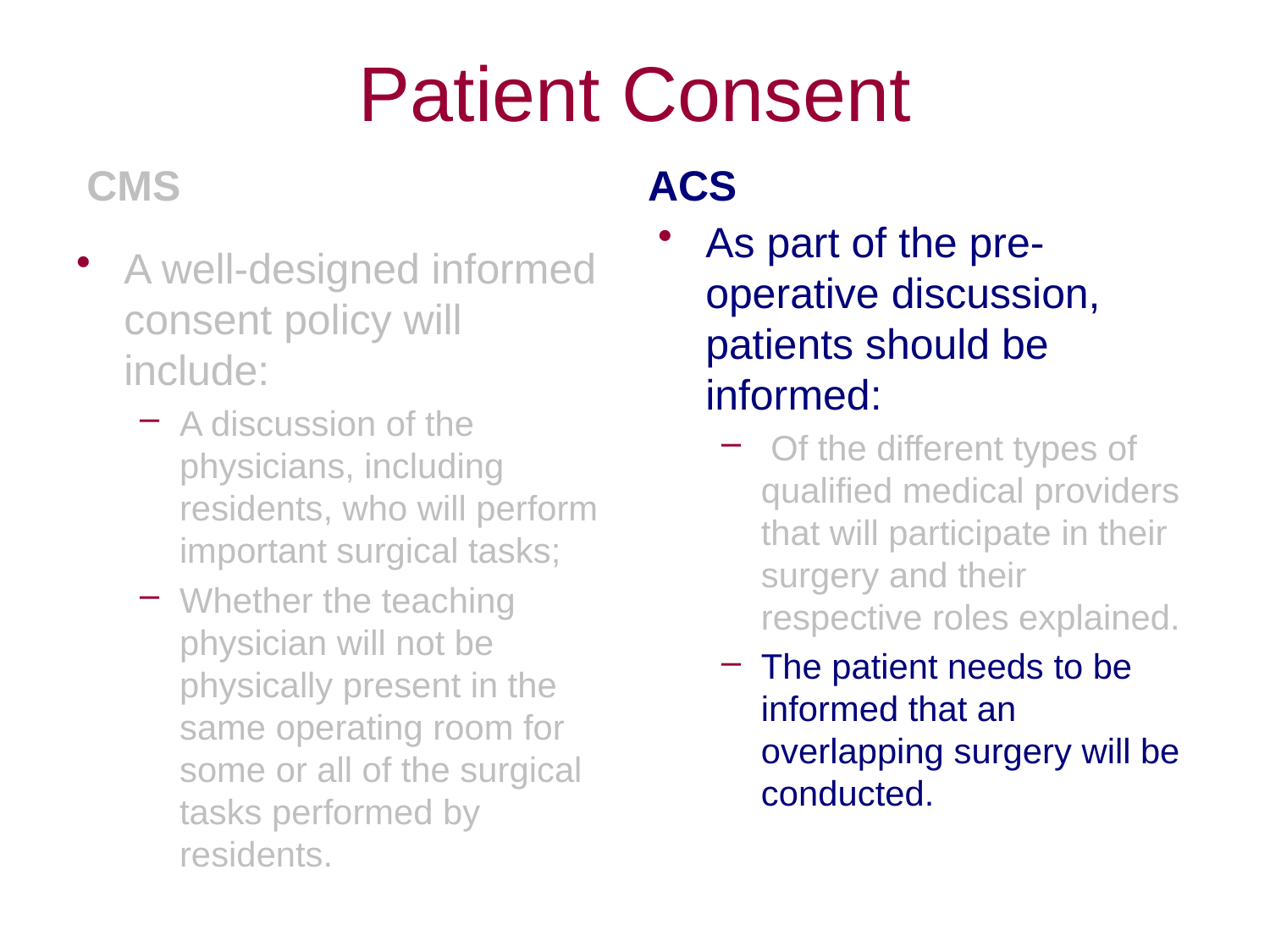

# Patient Consent
CMS
ACS
As part of the pre-operative discussion, patients should be informed:
 Of the different types of qualified medical providers that will participate in their surgery and their respective roles explained.
The patient needs to be informed that an overlapping surgery will be conducted.
A well-designed informed consent policy will include:
A discussion of the physicians, including residents, who will perform important surgical tasks;
Whether the teaching physician will not be physically present in the same operating room for some or all of the surgical tasks performed by residents.

## Slide 47
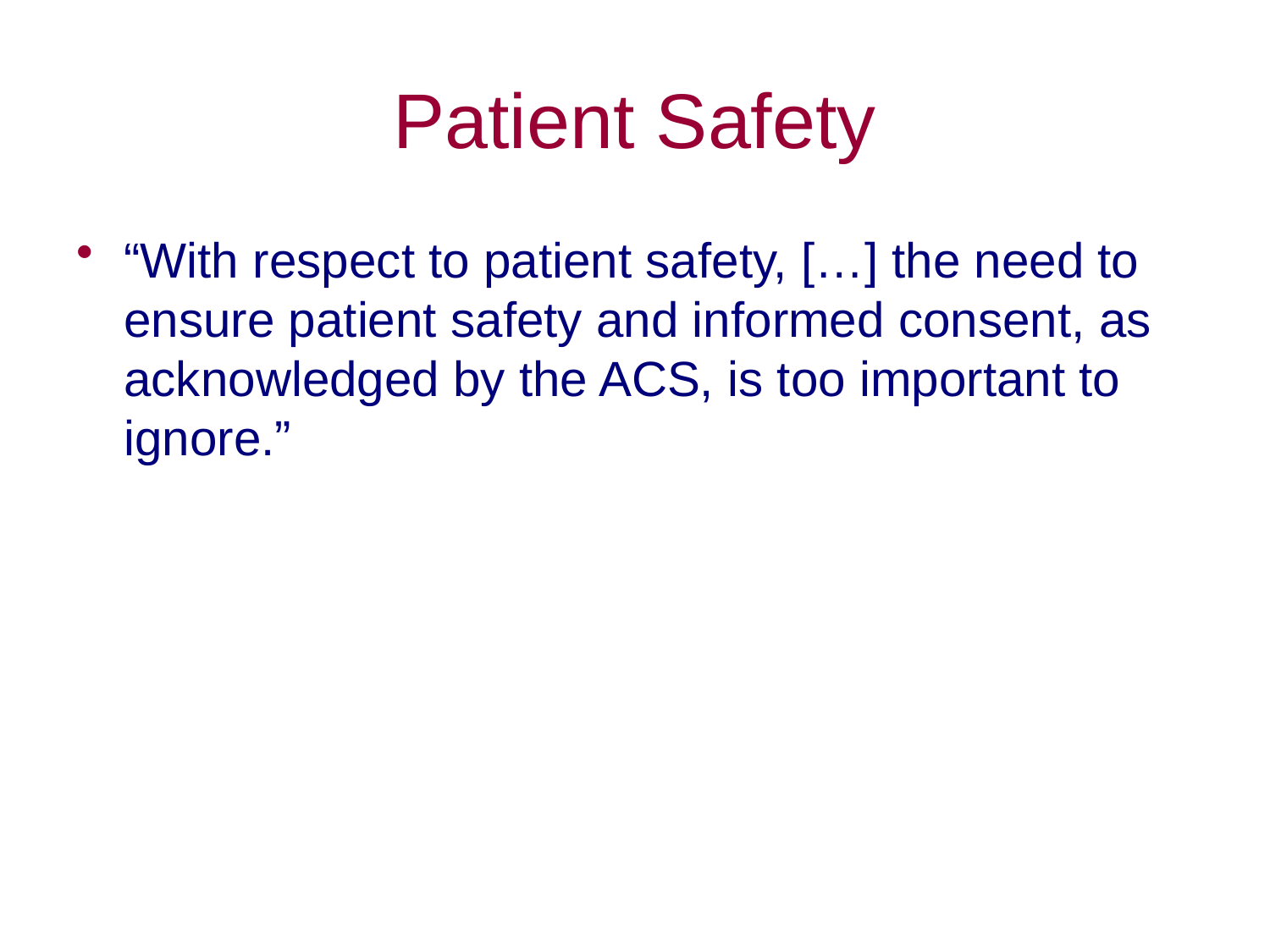

# Patient Safety
“With respect to patient safety, […] the need to ensure patient safety and informed consent, as acknowledged by the ACS, is too important to ignore.”

## Slide 48
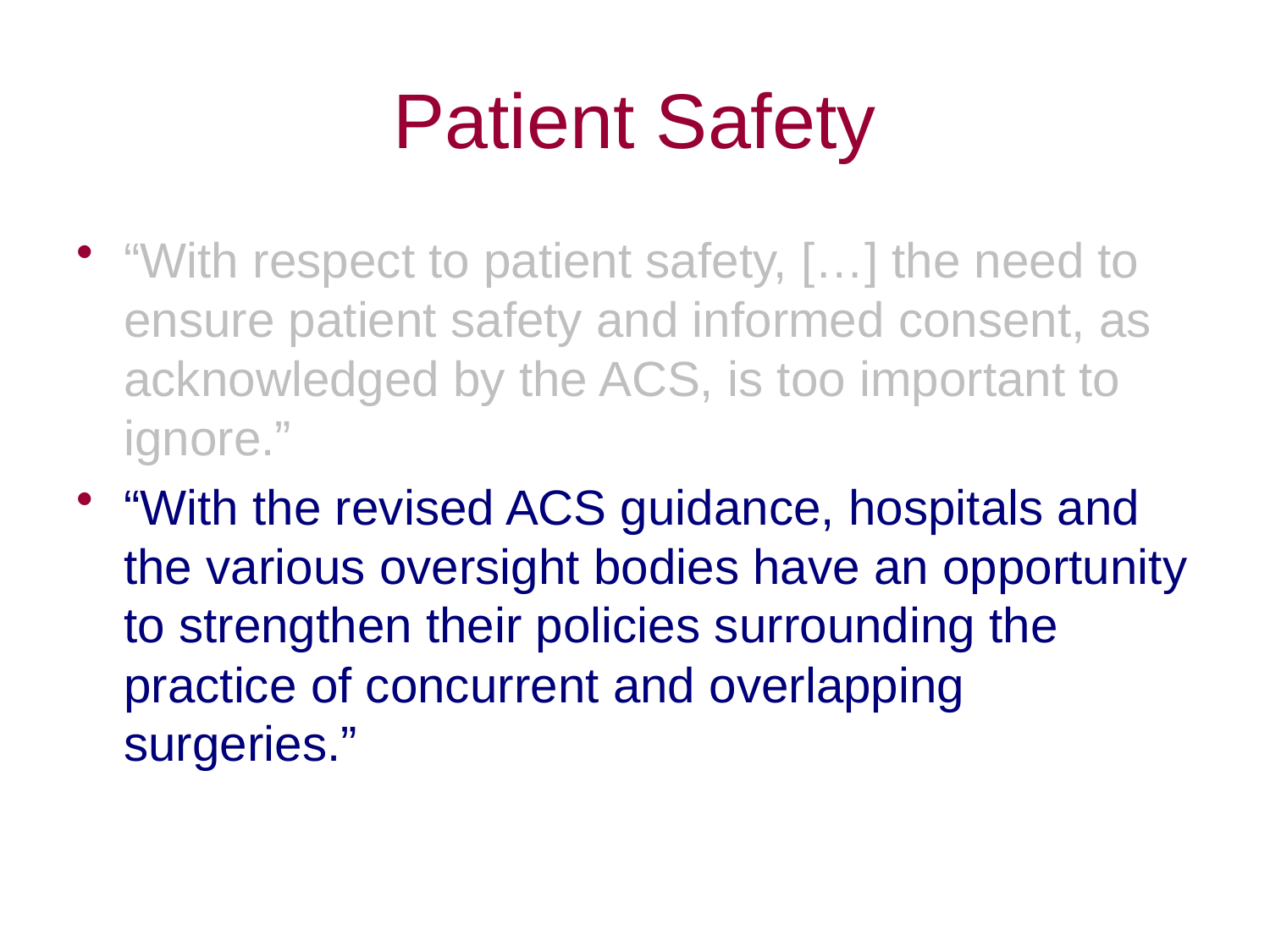

# Patient Safety
“With respect to patient safety, […] the need to ensure patient safety and informed consent, as acknowledged by the ACS, is too important to ignore.”
“With the revised ACS guidance, hospitals and the various oversight bodies have an opportunity to strengthen their policies surrounding the practice of concurrent and overlapping surgeries.”

## Slide 49
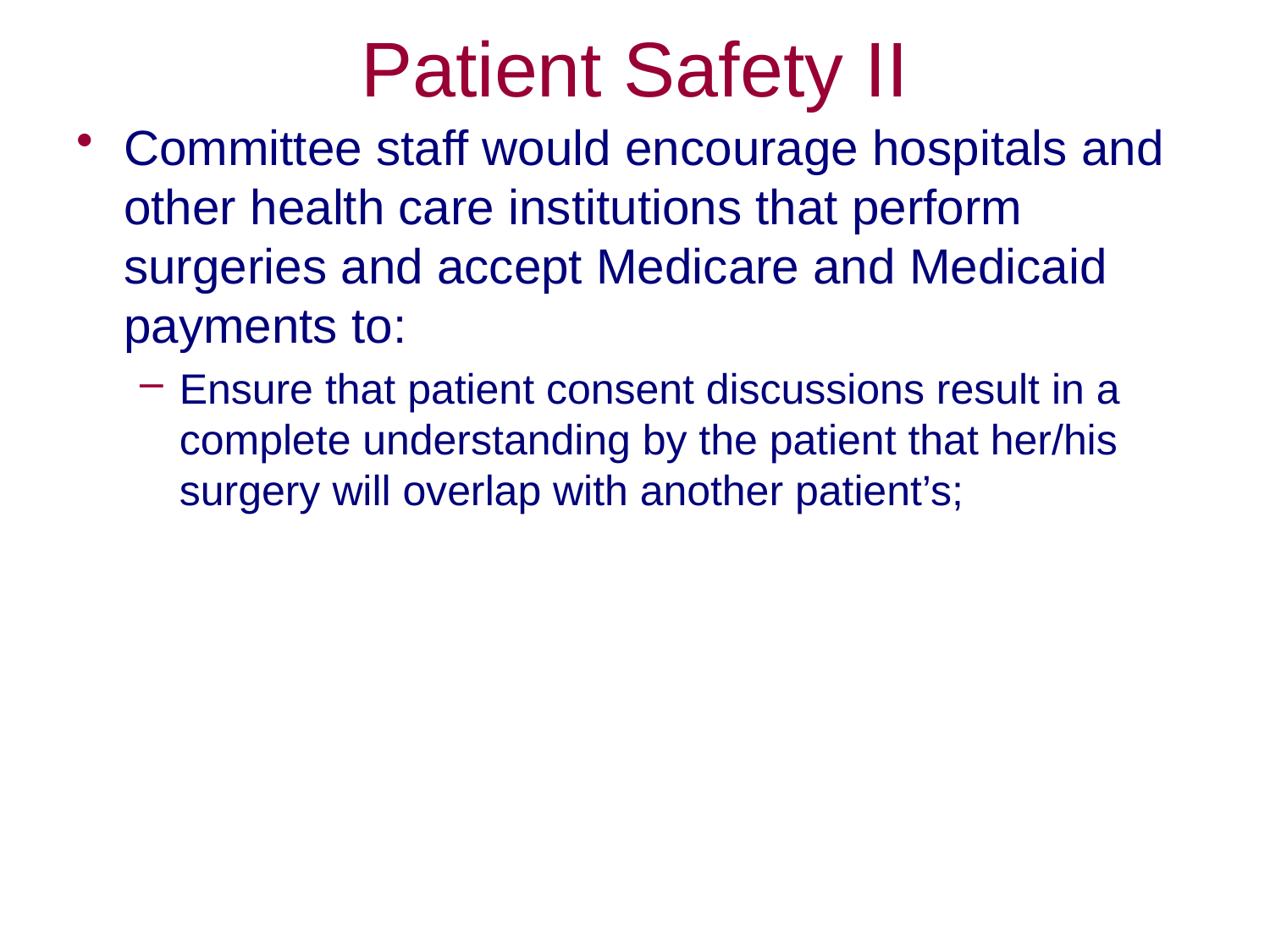

# Patient Safety II
Committee staff would encourage hospitals and other health care institutions that perform surgeries and accept Medicare and Medicaid payments to:
Ensure that patient consent discussions result in a complete understanding by the patient that her/his surgery will overlap with another patient’s;

## Slide 50
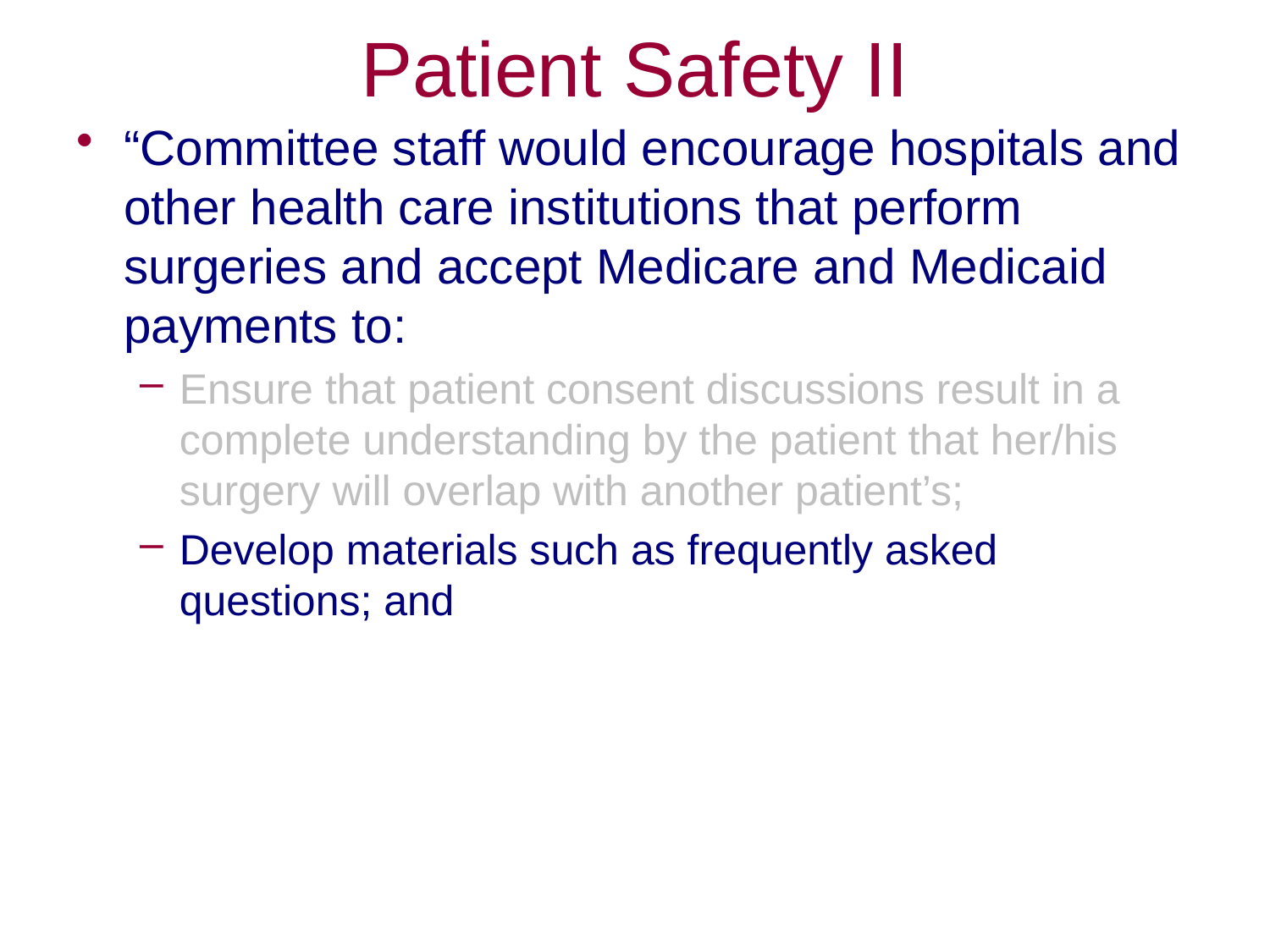

# Patient Safety II
“Committee staff would encourage hospitals and other health care institutions that perform surgeries and accept Medicare and Medicaid payments to:
Ensure that patient consent discussions result in a complete understanding by the patient that her/his surgery will overlap with another patient’s;
Develop materials such as frequently asked questions; and

## Slide 51
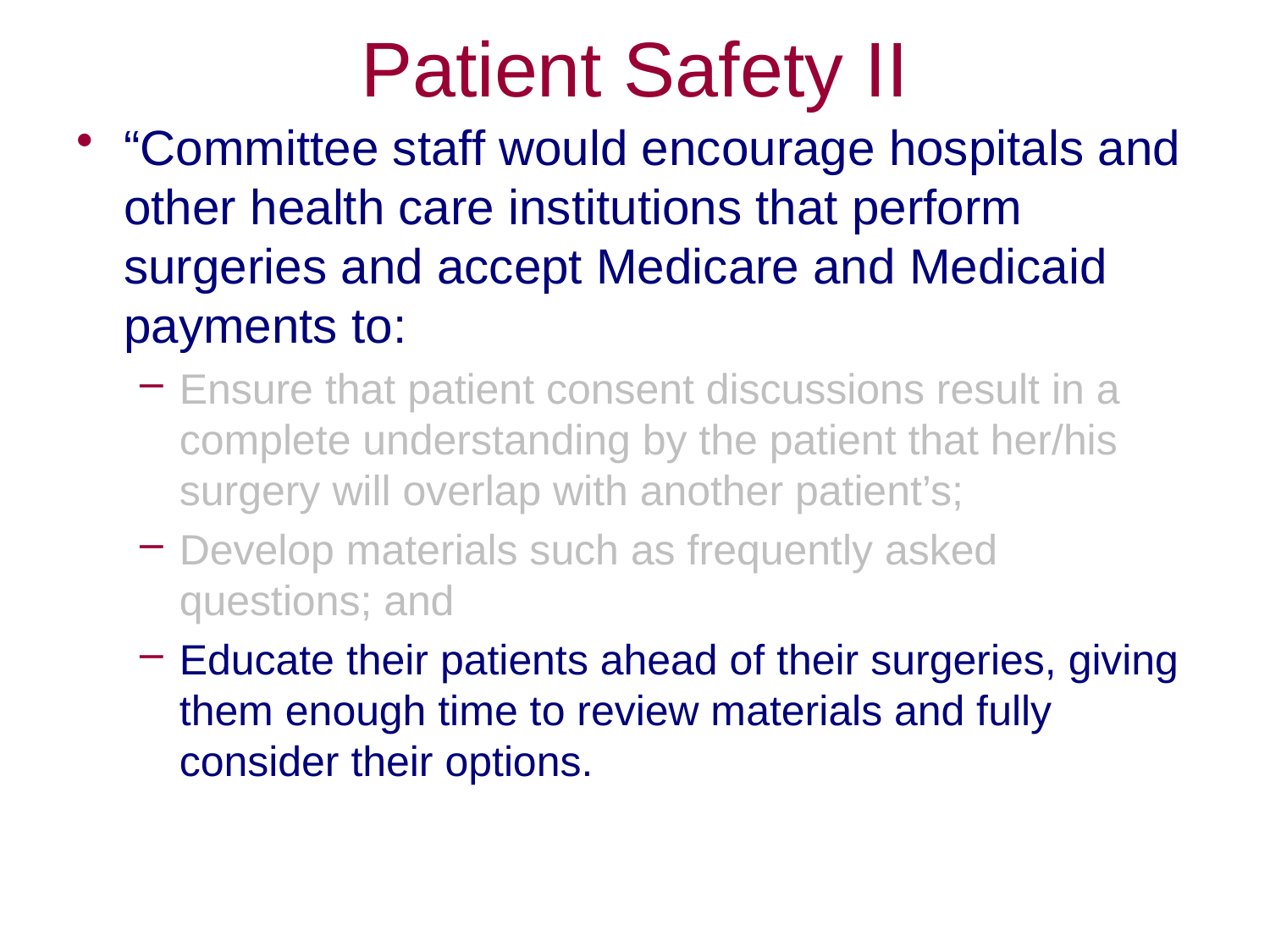

# Patient Safety II
“Committee staff would encourage hospitals and other health care institutions that perform surgeries and accept Medicare and Medicaid payments to:
Ensure that patient consent discussions result in a complete understanding by the patient that her/his surgery will overlap with another patient’s;
Develop materials such as frequently asked questions; and
Educate their patients ahead of their surgeries, giving them enough time to review materials and fully consider their options.

## Slide 52
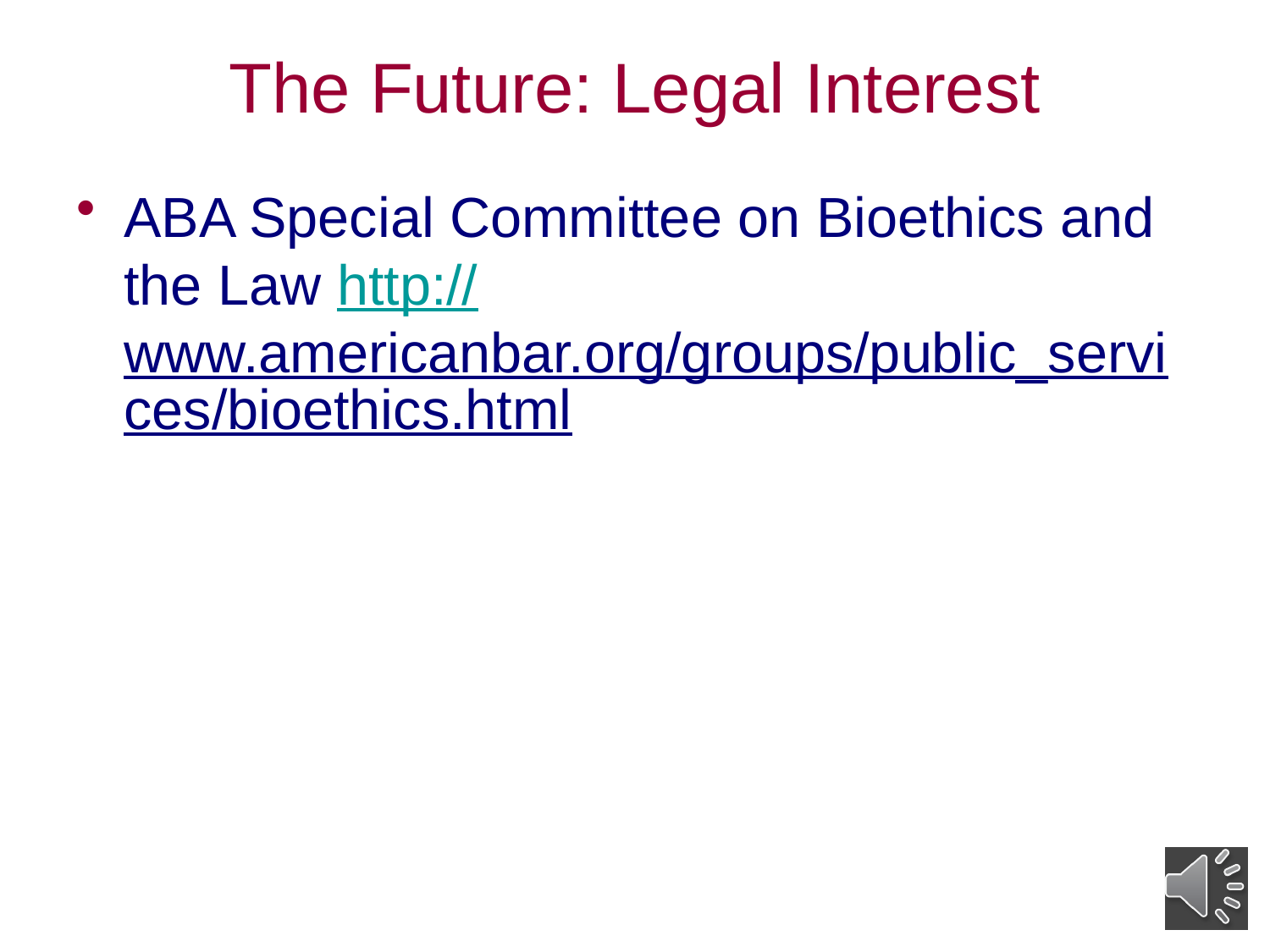

# The Future: Legal Interest
ABA Special Committee on Bioethics and the Law http://www.americanbar.org/groups/public_services/bioethics.html

## Slide 53
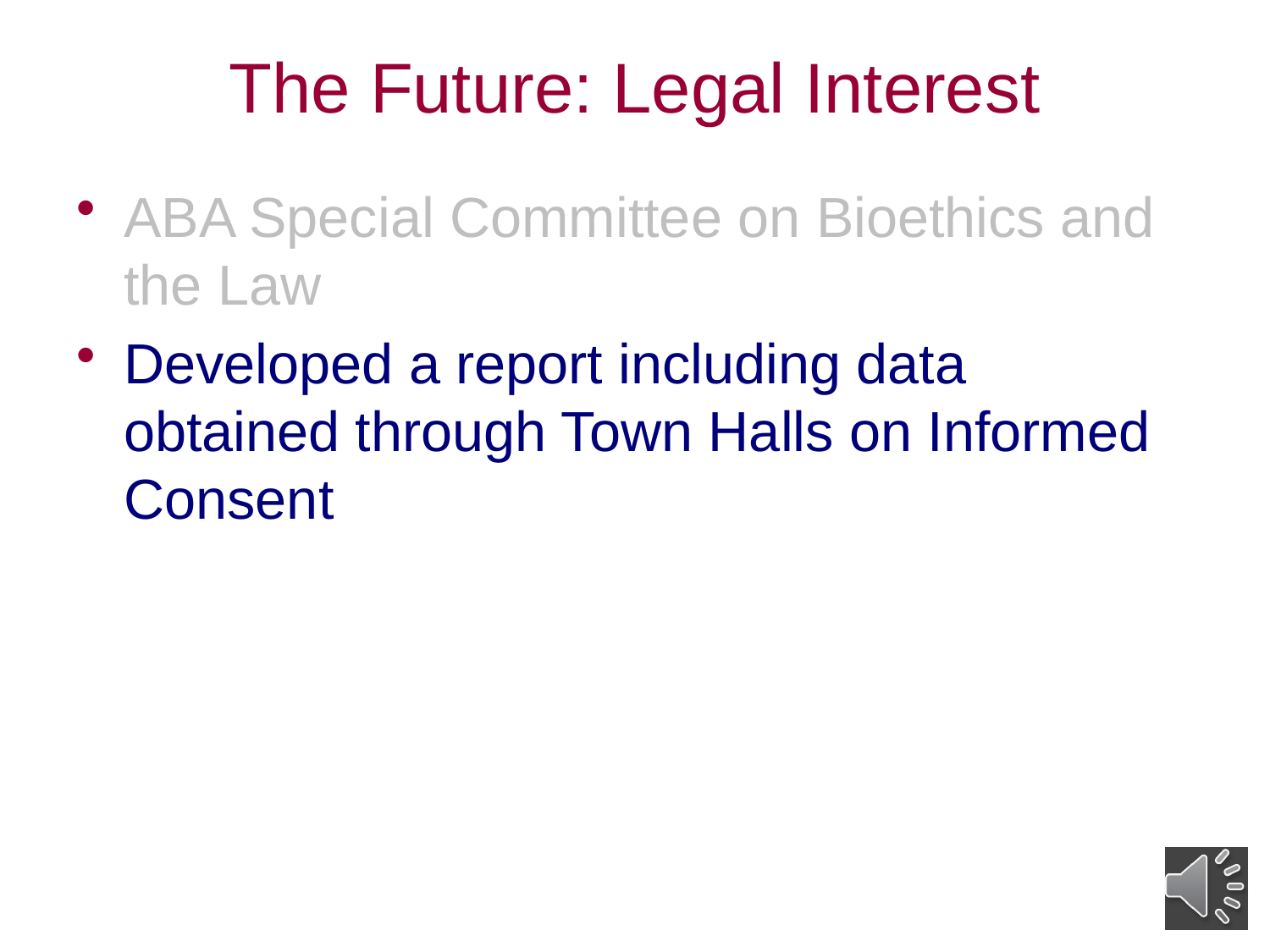

# The Future: Legal Interest
ABA Special Committee on Bioethics and the Law
Developed a report including data obtained through Town Halls on Informed Consent

## Slide 54
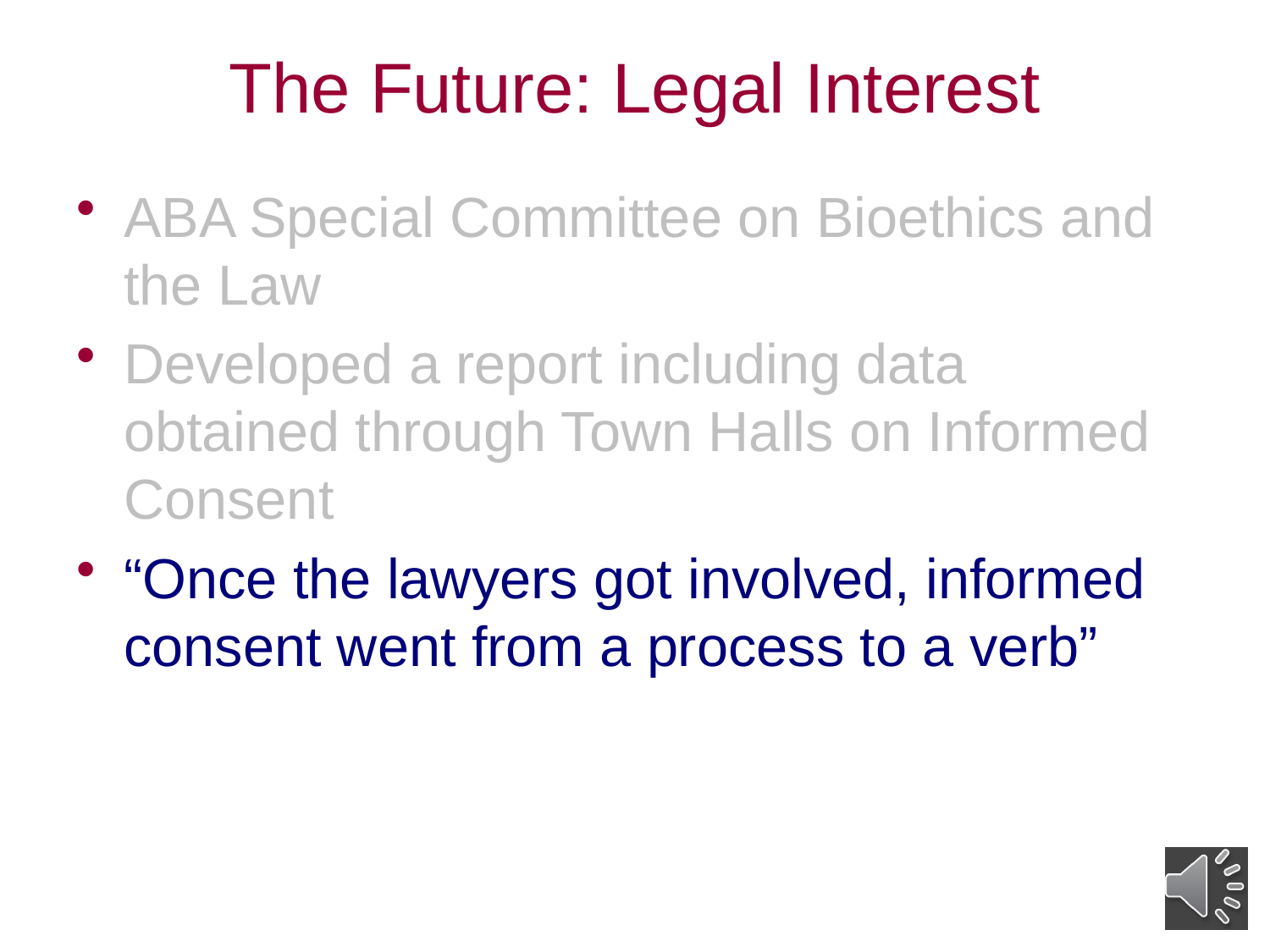

# The Future: Legal Interest
ABA Special Committee on Bioethics and the Law
Developed a report including data obtained through Town Halls on Informed Consent
“Once the lawyers got involved, informed consent went from a process to a verb”

## Slide 55
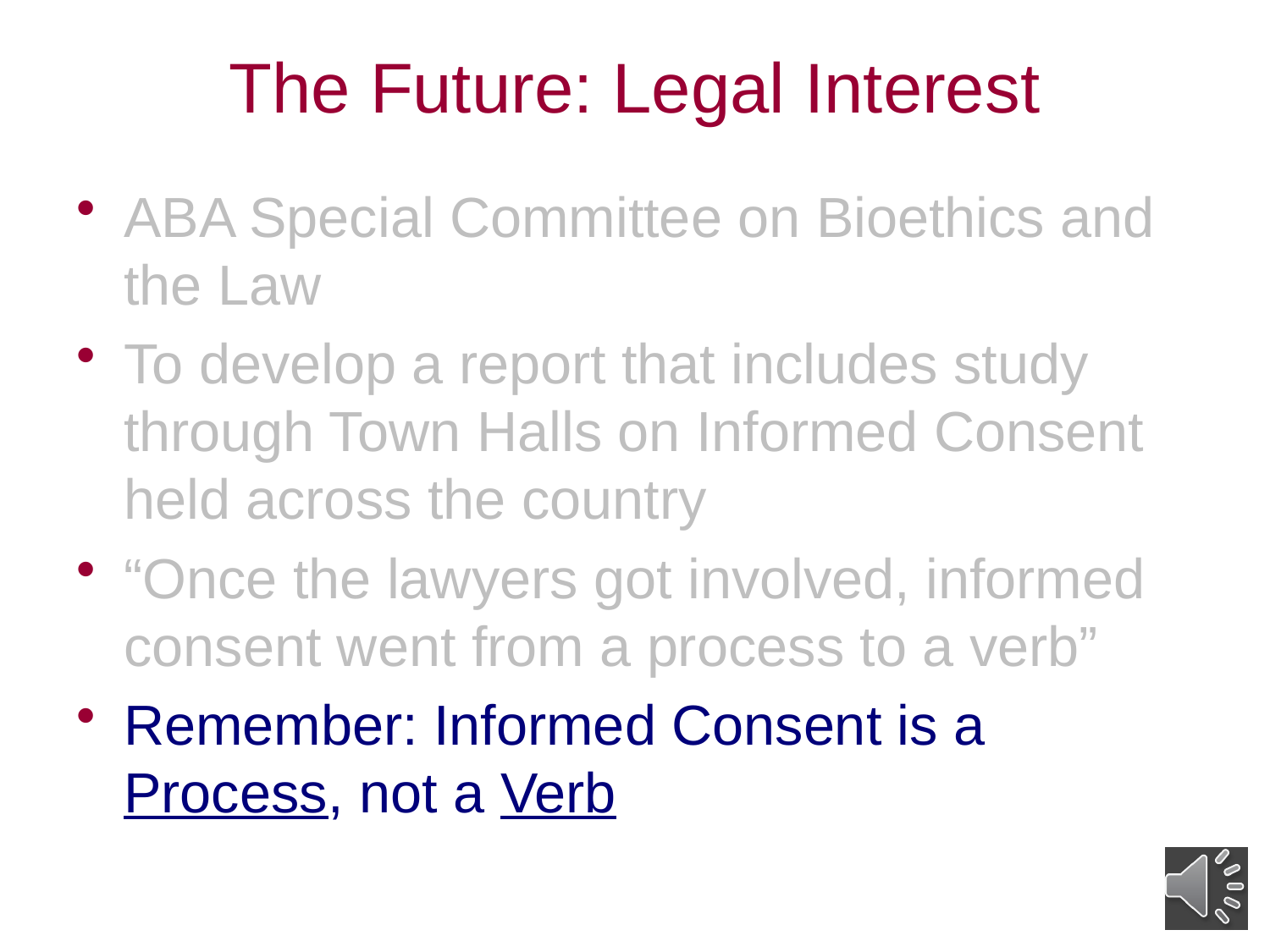

# The Future: Legal Interest
ABA Special Committee on Bioethics and the Law
To develop a report that includes study through Town Halls on Informed Consent held across the country
“Once the lawyers got involved, informed consent went from a process to a verb”
Remember: Informed Consent is a Process, not a Verb

## Slide 56
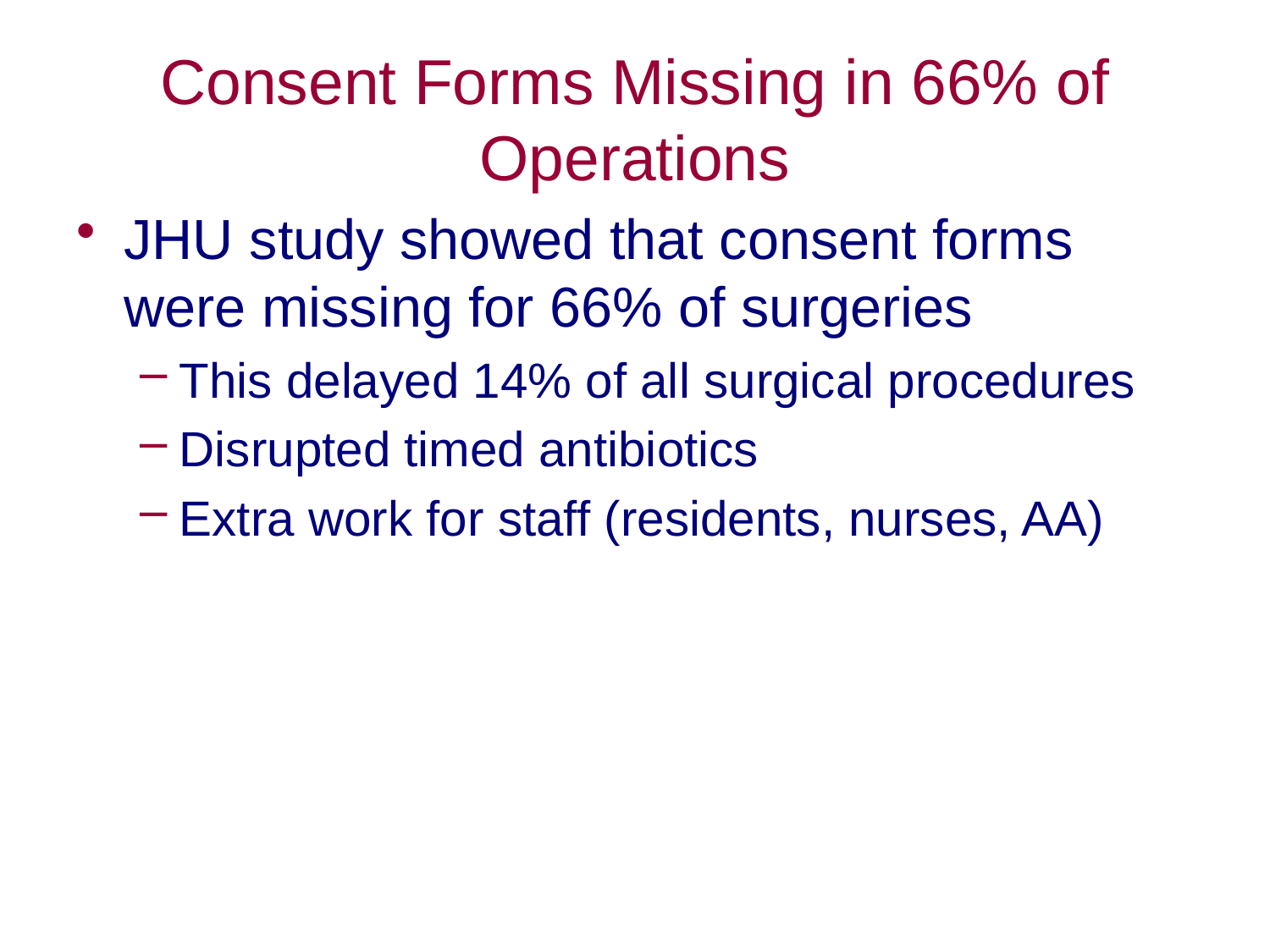

# Consent Forms Missing in 66% of Operations
JHU study showed that consent forms were missing for 66% of surgeries
This delayed 14% of all surgical procedures
Disrupted timed antibiotics
Extra work for staff (residents, nurses, AA)

## Slide 57
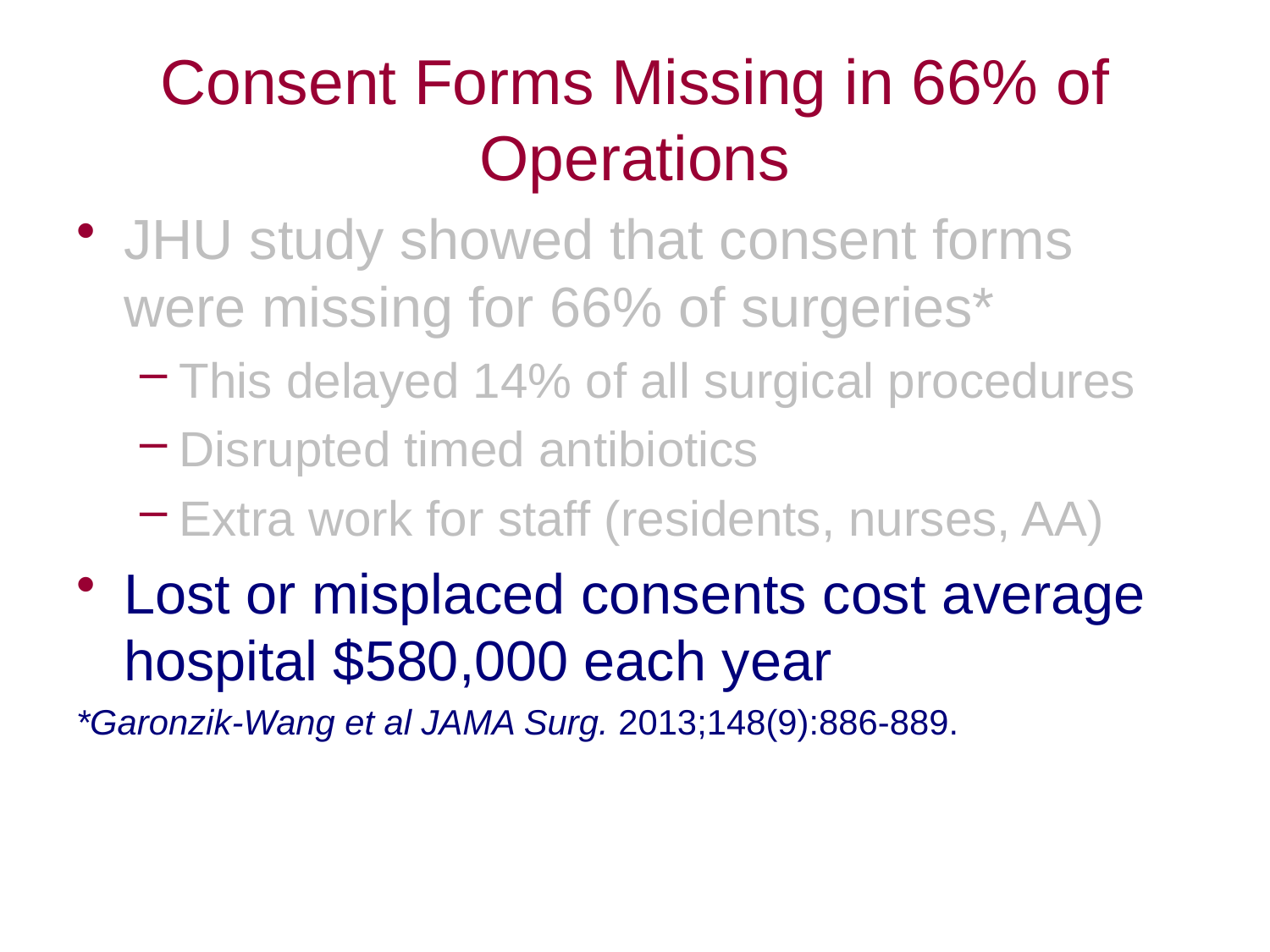

# Consent Forms Missing in 66% of Operations
JHU study showed that consent forms were missing for 66% of surgeries*
This delayed 14% of all surgical procedures
Disrupted timed antibiotics
Extra work for staff (residents, nurses, AA)
Lost or misplaced consents cost average hospital $580,000 each year
*Garonzik-Wang et al JAMA Surg. 2013;148(9):886-889.

## Slide 58
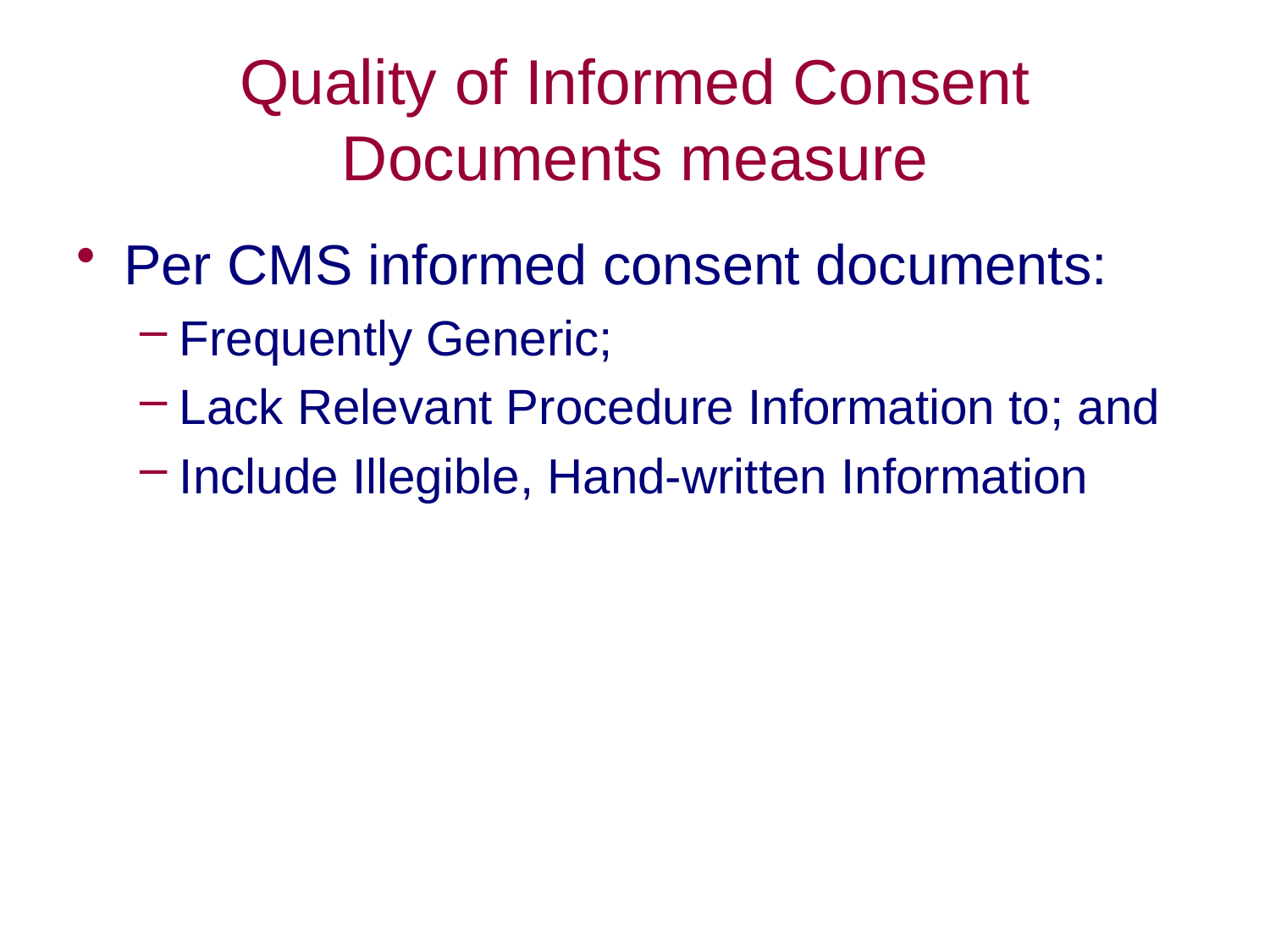

# Quality of Informed Consent Documents measure
Per CMS informed consent documents:
Frequently Generic;
Lack Relevant Procedure Information to; and
Include Illegible, Hand-written Information

## Slide 59
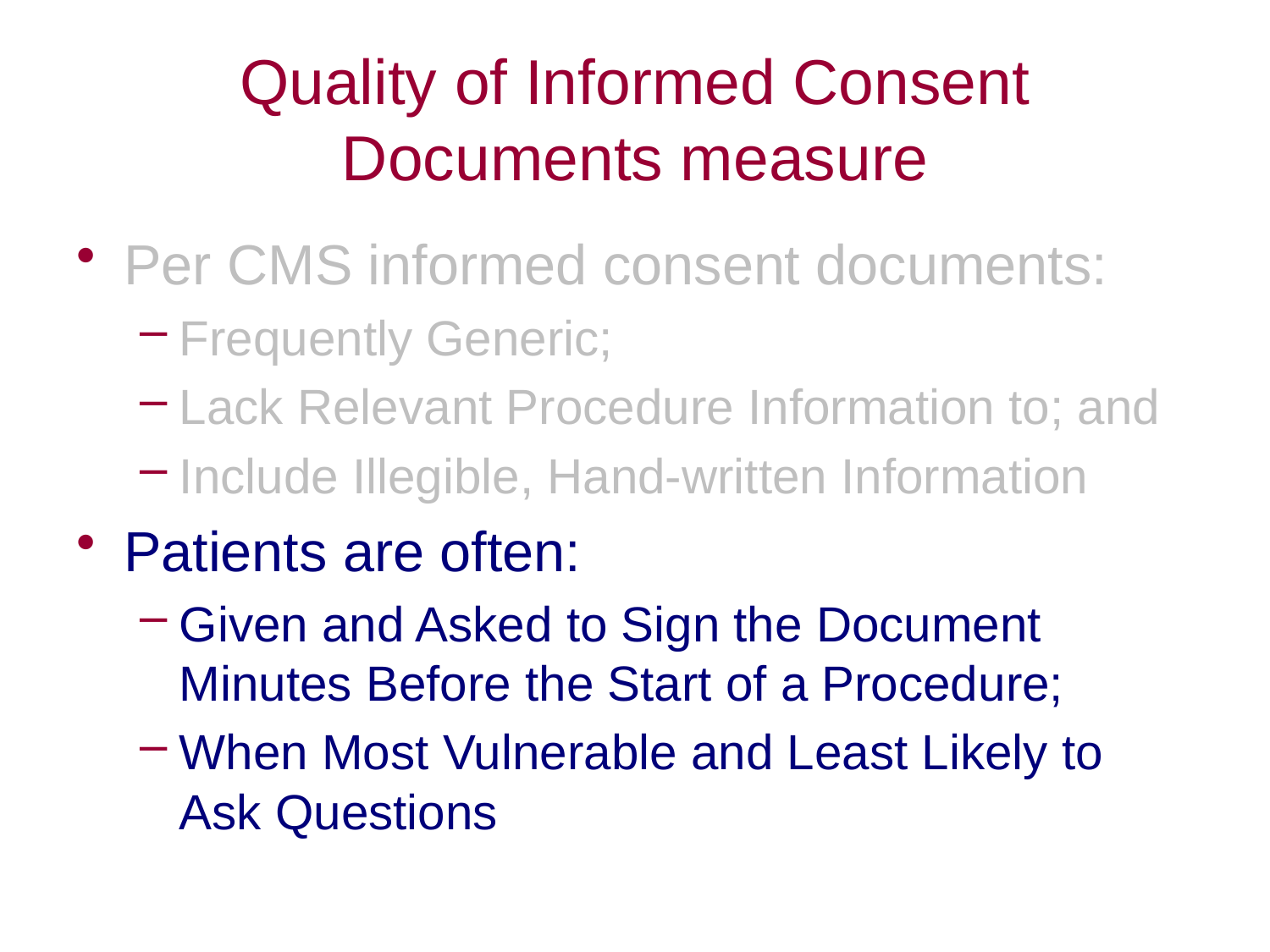

# Quality of Informed Consent Documents measure
Per CMS informed consent documents:
Frequently Generic;
Lack Relevant Procedure Information to; and
Include Illegible, Hand-written Information
Patients are often:
Given and Asked to Sign the Document Minutes Before the Start of a Procedure;
When Most Vulnerable and Least Likely to Ask Questions

## Slide 60
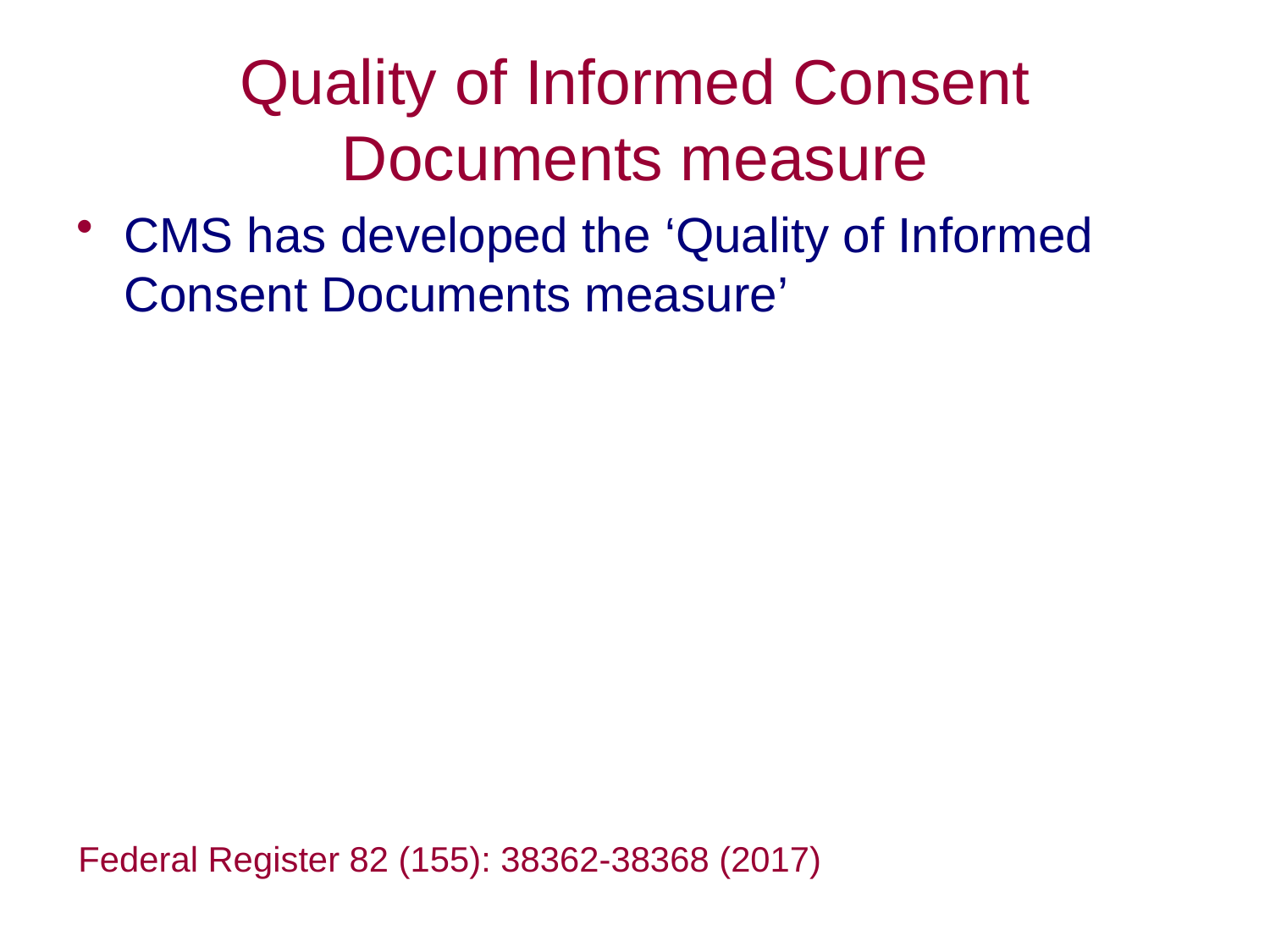

# Quality of Informed Consent Documents measure
CMS has developed the ‘Quality of Informed Consent Documents measure’
Federal Register 82 (155): 38362-38368 (2017)

## Slide 61
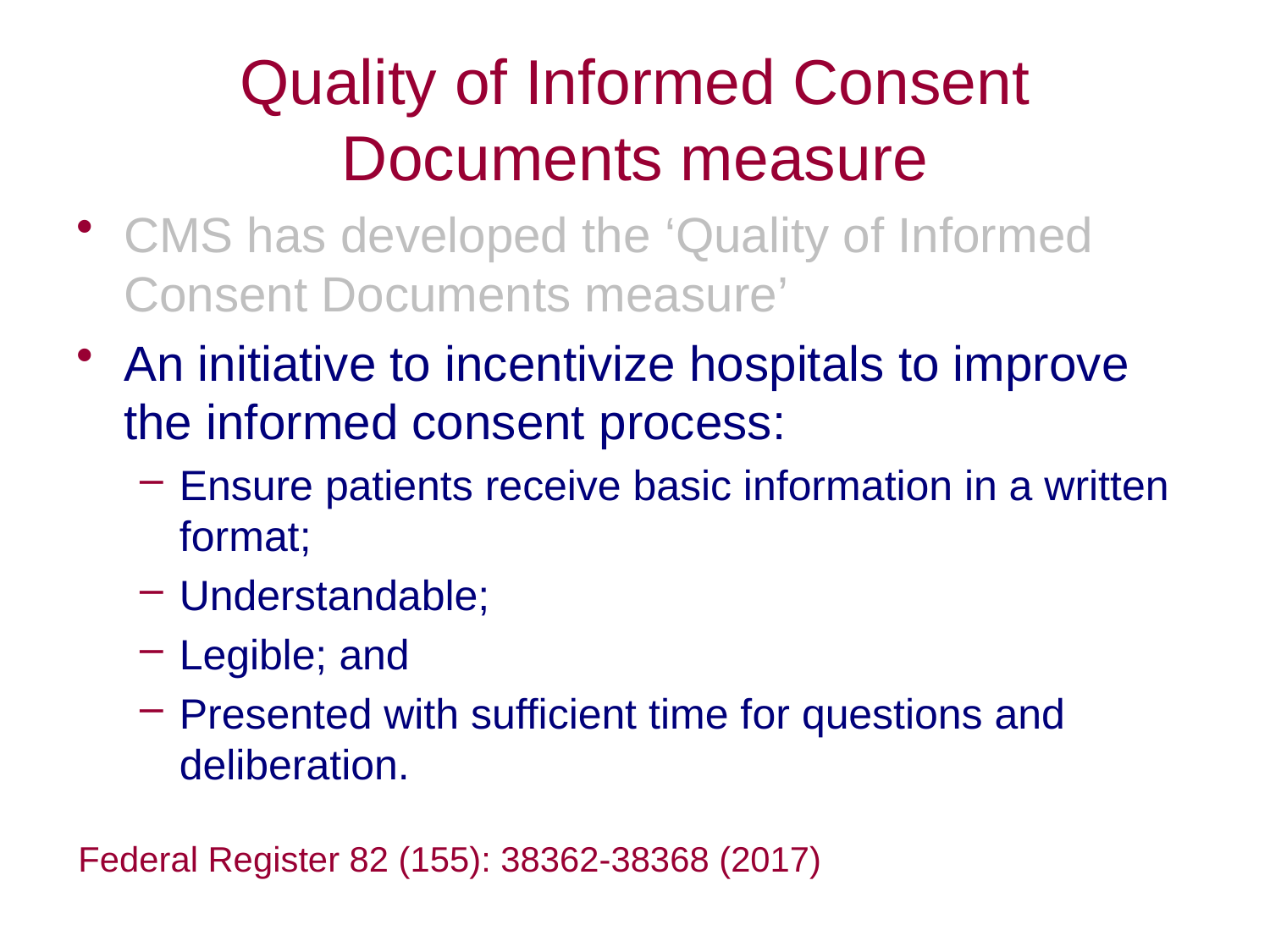

# Quality of Informed Consent Documents measure
CMS has developed the ‘Quality of Informed Consent Documents measure’
An initiative to incentivize hospitals to improve the informed consent process:
Ensure patients receive basic information in a written format;
Understandable;
Legible; and
Presented with sufficient time for questions and deliberation.
Federal Register 82 (155): 38362-38368 (2017)

## Slide 62
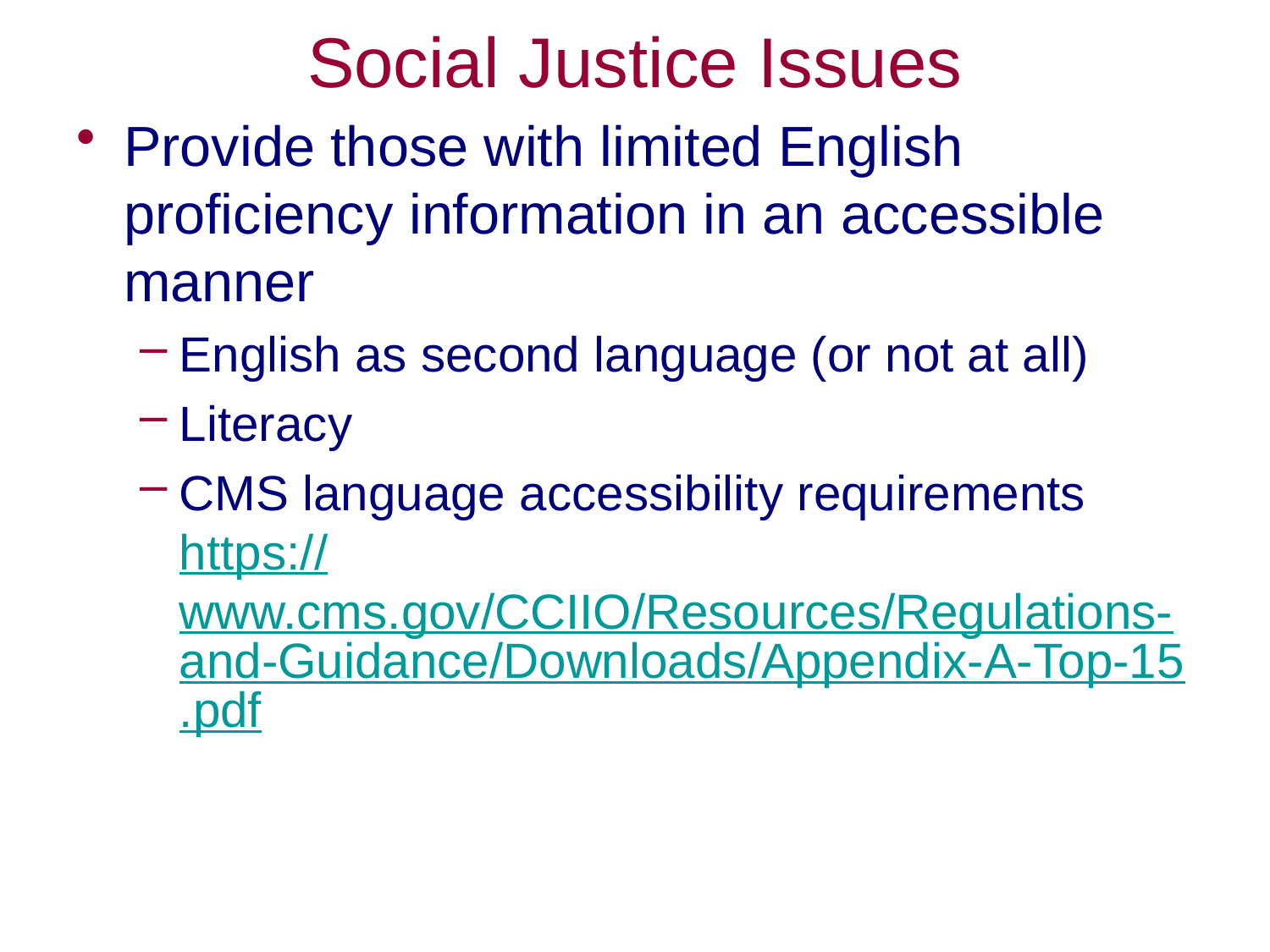

# Social Justice Issues
Provide those with limited English proficiency information in an accessible manner
English as second language (or not at all)
Literacy
CMS language accessibility requirements https://www.cms.gov/CCIIO/Resources/Regulations-and-Guidance/Downloads/Appendix-A-Top-15.pdf

## Slide 63
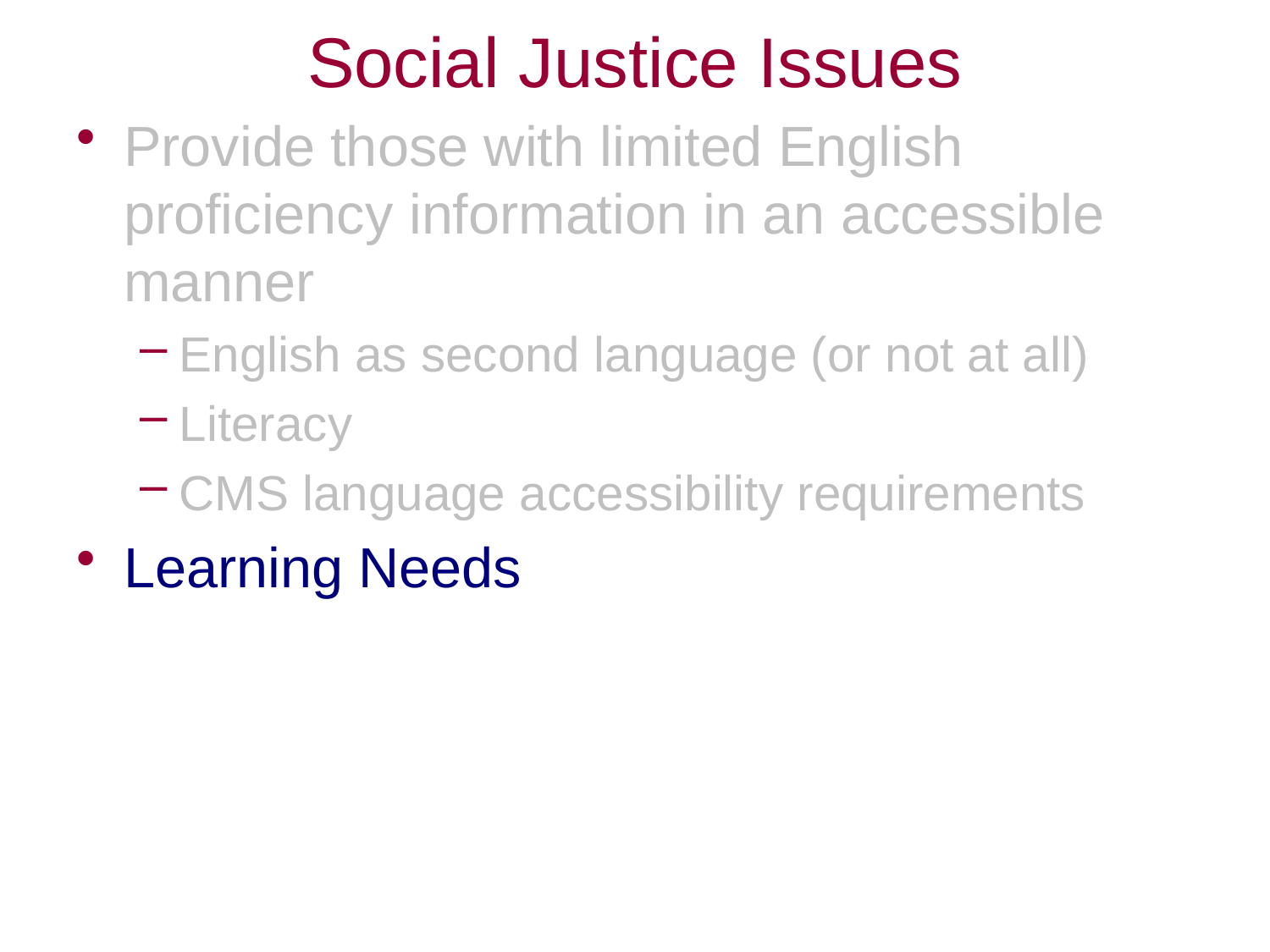

# Social Justice Issues
Provide those with limited English proficiency information in an accessible manner
English as second language (or not at all)
Literacy
CMS language accessibility requirements
Learning Needs

## Slide 64
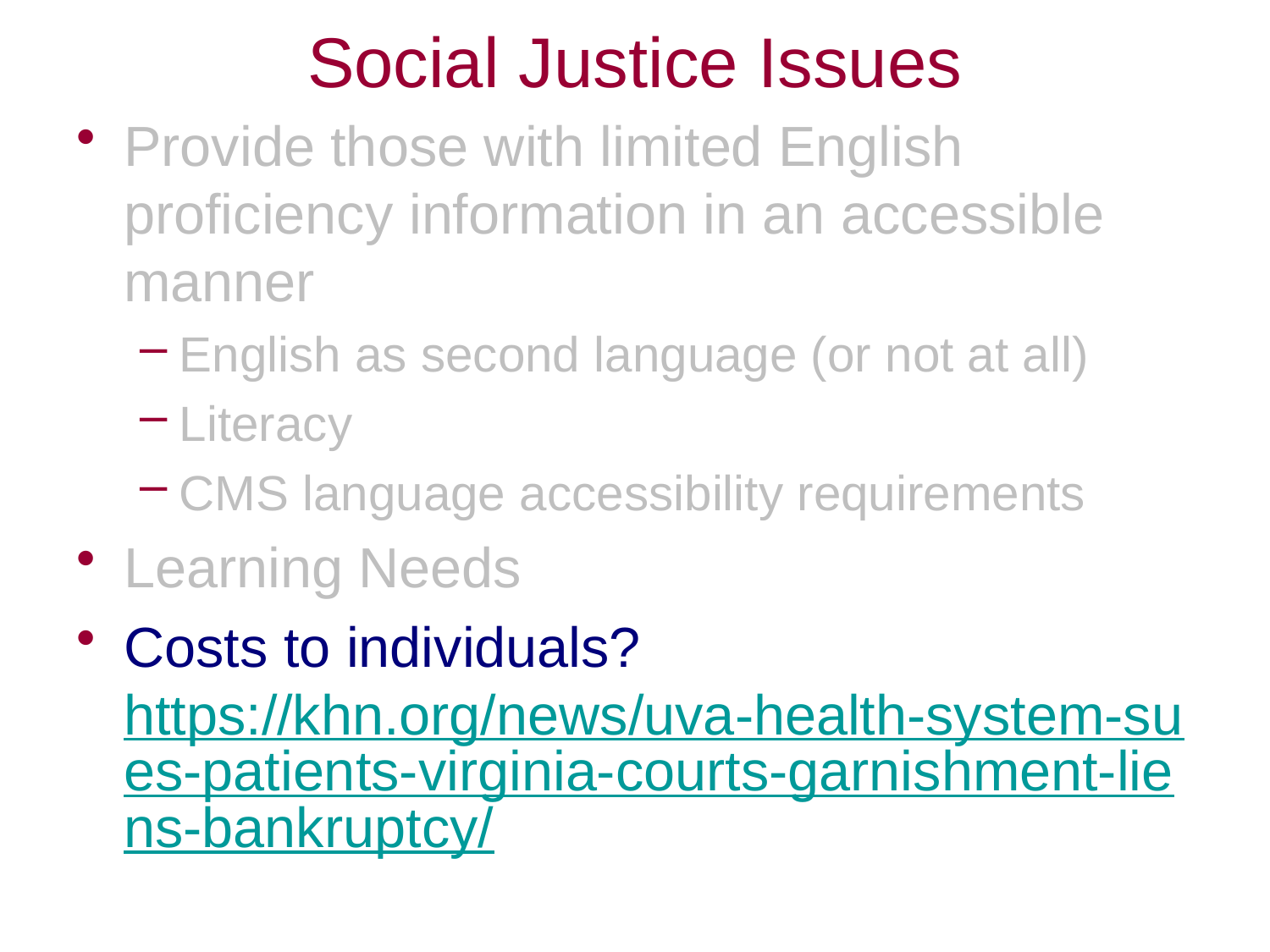

# Social Justice Issues
Provide those with limited English proficiency information in an accessible manner
English as second language (or not at all)
Literacy
CMS language accessibility requirements
Learning Needs
Costs to individuals? https://khn.org/news/uva-health-system-sues-patients-virginia-courts-garnishment-liens-bankruptcy/

## Slide 65
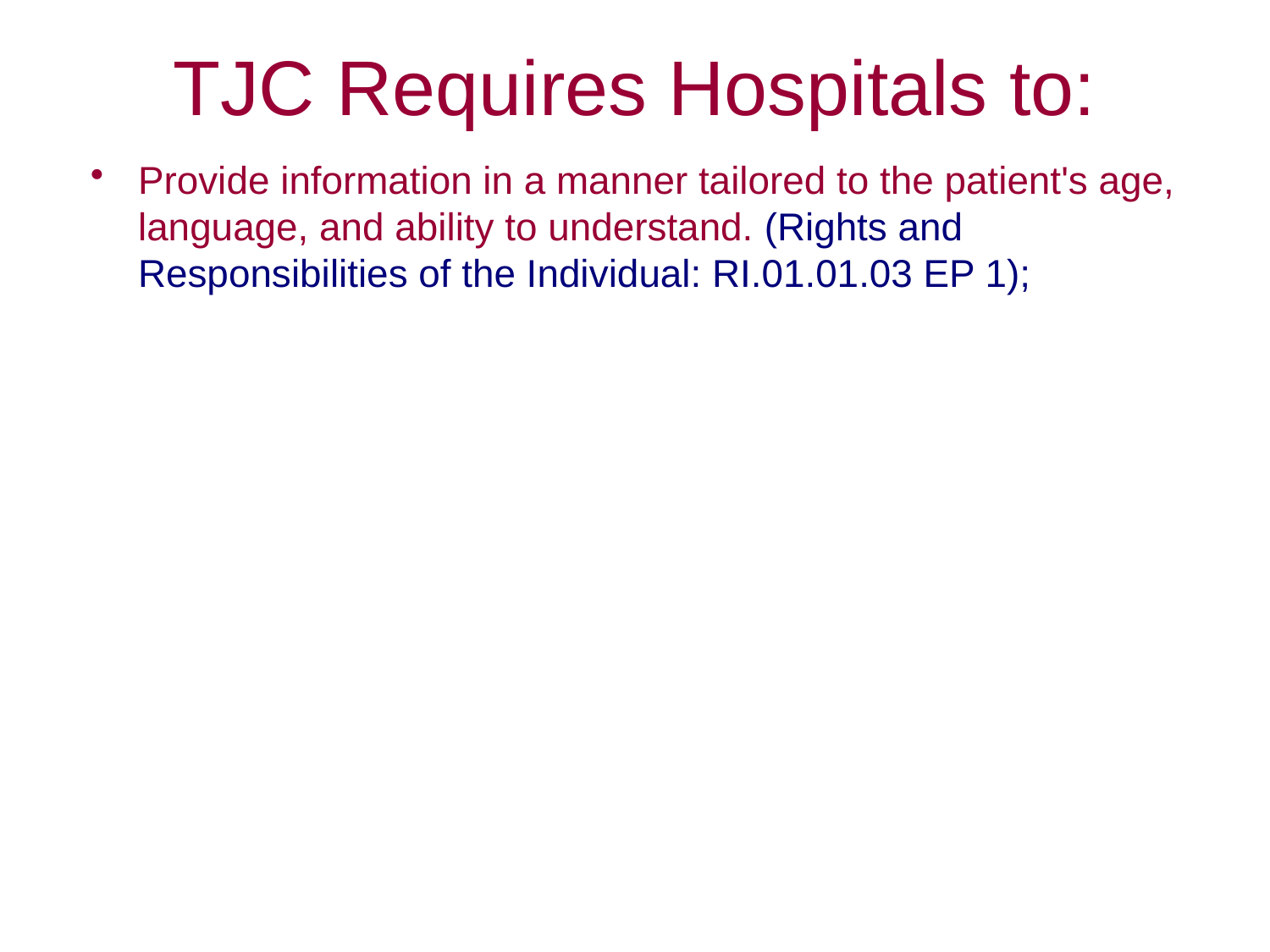

# TJC Requires Hospitals to:
Provide information in a manner tailored to the patient's age, language, and ability to understand. (Rights and Responsibilities of the Individual: RI.01.01.03 EP 1);

## Slide 66
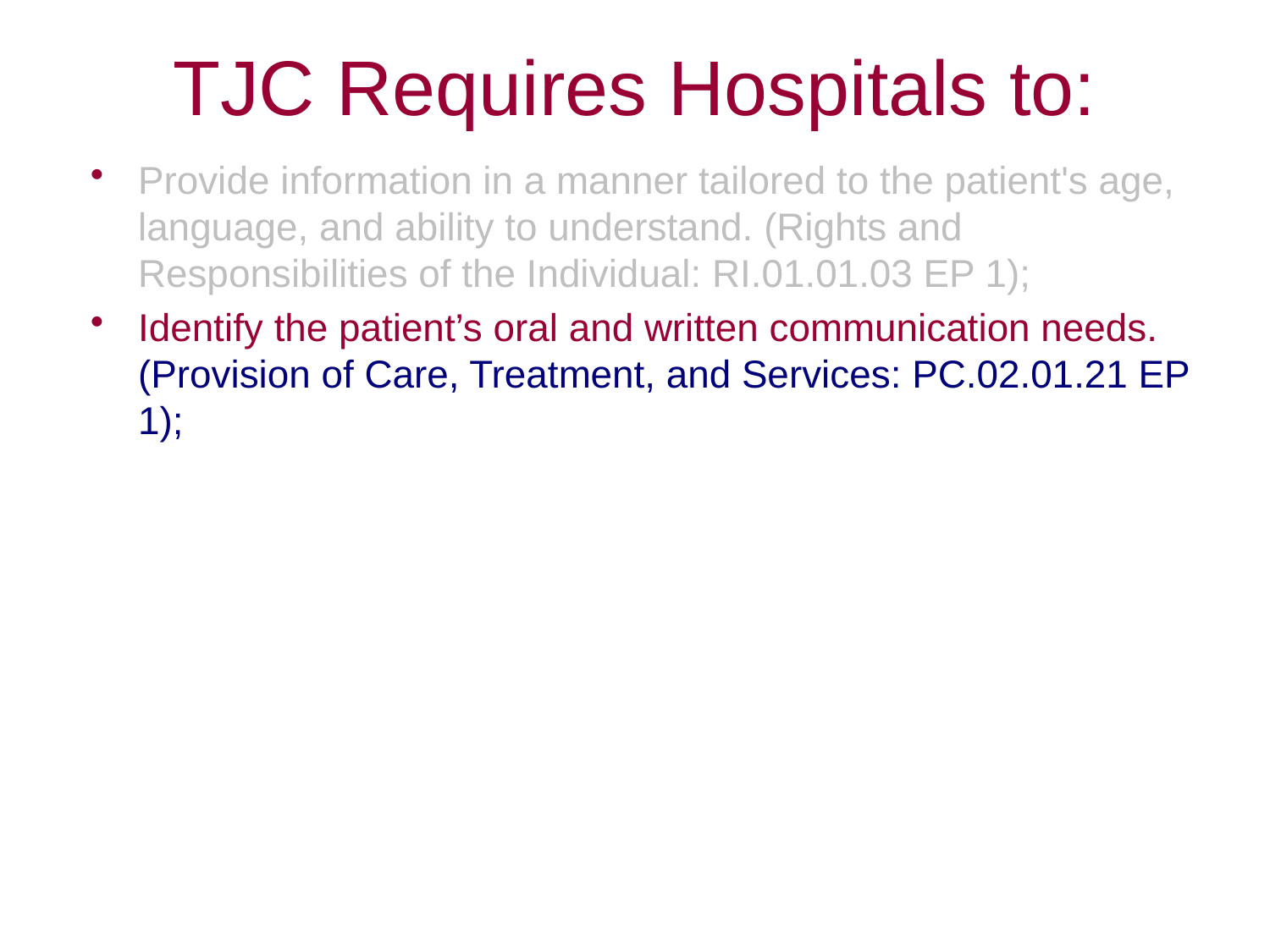

# TJC Requires Hospitals to:
Provide information in a manner tailored to the patient's age, language, and ability to understand. (Rights and Responsibilities of the Individual: RI.01.01.03 EP 1);
Identify the patient’s oral and written communication needs. (Provision of Care, Treatment, and Services: PC.02.01.21 EP 1);

## Slide 67
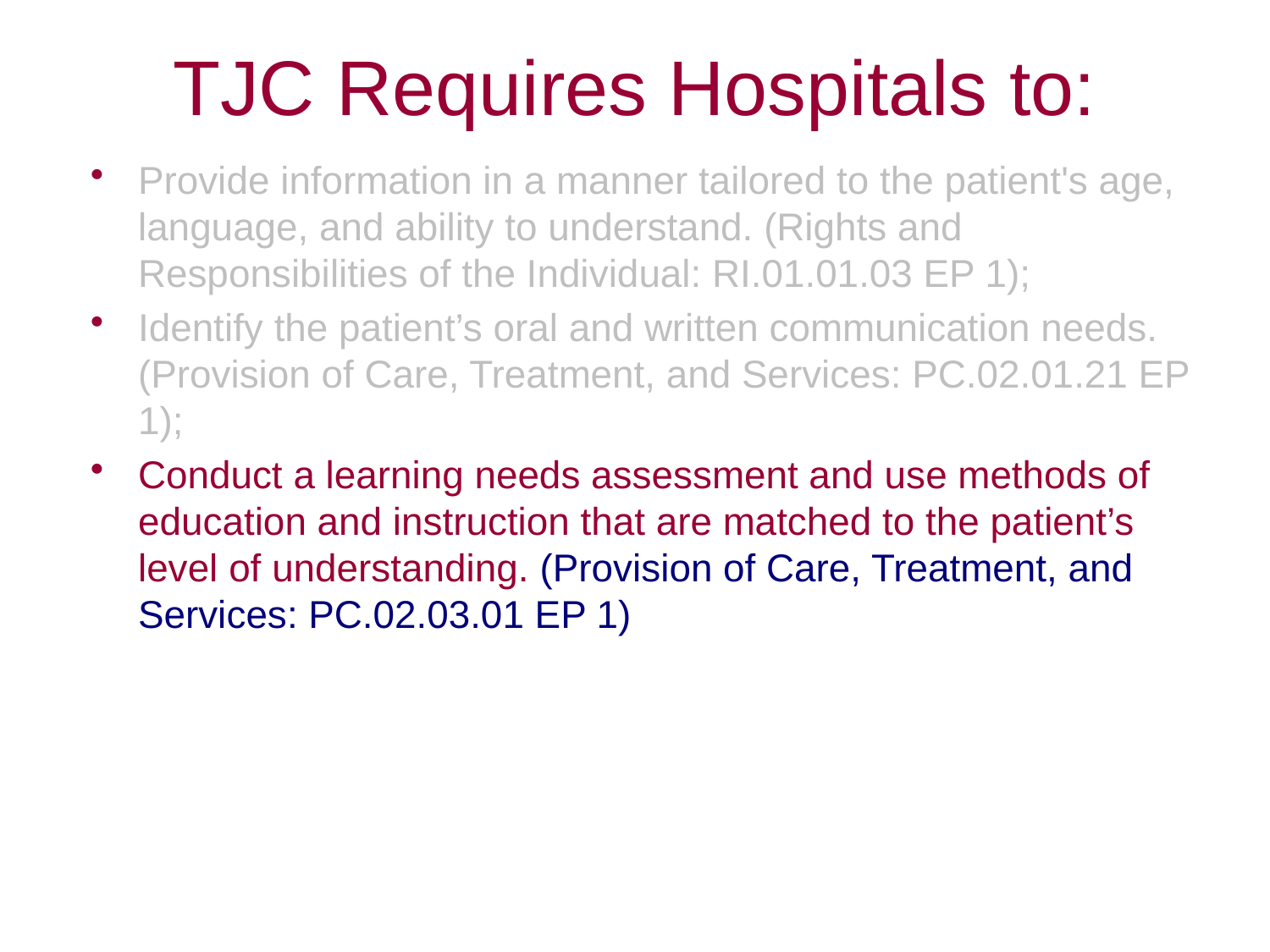

# TJC Requires Hospitals to:
Provide information in a manner tailored to the patient's age, language, and ability to understand. (Rights and Responsibilities of the Individual: RI.01.01.03 EP 1);
Identify the patient’s oral and written communication needs. (Provision of Care, Treatment, and Services: PC.02.01.21 EP 1);
Conduct a learning needs assessment and use methods of education and instruction that are matched to the patient’s level of understanding. (Provision of Care, Treatment, and Services: PC.02.03.01 EP 1)

## Slide 68
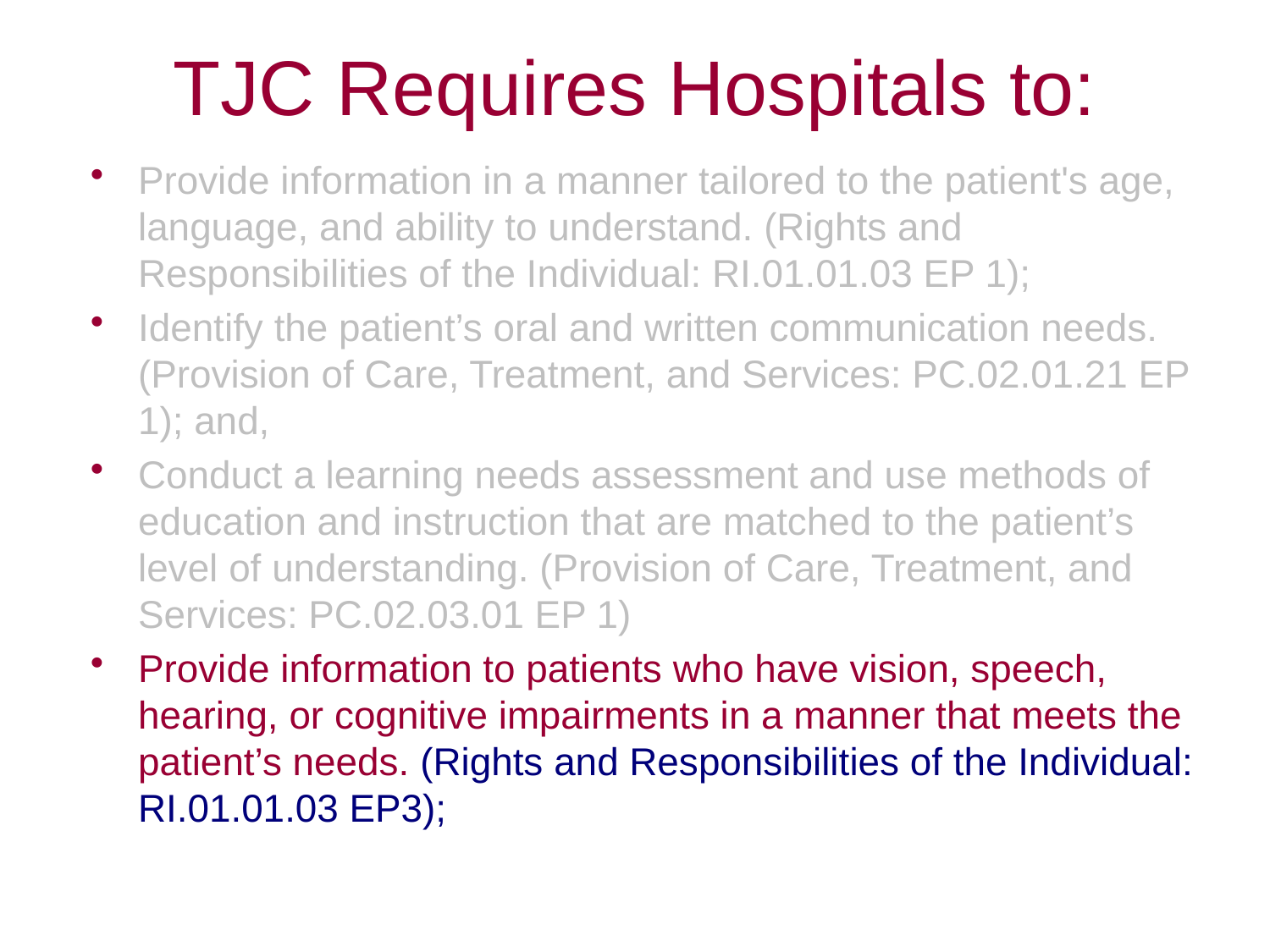

# TJC Requires Hospitals to:
Provide information in a manner tailored to the patient's age, language, and ability to understand. (Rights and Responsibilities of the Individual: RI.01.01.03 EP 1);
Identify the patient’s oral and written communication needs. (Provision of Care, Treatment, and Services: PC.02.01.21 EP 1); and,
Conduct a learning needs assessment and use methods of education and instruction that are matched to the patient’s level of understanding. (Provision of Care, Treatment, and Services: PC.02.03.01 EP 1)
Provide information to patients who have vision, speech, hearing, or cognitive impairments in a manner that meets the patient’s needs. (Rights and Responsibilities of the Individual: RI.01.01.03 EP3);

## Slide 69
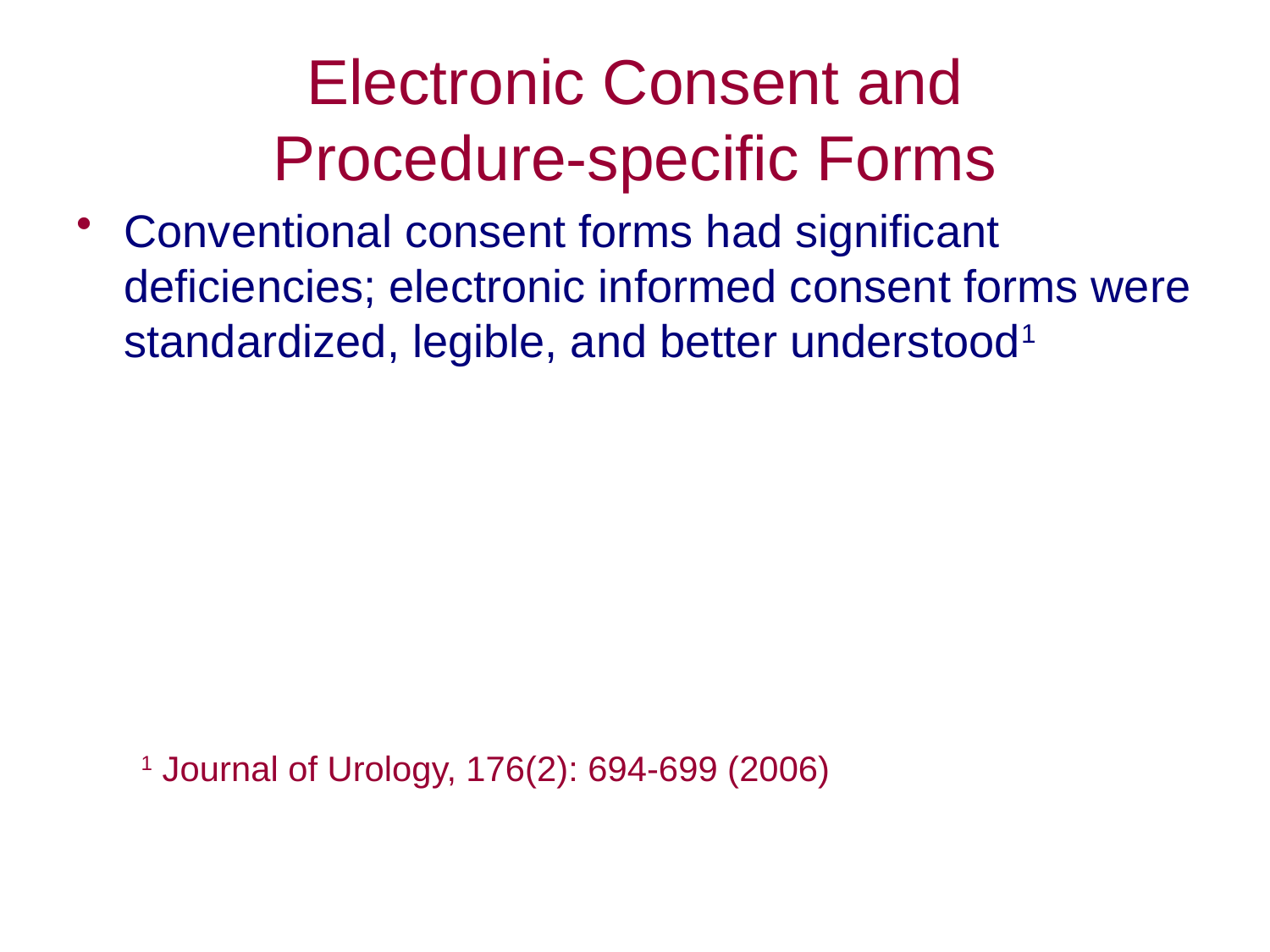

# Electronic Consent and Procedure-specific Forms
Conventional consent forms had significant deficiencies; electronic informed consent forms were standardized, legible, and better understood1
1 Journal of Urology, 176(2): 694-699 (2006)

## Slide 70
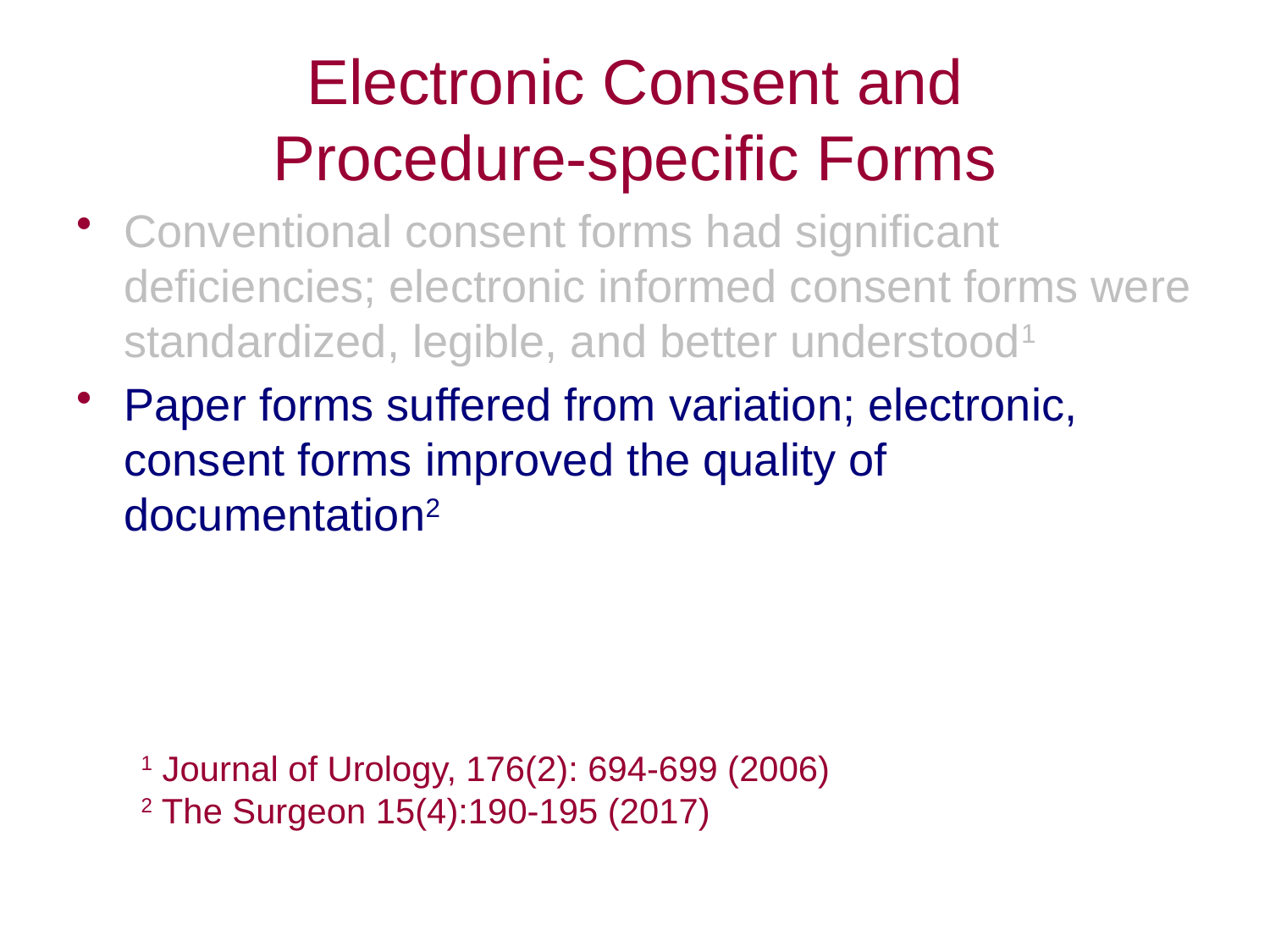

# Electronic Consent and Procedure-specific Forms
Conventional consent forms had significant deficiencies; electronic informed consent forms were standardized, legible, and better understood1
Paper forms suffered from variation; electronic, consent forms improved the quality of documentation2
1 Journal of Urology, 176(2): 694-699 (2006)
2 The Surgeon 15(4):190-195 (2017)

## Slide 71
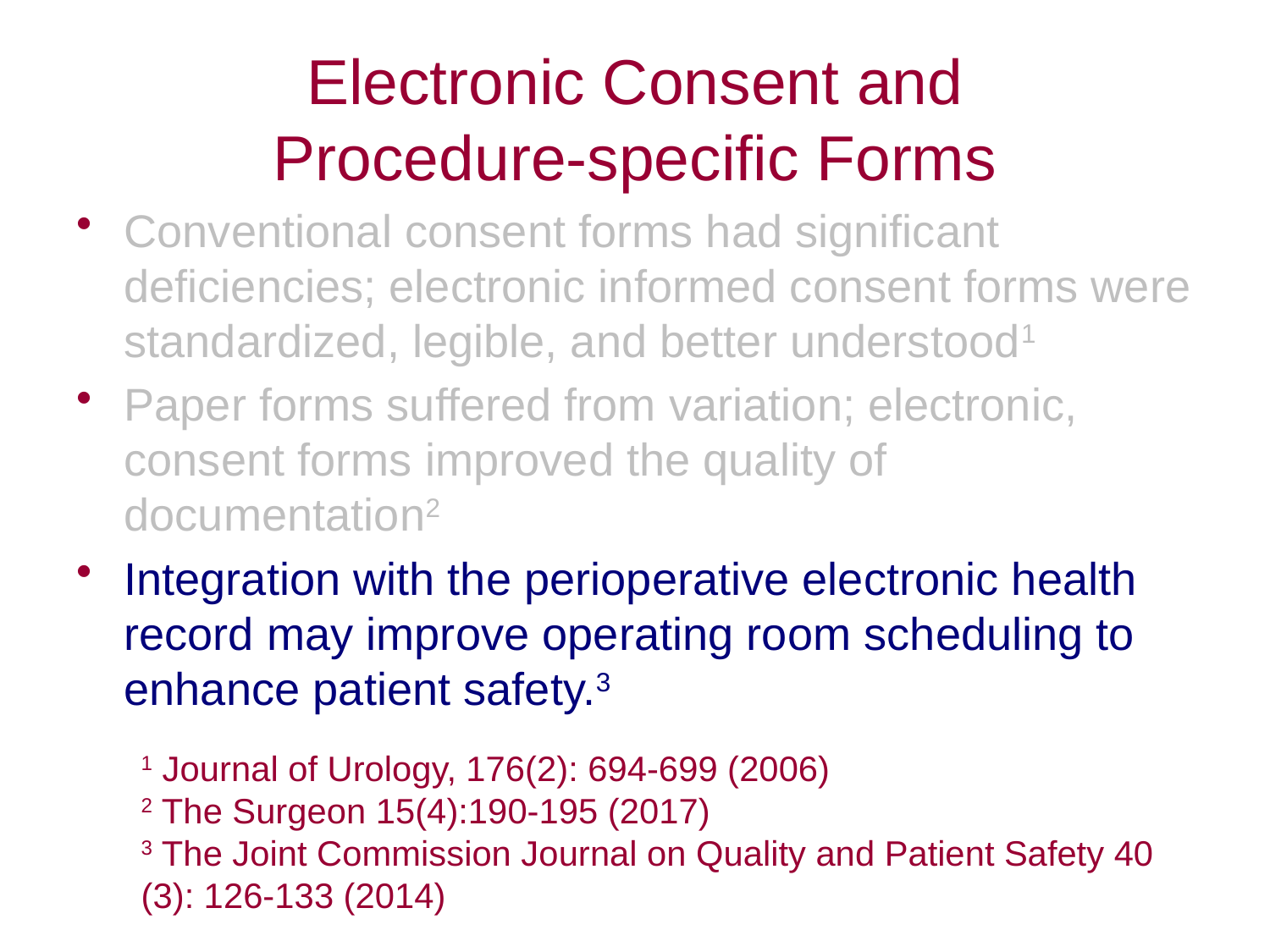

# Electronic Consent and Procedure-specific Forms
Conventional consent forms had significant deficiencies; electronic informed consent forms were standardized, legible, and better understood1
Paper forms suffered from variation; electronic, consent forms improved the quality of documentation2
Integration with the perioperative electronic health record may improve operating room scheduling to enhance patient safety.3
1 Journal of Urology, 176(2): 694-699 (2006)
2 The Surgeon 15(4):190-195 (2017)
3 The Joint Commission Journal on Quality and Patient Safety 40 (3): 126-133 (2014)

## Slide 72
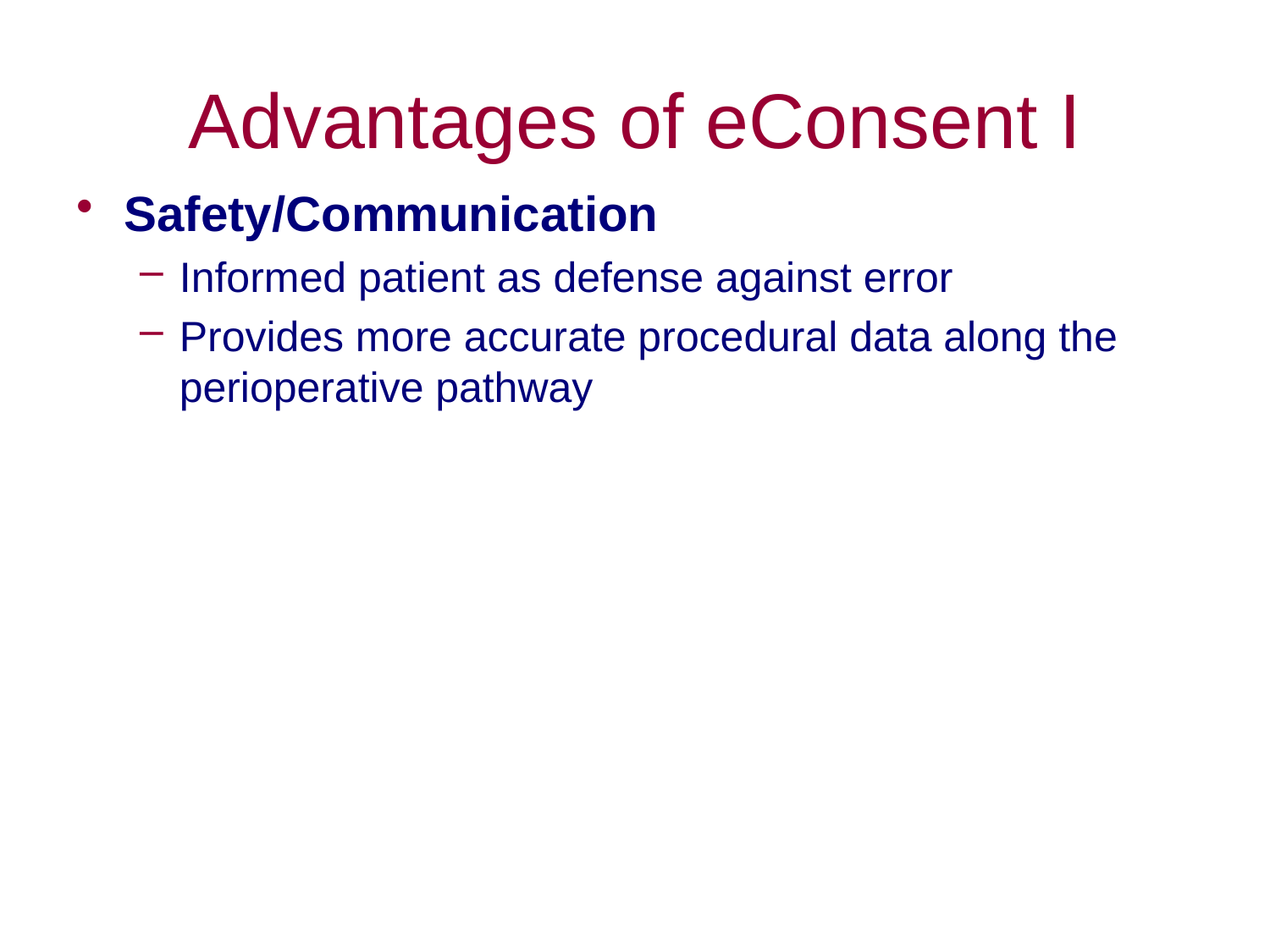

# Advantages of eConsent I
Safety/Communication
Informed patient as defense against error
Provides more accurate procedural data along the perioperative pathway

## Slide 73
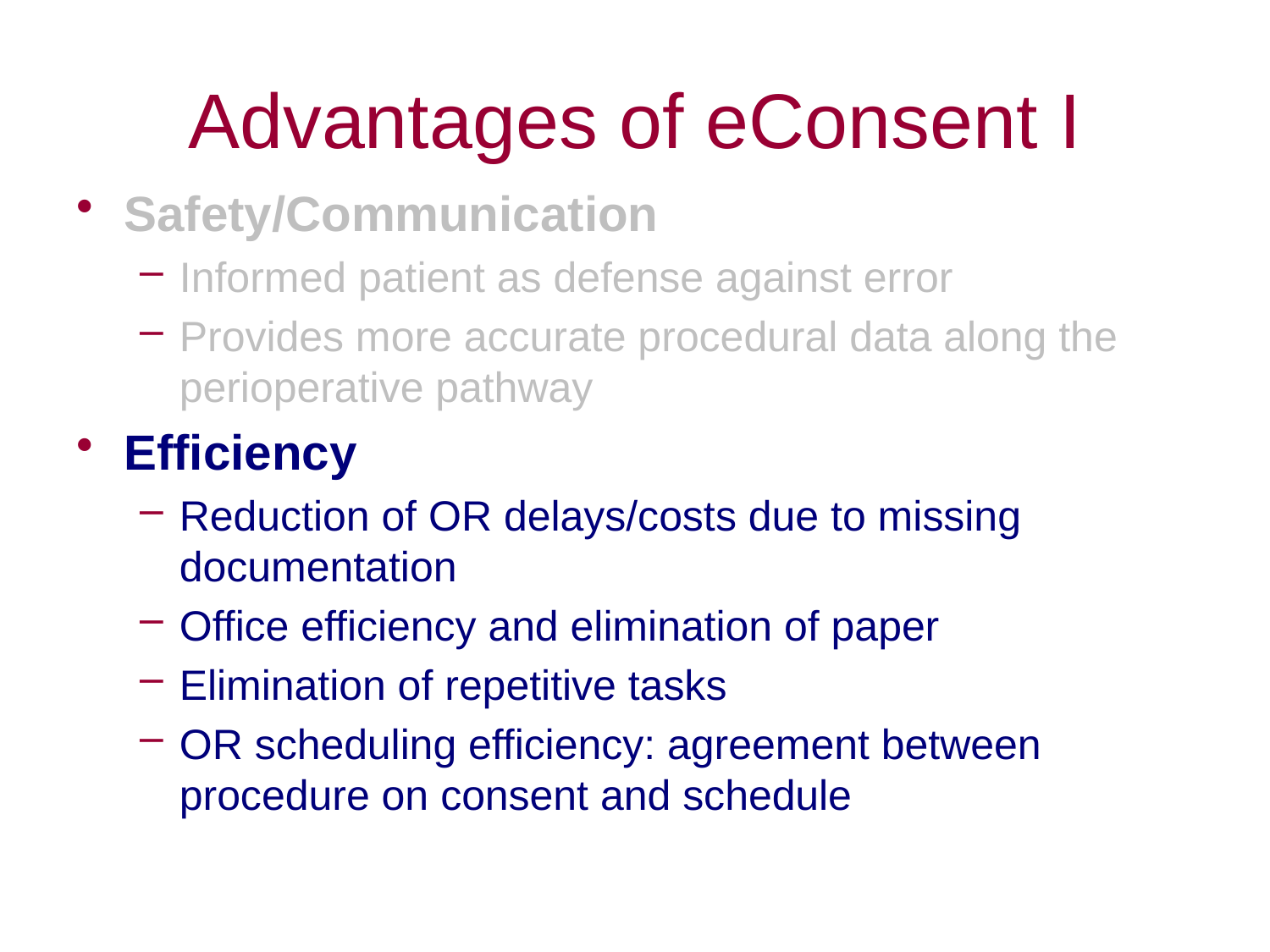

# Advantages of eConsent I
Safety/Communication
Informed patient as defense against error
Provides more accurate procedural data along the perioperative pathway
Efficiency
Reduction of OR delays/costs due to missing documentation
Office efficiency and elimination of paper
Elimination of repetitive tasks
OR scheduling efficiency: agreement between procedure on consent and schedule

## Slide 74
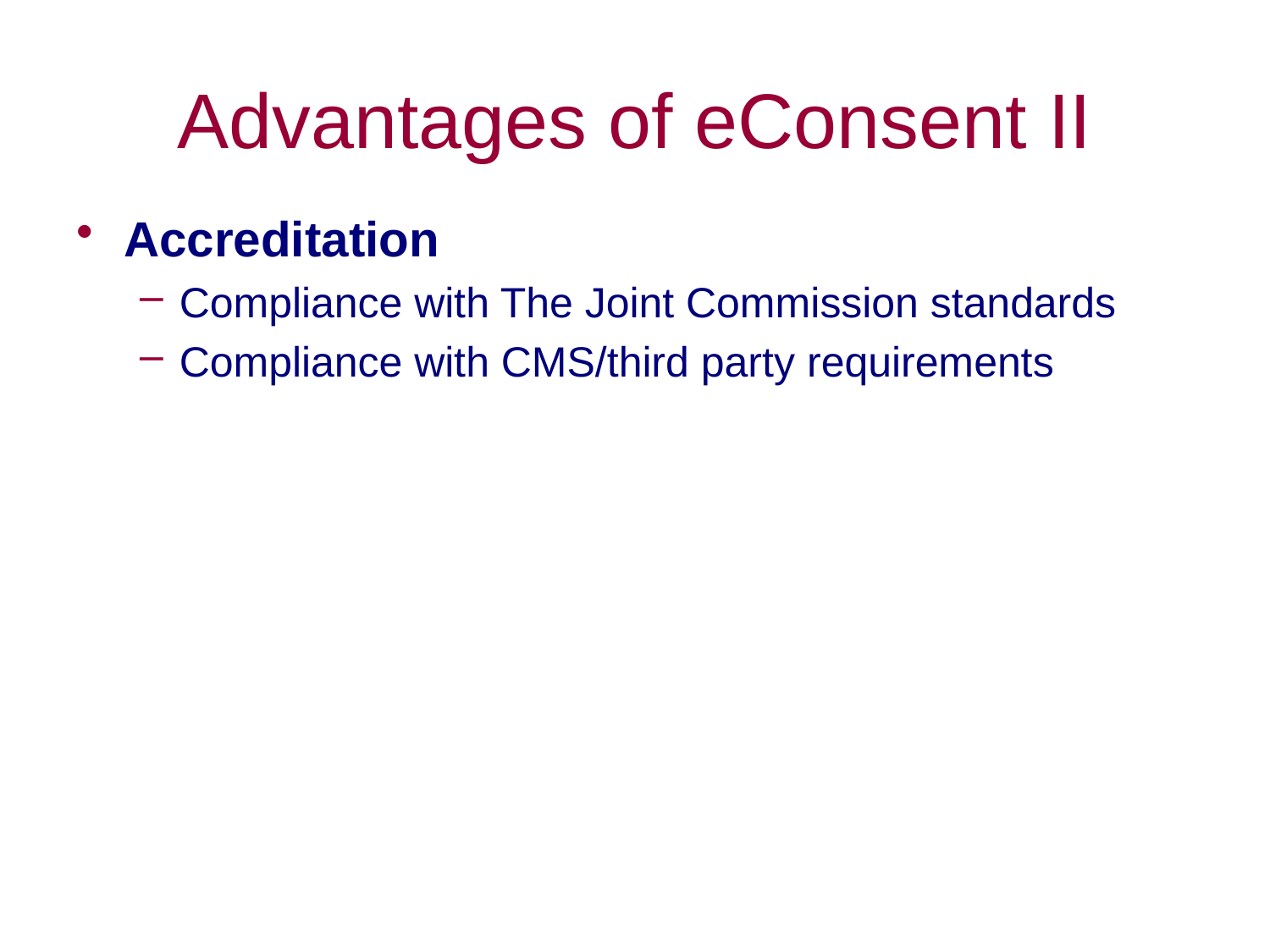

# Advantages of eConsent II
Accreditation
Compliance with The Joint Commission standards
Compliance with CMS/third party requirements

## Slide 75
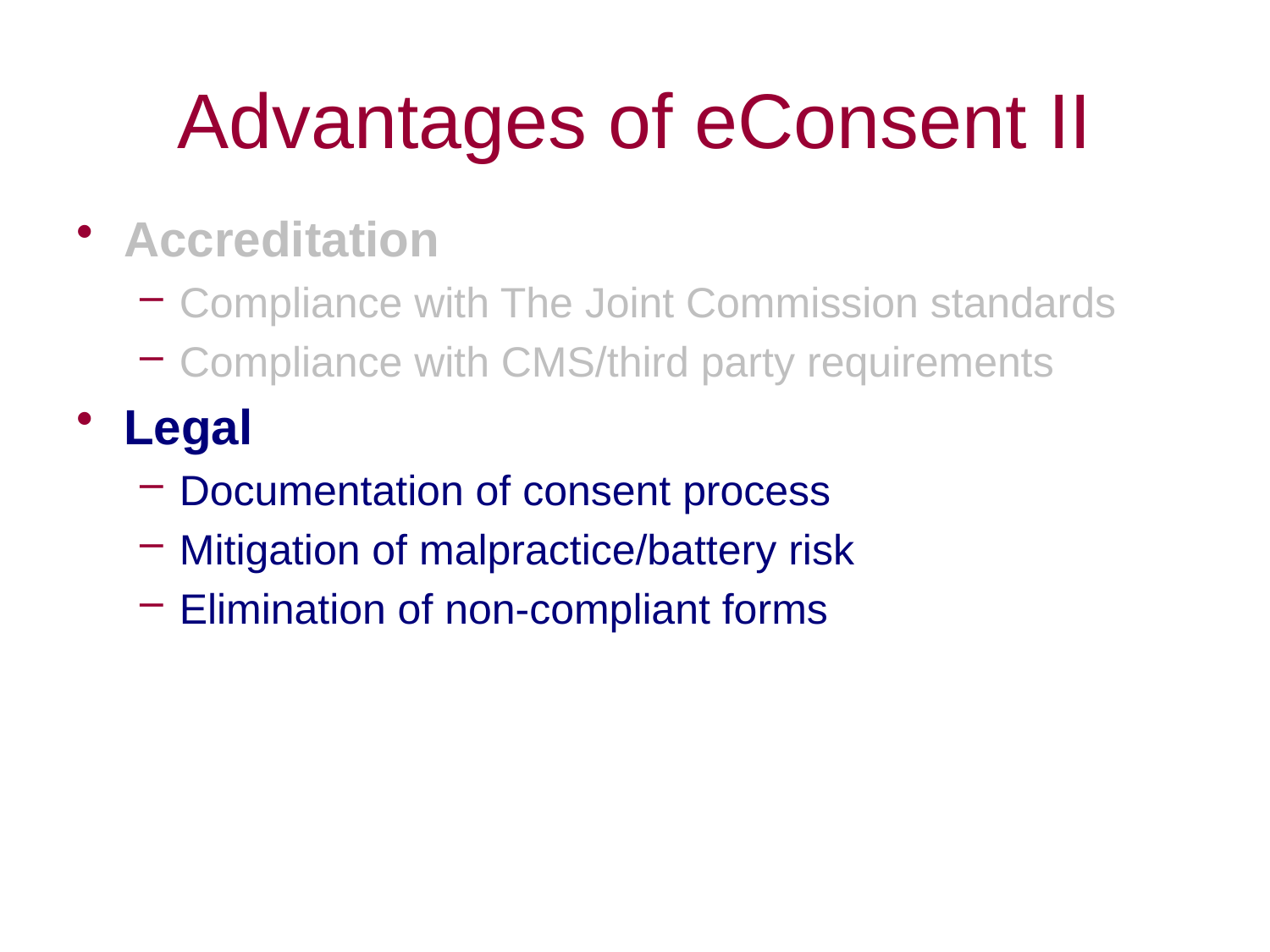

# Advantages of eConsent II
Accreditation
Compliance with The Joint Commission standards
Compliance with CMS/third party requirements
Legal
Documentation of consent process
Mitigation of malpractice/battery risk
Elimination of non-compliant forms

## Slide 76
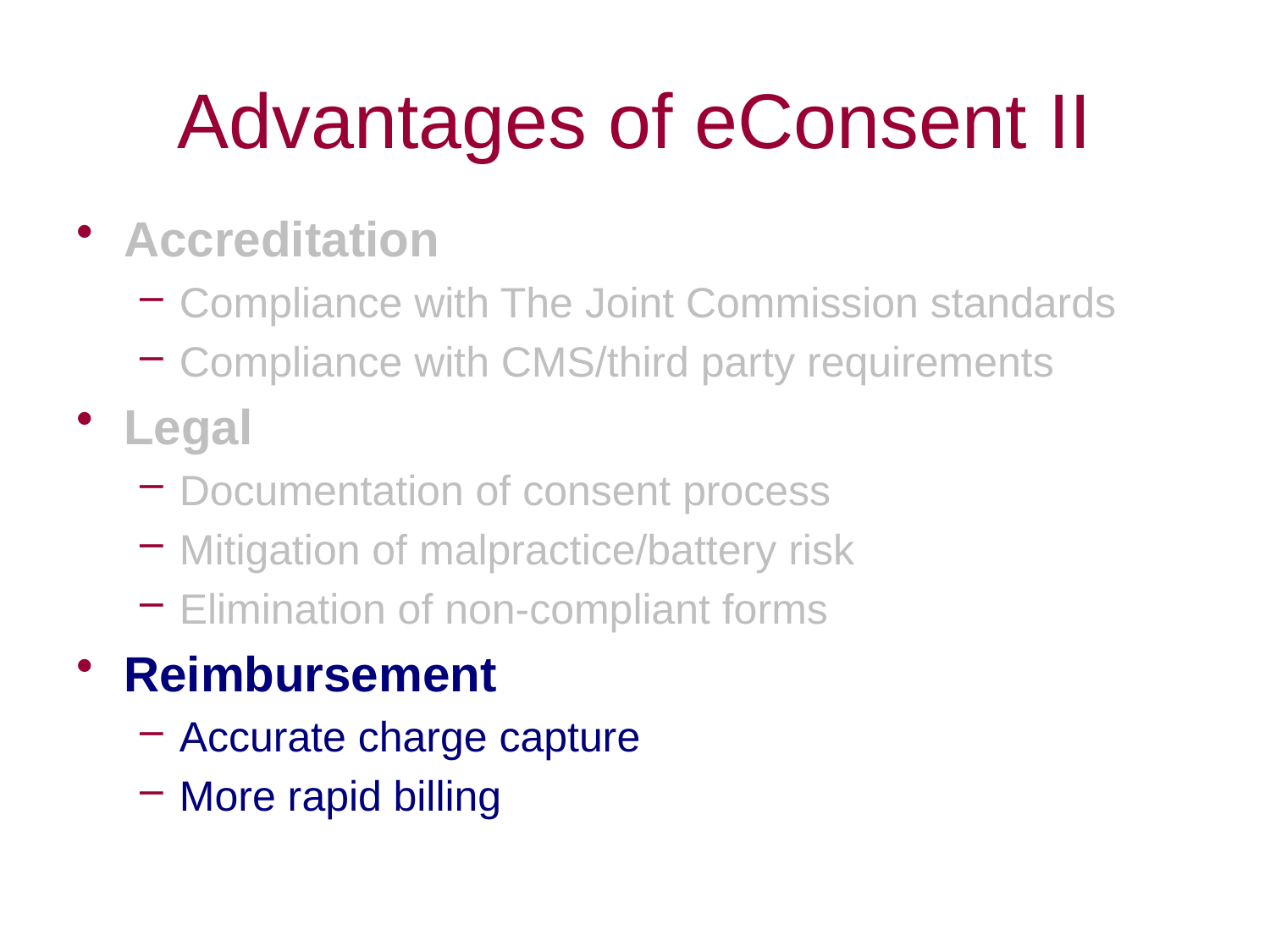

# Advantages of eConsent II
Accreditation
Compliance with The Joint Commission standards
Compliance with CMS/third party requirements
Legal
Documentation of consent process
Mitigation of malpractice/battery risk
Elimination of non-compliant forms
Reimbursement
Accurate charge capture
More rapid billing

## Slide 77
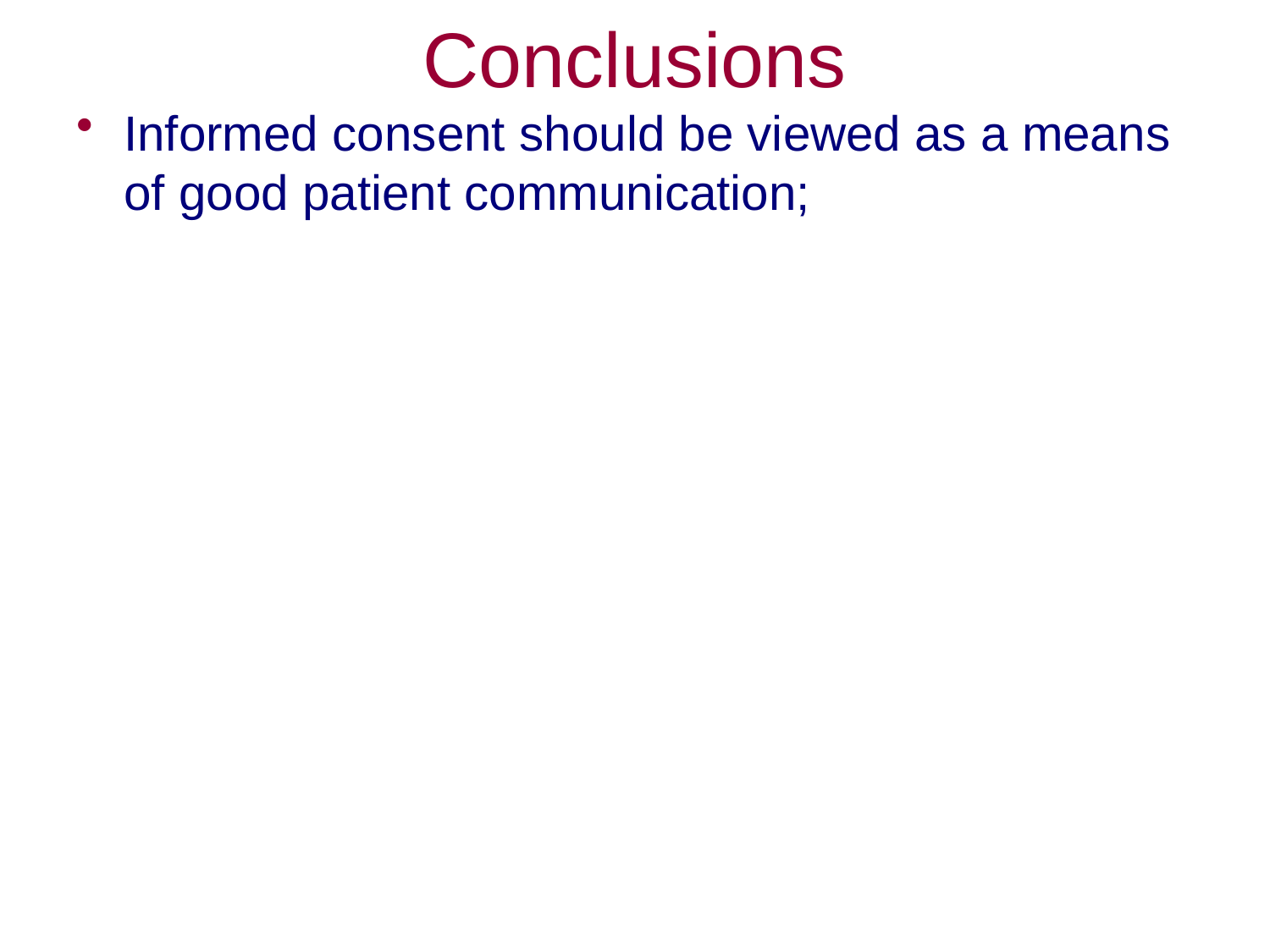

# Conclusions
Informed consent should be viewed as a means of good patient communication;

## Slide 78
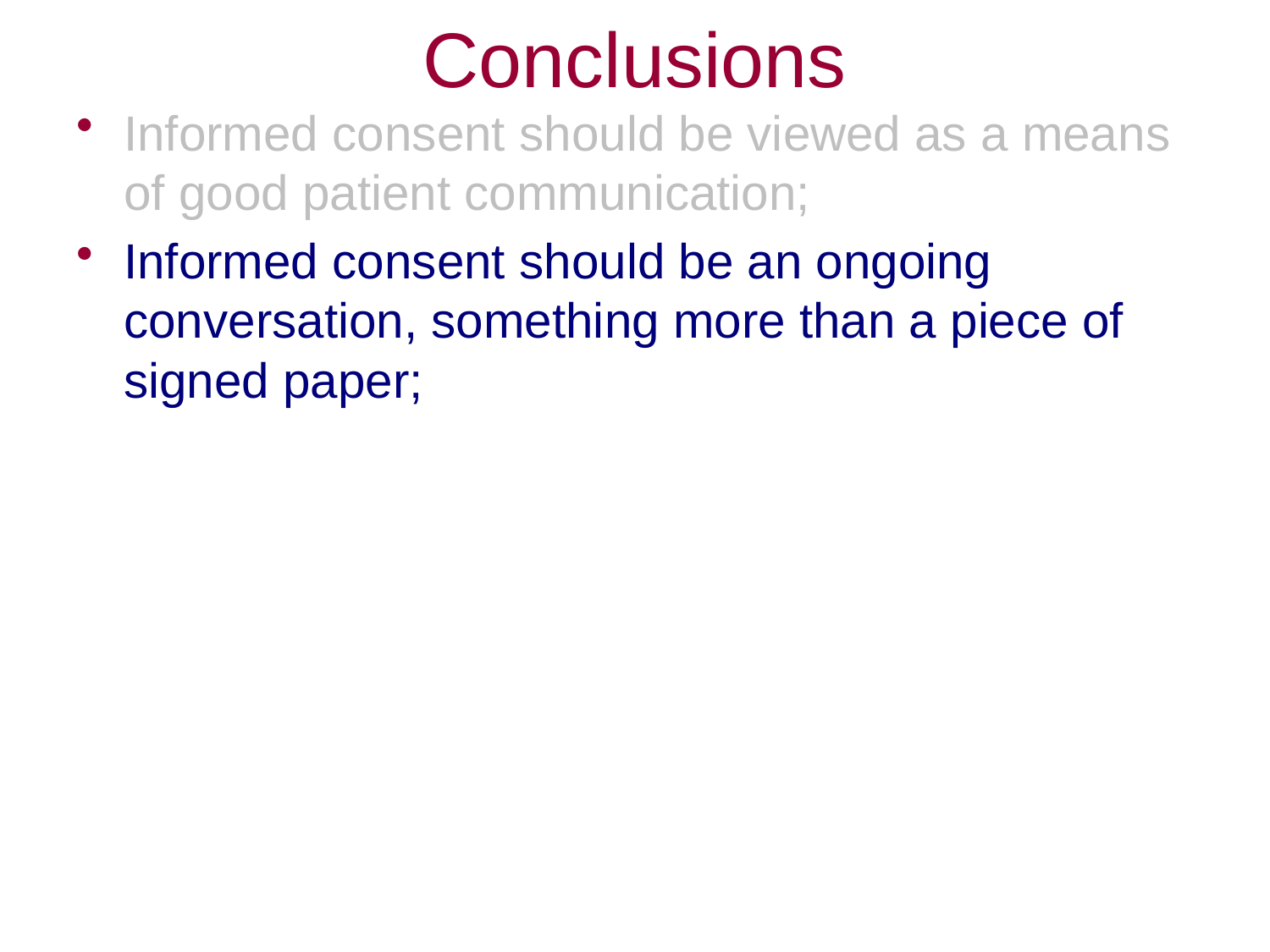

# Conclusions
Informed consent should be viewed as a means of good patient communication;
Informed consent should be an ongoing conversation, something more than a piece of signed paper;

## Slide 79
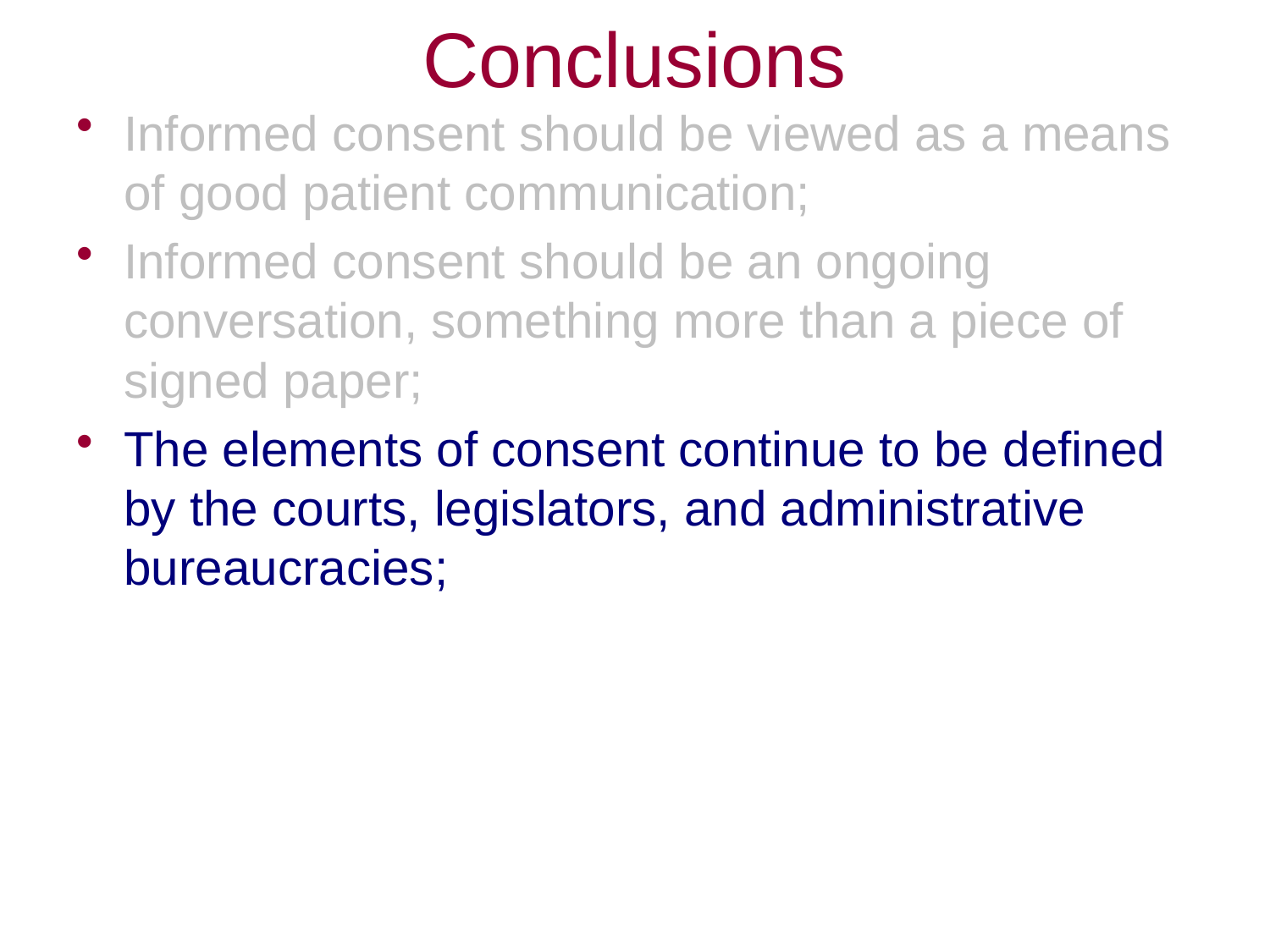

# Conclusions
Informed consent should be viewed as a means of good patient communication;
Informed consent should be an ongoing conversation, something more than a piece of signed paper;
The elements of consent continue to be defined by the courts, legislators, and administrative bureaucracies;

## Slide 80
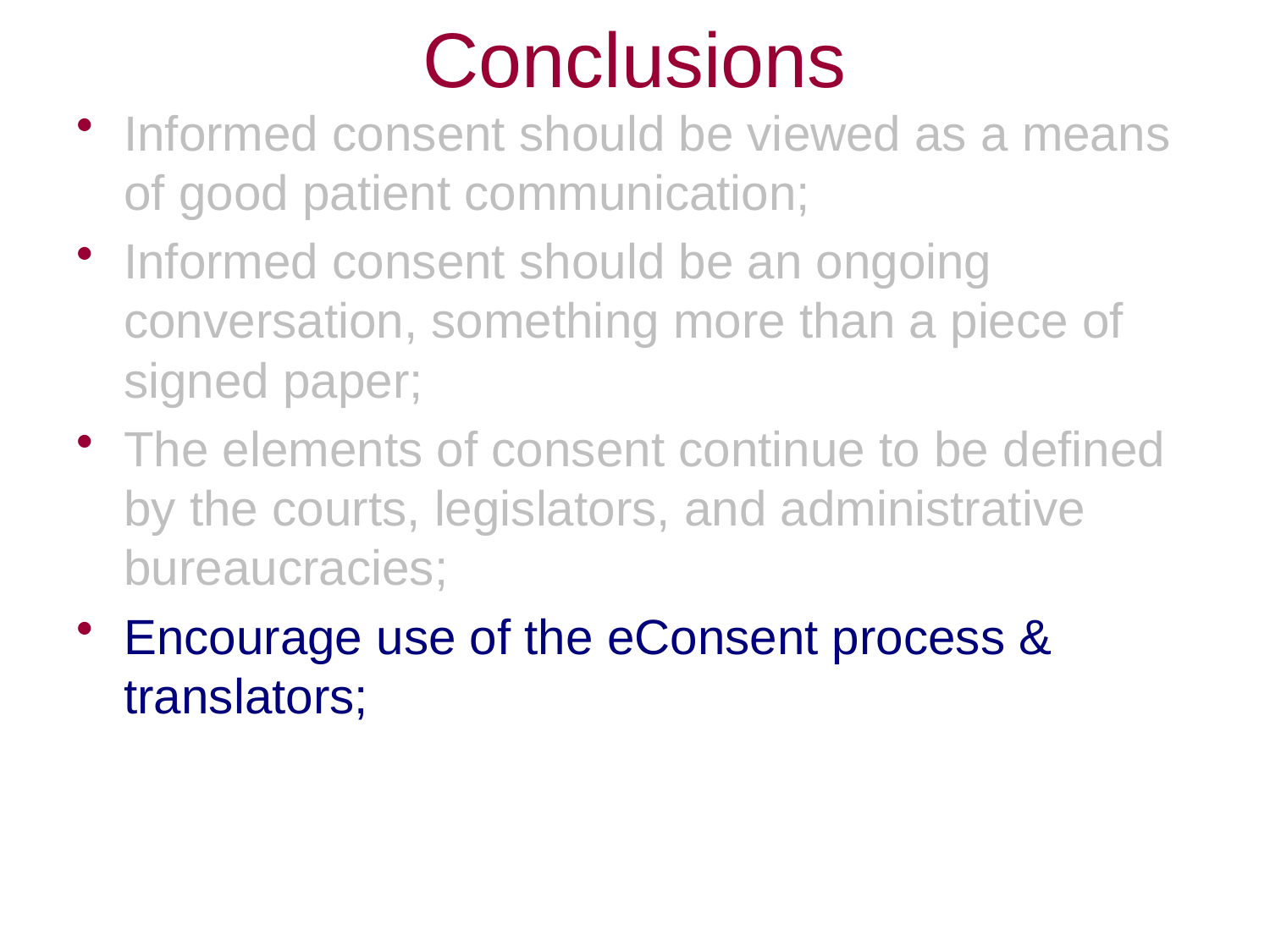

# Conclusions
Informed consent should be viewed as a means of good patient communication;
Informed consent should be an ongoing conversation, something more than a piece of signed paper;
The elements of consent continue to be defined by the courts, legislators, and administrative bureaucracies;
Encourage use of the eConsent process & translators;

## Slide 81
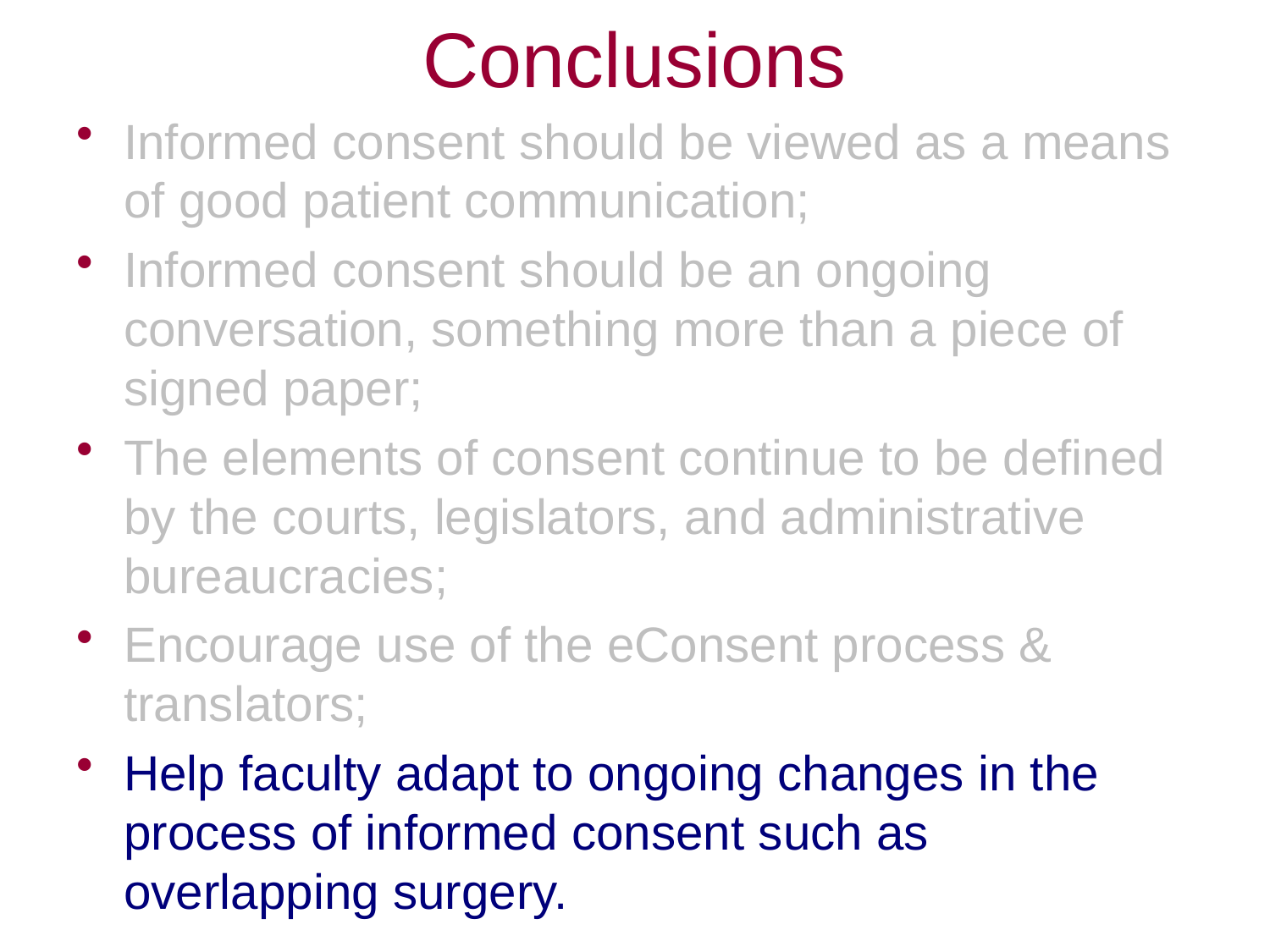

# Conclusions
Informed consent should be viewed as a means of good patient communication;
Informed consent should be an ongoing conversation, something more than a piece of signed paper;
The elements of consent continue to be defined by the courts, legislators, and administrative bureaucracies;
Encourage use of the eConsent process & translators;
Help faculty adapt to ongoing changes in the process of informed consent such as overlapping surgery.
