# Supplementary material for: Informed Consent for Academic Surgeons: A Curriculum-Based Update
Source: MedEdPORTAL. 2020 Oct 1;16:10985. doi: 10.15766/mep_2374-8265.10985 (PMC7528671; doi:10.15766/mep_2374-8265.10985)
Supplement: Supplementary file 1 — Informed Consent Update Slide Deck.pptxFacilitator Guide.docxInformed Consent Update Evaluation.docxKnowledge Posttest Questions.docx [file mep_2374-8265.10985-s001.zip › B. Facilitator Guide.docx]

Appendix B: Facilitator Guide

Development of Course Content

We based course content on informal discussion with faculty, administrators, and the Office of General Counsel. The lead author (SER) taught the course and also reviewed content with Penn’s Office of General Counsel for legal accuracy. The active surgical faculty of the Department of Surgery, University of Pennsylvania were targeted by our course. We selected the facilitators (faculty members who coordinated the rounds for the various divisions and talked it up among colleagues) to be surgeons in the discipline to which the course was directed (i.e. practicing surgeons). The only additional suggested preparation is for course instructors to meet with health system attorneys to ensure statutes, regulations, and case law are correct for the jurisdiction in which the course is taught.

Some prompting questions would include: What is the standard in the state? (e.g professional, reasonable patient, subjective); What clinical (i.e. non-research) procedures require signed informed consent documents? (e.g. surgery, chemotherapy, radiation therapy, blood transfusion); What are the leading informed consent cases in the jurisdiction? What regulations (e.g state board of medicine, department of health) are in effect? And who must sign for consent to be valid? (e.g surgeon, surrogate (e.g. resident, fellow, advanced practitioner) for surgeon, witness). We suggest presenting the course within the confines of usual time slots allocated to educational activities; the course takes about one hour to teach.

To maximally disseminate the material, we held four live sessions; surgery grand rounds, cardiac surgery, plastic surgery, and urology rounds, but depending on an organization’s educational activities are structured, one session for all surgeons may be adequate. arrange for CME credit where possible is an additional benefit for attendees. The usual assembly location can be used as for other educational conferences if audiovisual technology is available; a workstation or laptop computer hooked up to room-sized monitors or projection devices. The satisfaction surveys should be distributed as attendees enter, and collected as they leave. For a post-test comprehension assessment if provided, either paper forms can be distributed and collected or an institutionally owned or online audience response tool can be used if available. Audience responses are beneficial as aggregate data can be immediately fed back and where necessary, additional education imparted.

Slide Deck Notes

I suggest you work with someone skilled in AV to change out the slide master to your own institution. I prefer to use duplicate slides rather than animation, and ‘grey out’ already presented material. There is narration on the slides that is currently turned off. We did this ourselves using the PowerPoint capability and it is not the same quality you could use with professional editors (but much cheaper and faster)! For purposes of your own course, feel free to narrate in your own voice if you plan to post online. The slide deck contains a ‘script’ for each of the slides and can be read. Important references are also provided here should more detail be desired.

Slides 1-12 - Introduction, Outline, and Ethical theory (may want to mention that only standard types of surgical consent will be covered. Many special cases of consent (minors, emergency care, lack of capacity) could be considered in future courses.

Slides 13-23 - Legal evolution. This material starts with the Supreme Court’s justification for compelling physicians to provide informed consent; and why such compulsion does not violate the First Amendment. The cases presented are the first nation-wide to articulate a specific refinement of the doctrine of informed consent. All states have incorporated these refinements into state case law. Your Office of General Counsel can provide the state-specific cases on point as well as any state-specific statutes and regulations.

Slides 24-33 - Current regulations of the Centers for Medicare and Medicaid Services (CMS). I update slide 33 as needed and the CMS website is provided in the slides notes for #33. Accessing the data is accomplished by pointing your browser to the CMS website [Quality, Safety & Oversight – Certification & Compliance](https://www.cms.gov/Medicare/Provider-Enrollment-and-Certification/CertificationandComplianc)**.** https://www.cms.gov/Medicare/Provider-Enrollment-and-Certification/CertificationandComplianc/Hospitals (Accessed March 13, 2020) and then the ‘Full-Text-Statements-of-Deficiencies-Hospital-Surveys’ which will lead to the data file. The two specific citations on slides 34 & 35 come from the data file and involve, respectively, providers other than the operative surgeon who do significant parts of an operation, and inadequate attention to literacy and translation needs.

Slides 34-38 - Use a summary of the American College of Surgeons Statements of Principles (web address provided on slide notes) as an example of the professional obligations promulgated by the various professional societies.

Slides 39-51 – Provided information about a significant change in consent requirements. These slides detail responses by CMS and the American College of Surgeons regarding the issue of overlapping surgery.

Slides 52-55 – To highlight the ongoing evolution of the informed consent process, four issues are discussed. Some or all will likely impact the consent process and highlight the fact that surgeons will need to remain adaptable in the approach to providing consent to patients. First is a symposium generated by the American Bar Association Committee on Bioethics.

Slides 56 & 57 - These slides present a study documenting that, despite the guidance provided by CMS and the professional societies, many errors remain in the consent process leading to inadequately documented informed consent and significant health care costs. The data are a lead-in to the next topic.

Slides 58-61 – Discuss a CMS ‘Quality of Informed Consent Documents measure’ which will likely require health care organizations to provide data on adequacy of consent.

Slides 62-68 – Discuss issues of social justice. Despite much consideration of patient autonomy and physician beneficence, very little consideration has been given to this third ethical imperative. Social justice issues will likely assume greater importance in the future as evidenced by the Joint Commission.

Slides 69-76 – Informed consent is evolving to an online process. The many benefits are articulated here.

Slides 77-81 – These slides highlight key take-home points.
