# Supplementary material for: Informed Consent for Academic Surgeons: A Curriculum-Based Update
Source: MedEdPORTAL. 2020 Oct 1;16:10985. doi: 10.15766/mep_2374-8265.10985 (PMC7528671; doi:10.15766/mep_2374-8265.10985)
Supplement: Supplementary file 1 — Informed Consent Update Slide Deck.pptxFacilitator Guide.docxInformed Consent Update Evaluation.docxKnowledge Posttest Questions.docx [file mep_2374-8265.10985-s001.zip › C. Informed Consent Update Evaluation.docx]

Informed Consent Update Evaluation

Please rate today’s Informed Consent Update in the following categories using the provided scale (choose one):

❑ Faculty ❑ Resident/Fellow ❑ Advanced Practitioner

The goals of today’s session were clearly communicated.

Strongly Disagree Strongly Agree

❑1 ❑2 ❑3 ❑4 ❑5

Review of ethical principles increased my awareness of the foundations of Informed Consent.

Strongly Disagree Strongly Agree

❑1 ❑2 ❑3 ❑4 ❑5

The materials presented suggested opportunities for improving physician-patient communication through Informed Consent.

Strongly Disagree Strongly Agree

❑1 ❑2 ❑3 ❑4 ❑5

The discussion of PA-specific law helped me better understand my obligations in the Informed Consent process.

Strongly Disagree Strongly Agree

❑1 ❑2 ❑3 ❑4 ❑5

I better understand the various regulation governing the process of Informed Consent.

Strongly Disagree Strongly Agree

❑1 ❑2 ❑3 ❑4 ❑5

The materials regarding Informed Consent obligations in overlapping surgery were informative.

Strongly Disagree Strongly Agree

❑1 ❑2 ❑3 ❑4 ❑5

This Update will help me in future Informed Consent interactions with patients and families.

Strongly Disagree Strongly Agree

❑1 ❑2 ❑3 ❑4 ❑5

What was the most valuable part of this session?

What improvements would you suggest for future sessions?
