# Supplementary material for: Informed Consent for Academic Surgeons: A Curriculum-Based Update
Source: MedEdPORTAL. 2020 Oct 1;16:10985. doi: 10.15766/mep_2374-8265.10985 (PMC7528671; doi:10.15766/mep_2374-8265.10985)
Supplement: Supplementary file 1 — Informed Consent Update Slide Deck.pptxFacilitator Guide.docxInformed Consent Update Evaluation.docxKnowledge Posttest Questions.docx [file mep_2374-8265.10985-s001.zip › D. Knowledge Posttest Questions.docx]

Appendix D: Knowledge Posttest Questions^[[1]](#footnote-1)^

| Question Asked |
| --- |
| 1. In [Insert State] , inadequate informed consent is legally negligence (T/F) |
| 2. In [Insert State] a physician has an affirmative duty to disclose his/her professional credentials, training and experience performing the particular procedure for which consent is being secured (T/F) |
| 3. In [Insert State], the standard for judging the adequacy of the informed consent is based on the consensus in the medical community on what information about the procedure, the risks and the alternatives is required to make an informed decision (i.e. Professional Standard) (T/F) |
| 4. In [Insert State], only the performing surgeon is allowed to provide information regarding informed consent (T/F) |
| 5. In [Insert State], to recover in an action for lack of informed consent, the patient must prove that receiving the information that was not provided would have been a substantial factor in his/her decision to undergo the procedure (T/F) |

1. The Office of General Counsel at your institution should be consulted to insure correct answers to these questions. [↑](#footnote-ref-1)
