# Supplementary material for: Informed Consent for Academic Surgeons: A Curriculum-Based Update
Source: MedEdPORTAL. 2020 Oct 1;16:10985. doi: 10.15766/mep_2374-8265.10985 (PMC7528671; doi:10.15766/mep_2374-8265.10985)
Supplement: Supplementary file 1 — Informed Consent Update Slide Deck.pptxFacilitator Guide.docxInformed Consent Update Evaluation.docxKnowledge Posttest Questions.docx [file mep_2374-8265.10985-s001.zip › _Educational_Summary_Report_10985.pdf]

MedEdPORTAL® The AAMC Journal of Teaching and Learning Resources

Original Publication Open Access

Informed Consent for Academic Surgeons: A Curriculum-Based Update

Steven E. Raper, MD, JD*, Johncy Joseph, RN, MBA

*Corresponding Author: steven.raper@pennmedicine.upenn.edu

Abstract

Introduction: The principles of consent are evolving but remain an important part of the surgeon-patient relationship. The goal of this course was a concise, contemporary review of the principles of informed consent that would be favorably received by academic surgeons. **Methods:** The curriculum consisted of ethicohistorical and legal principles, current requirements, and new consent developments. An anonymous, voluntary evaluation tool was used to assess strengths and opportunities for improvement. A short postcourse quiz was developed to assess understanding. **Results:** Eighty-five percent of the surgery department faculty participated. Evaluations were overwhelmingly positive, all elements having weighted averages of greater than 4.5 on a 5-point Likert scale (1 = *strongly disagree,* 5 = *strongly agree*). Furthermore, a majority of respondents for the posttest got the answers correct for all five questions asked on the postcourse quiz. **Discussion:** A proper understanding of informed consent remains critically important in the practice of surgery. This short course updating surgeons on informed consent quantitatively confirms the favorable reception of this approach in terms of attendance and satisfaction, as well as understanding of the material.

Keywords
Informed Consent, Faculty Education, Accreditation Council for Continuing Medical Education, Surgeon-Patient Communication, Law of Consent, Clinical/Procedural Skills Training

Educational Objectives

By the end of this activity, learners will be able to:

1. Synthesize new knowledge with previous knowledge to describe better the ethical and legal foundations underpinning contemporary informed consent, current regulatory requirements for informed consent, and recent and future developments in informed consent doctrine.
2. Develop competence by logistical planning and learning how to use the materials in a course on informed consent.
3. Develop performance skills using the materials provided to teach faculty about current concepts of informed consent.

Introduction

Informed consent of the patient is a constantly evolving ethical imperative, legal duty, and professionally required responsibility imposed on surgeons prior to the performance of an operation.[1-3] Informed consent is also a means for enhancing surgeon-patient communication.[4] Physicians with higher numbers of unsolicited patient complaints—often involving poor communication—are more likely to get sued; hence, good communication may be protective against lawsuits.[5] Informed consent and other forms of surgeon-patient communication are thus important risk-management tools.[6] Informed consent has also been positively correlated with patient satisfaction.[7] Surgical consent discussions help make patients aware of a cost-benefit deliberation, establish trust in their surgeons, and may cause them to reconsider decisions previously made.[8]

All surgeons are familiar with the need to get informed consent prior to performing surgery on patients, yet there is a question of whether the informed consent process is as robust as it should be. Patient comprehension has been estimated to be less than 50%, a possible reflection of less than completely informed consent.[9] Surveys assessing the adequacy of consent have found that substantial numbers of consents do not contain essential elements.[10] Furthermore, interview-based research has suggested that consent discussions as currently practiced function as mere ritual, with little autonomy for patients.[11]

There have also been some recent developments worth noting. The oft-cited ideal of shared decision-making has been called into question.[12] The United States Senate Finance

Citation:
Raper SE, Joseph J. Informed consent for academic surgeons: a curriculum-based update. *MedEdPORTAL.* 2020;16:10985.
https://doi.org/10.15766/mep_2374-8265.10985

Copyright © 2020 Raper and Joseph. This is an open-access publication distributed under the terms of the Creative Commons Attribution license.

Committee has determined that surgeons should be required to disclose whether patients' surgical procedures will overlap with those of other patients.[13] There is scrutiny by the Centers for Medicare and Medicaid Services (CMS) of the adequacy of consent.[14]

Although most surgeons know something of the importance of informed consent, currently, the process is riddled with problems. Operating rooms are expected to run like clockwork, but a Johns Hopkins study documented that consent forms were missing in 66% of operations, delaying 14% of all surgical procedures, disrupting timed antibiotics, making extra work for OR staff (house staff, nurses, administrative assistants), and estimated that lost or misplaced consents cost an average hospital $580,000 per year.[15]

For all the reasons mentioned above, now is an opportune time to refocus surgeon awareness on matters of informed consent. Our contribution is unique; there is little published literature on strategies for formal education in the principles or practice of consent.[16,17] To update an academic surgical faculty on the informed consent process, a short course was developed to illuminate the past, present, and future of informed consent. The goal of the course was to help satisfy surgeons' educational needs for knowledge, competence, and performance by providing a concise, contemporary analysis of the principles of informed consent that would be favorably received by academic surgeons.[18]

Methods

Target Population and Practical Implementation Advice

We based course content on informal discussion with faculty, administrators, and the University of Pennsylvania's Office of the General Counsel. The lead author (Steven E. Raper) taught the course and also reviewed content with the Office of the General Counsel for legal accuracy. The active surgical faculty of the University of Pennsylvania's Department of Surgery were targeted by our course. We worked with faculty members embedded in the various divisions to coordinate the rounds of surgeons in the discipline to which the course was directed (i.e., practicing faculty surgeons). The only additional suggested preparation would be for course instructors to meet with health system attorneys to ensure statutes, regulations, and case law are correct for the jurisdiction in which the course is taught. Some prompting questions would include the following:

- What is the standard (e.g., professional, reasonable patient, subjective) in the state?
- What clinical (i.e., nonresearch) procedures (e.g., surgery, chemotherapy, radiation therapy, blood transfusion) require signed informed consent documents?
- What are the leading informed consent cases in the jurisdiction?
- What regulations (e.g., state board of medicine, department of health) are in effect?
- Who (e.g., surgeon, surrogate for surgeon [e.g., resident, fellow, advanced practitioner], witness) must sign for consent to be valid?

Course Curriculum Content

Course content consisted of material relevant to standard informed consent in adults with capacity for elective surgical procedures. A sharp focus on this topic left room for future courses dealing with urgent/emergent procedures, capacity, surrogate decision-making, other types of consent (e.g., chemotherapy), and advance directives. There was no prerequisite knowledge needed by the faculty participants, although all were actively involved in obtaining informed consent for their patients' operations. Much of the course content in the PowerPoint slide deck (Appendix A) and the facilitator guide (Appendix B) conveying the importance of informed consent, highlighting deficiencies in the current process, and explaining the new regulations regarding overlapping surgeries would be relevant to all surgeons and could be incorporated in similar courses throughout the larger surgical community (e.g., orthopedic surgeons, gynecologists). As a guide, we devoted 10-15 minutes to the past (ethical and legal underpinnings); 20-30 minutes to current law, regulations, and professional obligations (CMS, the Joint Commission, the American College of Surgeons); and 15-20 minutes to looking towards the future. Five to 10 minutes were allotted at the end for any questions and answers.

There were three parts to the curriculum. First, the past, or ethical underpinnings and a legal perspective on the evolution of informed consent, including a brief overview of ethicohistorical approaches to consent and the shift from the ethical principle of physician beneficence to patient autonomy. Ethical principles were elucidated from ancient times to the present.[1] The development of legal requirements through landmark case law was also discussed; we took care to present the legal issues in terms surgical audiences could grasp. The reasonable person standard focusing on materiality of the risks, alternatives, and consequences a reasonable person would want to know was discussed because it is the prevailing standard in most jurisdictions.[1]

Copyright © 2020 Raper and Joseph. This is an open-access publication distributed under the terms of the Creative Commons Attribution license.

Next, we went over current federal regulations and professional requirements. Requirements set forth by the CMS were discussed, including the Hospital Conditions of Participation.[14] Negligence or medical malpractice, a special case of the tort of negligence, was described since it is the cause of action in most jurisdictions.[1] All hospitals accepting Medicare must follow CMS regulations for all patients. CMS has issued interpretive guidelines called the *State Operations Manual,* which is the document used when inspectors come into health care facilities to audit compliance.[14] All deficiencies are transparently reported on the CMS website.[14] Furthermore, the Senate Finance Committee has jurisdiction over the Medicare and Medicaid programs. In December 2015, committee staff became aware of a surgical practice—referred to by hospitals as *concurrent, overlapping,* or *simultaneous surgeries*—from a *Boston Globe* article.[19] The committee released a report in which the findings were characterized as a patient safety issue.[13] In response, the American College of Surgeons revised its Statements on Principles.[3] A significant change in the latest version of the Statements on Principles is a discussion of the differences between concurrent and overlapping surgery.[3]

The last part of the course discussed several initiatives that would likely enhance the informed consent process in the near future. These included a recently published symposium arising from Town Halls on Informed Consent held by the American Bar Association,[20] a Quality of Informed Consent Documents measure proposed by CMS,[21] issues of social justice, and procedure-specific electronic informed consent documents.

This publication includes the PowerPoint slides (Appendix A) containing the course material. Also, a facilitator guide (Appendix B) with detailed course curriculum information, relevant references, and thoughts on how to more precisely tailor the slide deck to the audience is provided.

Survey Development and Analysis

An anonymous voluntary paper-based survey was provided to attendees so they could share their experience regarding the course with the faculty facilitators. The satisfaction survey (Appendix C) contained a space to designate the professional level of the responder, questions regarding the course material, and two free-text write-in, or hot, comments soliciting (a) the most useful material and (b) opportunities for improvement. The questions were adapted from similar ones asked of faculty and residents, as previously published.[4] The survey instrument used a 5-point Likert scale (1 = *strongly disagree,* 5 = *strongly agree*) to measure participant experience during the informed consent update in order to allow for improvement of future courses.[4,5] We analyzed and tabulated the survey results. The weighted average of each question was calculated using the following formula[22]: $\bar{X} = \Sigma W_i \times X_i / \Sigma W_i$, where $\bar{X}$ = weighted average of the responses, W_i = weight of the answer choice, and X_i = response count for answer choice. In addition to the Likert-scale questions, two free-text items were included for those who wished to provide further feedback. The University of Pennsylvania's Institutional Review Board did not consider our satisfaction survey and posttest to constitute human subjects research (dated December 6, 2017).

Posttest Comprehension

To attempt to determine if the material presented led to comprehension, a five-question posttest was developed. Some questions were written by the lead author (Steven E. Raper, Questions 1 and 4, Appendix D) and some by an attorney from the university health system's Office of the General Counsel (Questions 2, 3, and 5, Appendix D). As such, there was variability in wording; some questions were terser than others. After all responses were recorded, the correct answers and percentage for correct responses were calculated.

Results

One hundred Department of Surgery faculty members were eligible to participate in the course. Seventy-five attended the live lectures, and 10 viewed the course online, for a total participation rate of 85%. Attendance was tracked by an anonymized count of those who signed into the CME website or took the online version as provided by the CME office, and only totals were provided. Participation was encouraged by the usual email announcements. Prior to presenting the course, an arbitrary goal was set as 80% total attendance and 50% in the live setting; both goals were exceeded.

The evaluation survey (Appendix C) was distributed at the lecture or as a follow-up for the online course. There was a checkbox to designate the type of health care provider. Of the 85 faculty who participated, 48 filled out the anonymous voluntary evaluation, for a return rate of 56%. Only faculty evaluations were subsequently used for analysis. The evaluations were distributed before and retrieved at the end of the lecture. Evaluations were overwhelmingly positive for the seven Likert-type questions asked; all had a weighted average of greater than 4.5 (Table 1). A significant number of faculty also took the time to write in free-text or hot comments that were categorized with a simple content analysis. For "What was the most valuable part of this session?" there were 29 (60%) responses (Table 2). For "What improvements would you suggest for future sessions?" there were 19 (40%) responses on a variety of topics, including dissemination to advanced practitioners and

Copyright © 2020 Raper and Joseph. This is an open-access publication distributed under the terms of the Creative Commons Attribution license.

Table 1. Course Evaluation for Satisfaction

Question[a]	Weighted Average
The goals of today's session were clearly communicated.	4.8
Review of ethical principles increased my awareness of the foundations of IC.	4.6
The materials presented opportunities for improving physician-patient communication through IC.	4.6
The discussion of [state]-specific law helped me better understand my obligations in the IC process.	4.6
I better understand the various federal regulations governing the process of IC.	4.6
The materials regarding IC obligations in overlapping surgery were informative.	4.7
This update will help me in future IC interactions with patient and families.	4.7

Abbreviation: IC, informed consent.
[a]Rated on a 5-point Likert scale (1 = *strongly disagree*, 5 = *strongly agree*).

improved logistics of the online content (Table 3). Regarding postcourse comprehension, a majority of respondents gave the correct answers for each of the five questions asked on the posttest (Table 4).

Discussion

General Summary

To address the constantly evolving subject of surgical informed consent, we developed a course that covered not only the historical antecedents but also current requirements and some predictions about future changes. Because it was presented in several venues, the course was well attended, with an 85% faculty turnout. Satisfaction as measured by a survey was high, with greater than 4.5 out of 5 on a Likert-type scale in each of seven domains.

Assessment of Faculty Satisfaction

The attention to detail in trying to minimize the impact of the informed consent course on clinically busy surgeons was a relative strength of our approach. Getting surgical faculty—burdened by the competing demands of clinical care, education, and research—to attend lectures on quality and patient safety concerns like informed consent presented significant challenges.

Table 2. Answers to the Question "What Was the Most Valuable Part of This Session?"

Hot Comments	No.
Information on overlapping surgery	6
Posttest discussion	6
Discussion of the law	6
General	3
State-specific	3
History and evolution of consent	3
Institution-specific material	2
Future directions	2
Overall course	2
Lecturer	2

Table 3. Answers to the Question "What Improvements Would You Suggest for Future Sessions?"

Hot Comments	No.
Nothing	9
More case presentations	3
More pointers on improving consent documentation	3
Less background detail	1
Mandatory material for advanced practitioners	1
More posttest discussion	1
Online logistics	1

A long-term tripartite strategy pursued by Penn Surgery included focusing attention on issues that matter to surgeons, working with faculty embedded in the divisions involved, and offering some form of carrot rather than stick.[4] For the informed consent course, content was developed from offline conversations with faculty, the Office of the General Counsel, and an appreciation of recent events, some making national news. The course was presented in four venues to provide opportunities for all surgical faculty to attend in person. Time constraints imposed by the operating room and patient care made it essential to slot the presentations into time allotted to existing educational efforts.

Another important goal was to identify what did and did not register with faculty for purposes of developing future quality and patient safety presentations. Each of the seven areas assessed—communication of goals, ethical principle review, improved patient-physician communication, Pennsylvania-specific law, federal regulations, overlapping surgery obligations, and interactions with patients—scored weighted averages of greater than 4.5 (Table 1). If translated to a 100-point scale, scores would be greater than 90%, suggesting high satisfaction with the materials presented.

Lessons Learned

We suggest presenting the course within the confines of usual time slots allocated to educational activities; the course takes about 1 hour to teach. To maximally disseminate the material, we held four live sessions—surgery grand rounds, cardiac surgery, plastic surgery, and urology rounds—but depending on how an organization's educational activities are structured, one session for all surgeons may be adequate. Arranging for CME credit where possible is an additional benefit for attendees. The

Table 4. Posttest Questions and Correct Answers

Question	Correct Answer	No. Correct/Total No. (%)
1	True	99/103 (96)
2	False	81/112 (72)
3	False	62/107 (58)
4	True	96/98 (98)
5	True	77/97 (79)

Copyright © 2020 Raper and Joseph. This is an open-access publication distributed under the terms of the Creative Commons Attribution license.

usual assembly location can be used as for other educational conferences if audiovisual technology (a workstation or laptop computer hooked up to room-sized monitors or projection devices) is available. The satisfaction surveys should be distributed as attendees enter and be collected as they leave. If a posttest comprehension assessment is provided, either paper forms can be distributed and collected or an institutionally owned or online audience response tool can be used if available. Audience responses are beneficial as aggregate data can be immediately fed back and, where necessary, additional education imparted.

Limitations of the Study

Given the complex nature of the material presented, the posttest was developed to assess comprehension at a basic level. However, attributing correct answers on the posttest to the efficacy of the curriculum is difficult without a pretest baseline, which is a weakness of our study design. A pretest could additionally be used to telegraph important principles to be discussed during the course. A decision was made to use true/false questions rather than multiple-choice questions. The material tested was basic knowing, the lowest level in Bloom's cognitive domain.[23]

A cursory examination of the questions suggests that some were presented in a terser, more straightforward way; these questions (Questions 1 and 4, Appendix D) had the highest rate of correct answers. Question 3 had the lowest percentage of correct responses (Appendix D). The goal was to determine a baseline level of understanding, not whether the success rates had improved. How the wording of the material in a question might impact its ability to assess basic principles, as opposed to winnowing out those who do not pay close attention, has not been addressed. The small sample size represented here precludes any sweeping conclusions but is a matter worthy of more study.

There were at least three additional inadequacies in the posttest design. First, participants were not required to answer all questions. Next, the audience response system was not capable of being set up to allow identification by participant role (i.e., faculty, house staff, fellow, advanced practitioner). It would be disingenuous to attribute the high posttest scores to faculty alone. However, although house staff, fellows, and advanced practitioners were allowed to attend, no attempt to encourage them was made for this course, which was focused on faculty. Not all participants answered all questions. Fellows, house staff, and advanced practitioners were not excluded from the presentations, and some presumably also answered the postcourse quiz; hence, the total number of responses was greater than the number of participating faculty. There was no way to sort the answers by level of education or role. Lastly, true/false questions were offered rather than multiple-choice ones, limiting the ability of the questions to get past the lowest level of Bloom's cognitive domain, remembering. Future courses will address these limitations in the current study. There is a great deal of literature on writing effective multiple-choice questions and on how to frame them in terms of the levels of Bloom's revised taxonomy in the cognitive domain (in ascending order, remembering, understanding, applying, analyzing, evaluating, and creating)—especially the higher levels, those that correspond to competence and performance.[23,24] For truly informed consent, the affective domain is also relevant since effective communication between providers and patients/families is important.[25,26]

Comparison to Other Surgery Faculty Development Initiatives

There is a small literature with which to compare this curriculum. Surgical faculty have developed an extended quality improvement curriculum for surgical residents that includes formal didactics and structured practical experience.[27] One-hour frame-of-reference training sessions are likely sufficient to train surgical faculty to reliably use a simple evaluation instrument for the assessment of intraoperative performance.[28] Faculty development geared toward sessions on communication skills, patient education, informed consent, shared decision-making, and delivering bad news has been shown to be beneficial to educational activities in a clinical department.[29] Programs like the University of Michigan Surgery Innovation and Entrepreneurship Development Program can educate surgeons and other academicians on innovation, entrepreneurship, and commercialization.[30] A half-day course aimed at enhancing intraoperative instruction can contribute to resident-perceived improvement in structured teaching behavior among participating faculty.[31] An electronic survey of Yale surgery faculty and residents has evaluated their global-based experiences, measured interest in the development of international electives, and enumerated barriers to the development of global opportunities.[32] East Carolina University has developed the Teachers of Quality Academy with avowed dual goals of preparing faculty to lead clinical transformation while becoming proficient in designing curricula to prepare students in health systems science competencies.[33]

Conclusions

Surgical faculty were instructed in current principles of informed consent in a course that was well received. Consent was framed

Copyright © 2020 Raper and Joseph. This is an open-access publication distributed under the terms of the Creative Commons Attribution license.

as a means of good patient communication; good communicators were less likely to get sued and may have better outcomes. Informed consent should be an ongoing conversation, something more than a mere piece of signed paper. The elements of consent continue to be defined by the courts, legislators, and administrative bureaucracies and continue to evolve. Depending on the state in which academic surgical faculty practice, the local and state-specific laws will need to be particularized. More scrutiny will be placed on documentation that the patient actually understands the planned operation. If a surgeon performs overlapping surgery, that surgeon must consider how to discuss the role of qualified practitioners operating in his or her absence. CMS and the states will scrutinize this process in future inspections.

Future Directions

Enhancing surgeon-patient communication has been a main focus of our work. There are a number of specific physician-patient communication encounters, each requiring a specific skill set, and the topics of capacity, competence, implied consent, surrogate decision-making, and advance directives are all appropriate for future courses.[1,34-36] Recently, we taught a course for faculty on principles of outpatient communication, including the push to transparency and public reporting of patient satisfaction data. We recently published our experience with advanced practitioners in a course on principles of inpatient communication.[37] With regard to informed consent, we are developing a research agenda seeking to empirically examine and address factors that shape the informed consent process. As discussed above, there are numerous dynamics that shape consent to surgery—complex dynamics with ethical, legal, cultural, organizational, and even political dimensions. Outcomes research on informed consent is hampered by lack of consensus on exactly what a good decision is and how it can be measured. Assessment of knowledge transfer is but one outcome metric; others may better reflect decision-making quality.

Appendices

A. Informed Consent Update Slide Deck.pptx

B. Facilitator Guide.docx

C. Informed Consent Update Evaluation.docx

D. Knowledge Posttest Questions.docx

All appendices are peer reviewed as integral parts of the Original Publication.

Steven E. Raper, MD, JD: Associate Professor and Vice-chair for Quality and Risk Management, Department of Surgery, Perelman School of Medicine at the University of Pennsylvania; ORCID: https://orcid.org/0000-0001-9191-4681

Johncy Joseph, RN, MBA: Quality Manager, Department of Surgery, Penn Medicine

Disclosures
Johncy Joseph is founder and CEO of Avenir Solutions, a professional development company.

Funding/Support
None to report.

Prior Presentations
Raper SE, Joseph J. An informed consent update for academic surgeons. Presented at: 13th Annual Academic Surgical Congress; January 31, 2018; Jacksonville, FL.

Ethical Approval
Reported as not applicable.

References

1. Faden RR, Beauchamp TL. *A History and Theory of Informed Consent*. Oxford University Press; 1986:3-43.
2. *Salgo v Leland Stanford Jr. University Board of Trustees*, 317 P2d 170 (Cal Ct App 1957).
3. Statements on Principles. American College of Surgeons. *Updated April* 12, 2016. Accessed November 1, 2018. http://www.facs.org/about-acs/statements/stonprin
4. Raper SE, Gupta M, Okusanya O, Morris JB. Improving communication skills: a course for academic medical center surgery residents and faculty. *J Surg Educ*. 2015;72(6): e202-e211. https://doi.org/10.1016/j.jsurg.2015.06.008
5. Raper SE, Joseph J, Seymour WG, Sullivan PG. Tipping the scales: educating surgeons about medical malpractice. *J Surg Res*. 2016;206(1):206-213. https://doi.org/10.1016/j.jss.2016.08.002
6. Raper SE, Rose D, Nepps ME, Drebin JA. Taking the initiative: risk-reduction strategies and decreased malpractice costs. *J Am Coll Surg*. 2017;225(5):612-621. https://doi.org/10.1016/j.jamcollsurg.2017.07.1070
7. Hallock JL, Rios R, Handa VL. Patient satisfaction and informed consent for surgery. *Am J Obstet Gynecol*. 2017;217(2): 181.e1-181.e7. https://doi.org/10.1016/j.ajog.2017.03.020
8. Hall DE, Morrison P, Nikolajski C, Fine M, Arnold R, Zickmund SL. Informed consent for inguinal herniorrhaphy and cholecystectomy: describing how patients make decisions to have surgery. *Am J Surg*. 2012;204(5):619-625. https://doi.org/10.1016/j.amjsurg.2012.07.020

Copyright © 2020 Raper and Joseph. This is an open-access publication distributed under the terms of the Creative Commons Attribution license.

9. Fink AS, Prochazka AV, Henderson WG, et al. Predictors of comprehension during surgical informed consent. *J Am Coll Surg*. 2010;210(6):919-926. https://doi.org/10.1016/j.jamcollsurg.2010.02.049
10. Ankuda CK, Block SD, Cooper Z, et. al. Measuring critical deficits in shared decision making before elective surgery. *Patient Educ Couns*. 2014;94(3):328-333. https://doi.org/10.1016/j.pec.2013.11.013
11. Dixon-Woods M, Williams SJ, Jackson CJ, Akkad A, Kenyon S, Habiba M. Why do women consent to surgery, even when they do not want to? An interactionist and Bourdieusian analysis. *Soc Sci Med*. 2006;62(11):2724-2753. https://doi.org/10.1016/j.socscimed.2005.11.006
12. Clapp JT, Arriaga AF, Murthy S, et al. Surgical consultation as social process: implications for shared decision making. *Ann Surg*. 2019;269(3):446-452. https://doi.org/10.1097/sla.0000000000002610
13. *Concurrent and Overlapping Surgeries: Additional Measures Warranted*. Senate Finance Committee; 2016. Accessed November 1, 2018. http://www.finance.senate.gov/imo/media/doc/Concurrent%20Surgeries%20Report%20Final.pdf
14. Quality, safety & oversight—certification & compliance: hospitals. Centers for Medicare & Medicaid Services. Accessed March 13, 2020. https://www.cms.gov/Medicare/Provider-Enrollment-and-Certification/CertificationandComplianc/Hospitals
15. Garonzik-Wang JM, Brat G, Salazar JH, et al. Missing consent forms in the preoperative area: a single-center assessment of the scope of the problem and its downstream effects. *JAMA Surg*. 2013;148(9):886-889. https://doi.org/10.1001/jamasurg.2013.354
16. Childers R, Lipsett PA, Pawlik TM. Informed consent and the surgeon. *J Am Coll Surg*. 2009;208(4):627-634. https://doi.org/10.1016/j.jamcollsurg.2008.12.012
17. Kinnersley P, Phillips K, Savage K, et al. Interventions to promote informed consent for patients undergoing surgical and other invasive healthcare procedures. *Cochrane Database Syst Rev*. 2013;(7):CD009445. https://doi.org/10.1002/14651858.cd009445.pub2
18. Singer S. Educational terms: knowledge, competence, performance, and patient outcomes. Accreditation Council for Continuing Medical Education. July 5, 2016. Accessed April 24, 2020. https://www.accme.org/resources/video-resources/accreditation-requirements/educational-terms-knowledge-competence-performance-and-patient-outcomes
19. Abelson J, Saltzman J, Kowalczyk L. Clash in the name of care. *Boston Globe*. October 25, 2015. https://apps.bostonglobe.com/spotlight/clash-in-the-name-of-care/story/
20. Koch VG, Elster NR. Under attack: reconceptualizing informed consent. *J Law Med Ethics*. 2017;45(1):6-9. https://doi.org/10.1177/1073110517703095
21. Potential inclusion of the Quality of Informed Consent Documents for Hospital-Performed, Elective Procedures Measure. *Fed Regist*. 2017;82(155):38362-38368.
22. Gelman A. Struggles with survey weighting and regression modeling. *Stat Sci*. 2007;22(2):153-164. https://doi.org/10.1214/088342306000000691
23. Forehand M. Bloom's taxonomy. Vanderbilt University. December 7, 2011. Accessed March 12, 2020. https://cft.vanderbilt.edu/wp-content/uploads/sites/59/BloomsTaxonomy-mary-forehand.pdf
24. Anderson LW, Krathwohl DR, eds. *A Taxonomy for Learning, Teaching, and Assessing: A Revision of Bloom's Taxonomy of Educational Objectives*. Longman; 2001.
25. Krathwohl DR, Bloom BS, Masia BB. *Taxonomy of Educational Objectives: The Classification of Educational Goals—Handbook II: Affective Domain*. David McKay Co; 1973.
26. Bloom's revised taxonomy: cognitive, affective, and psychomotor. Arkansas State University. Accessed March 12, 2020. https://www.astate.edu/dotAsset/7a3b152c-b73a-45d6-b8a3-7ecf7f786f6a.pdf
27. Sellers MM, Hanson K, Schuller M, et al. Development and participant assessment of a practical quality improvement educational initiative for surgical residents. *J Am Coll Surg*. 2013;216(6):1207-1213.e1. https://doi.org/10.1016/j.jamcollsurg.2013.01.065
28. George BC, Teitelbaum EN, DaRosa DA, et al. Duration of faculty training needed to ensure reliable OR performance ratings. *J Surg Educ*. 2013;70(6):703-708. https://doi.org/10.1016/j.jsurg.2013.06.015
29. Kalet AL, Janicik R, Schwartz M, Roses D, Hopkins MA, Riles T. Teaching communication skills on the surgery clerkship. *Med Educ Online*. 2005;10(1):4382. https://doi.org/10.3402/meo.v10i.4382
30. Servoss J, Chang C, Olson D, Ward KR, Mulholland MW, Cohen MS. The Surgery Innovation and Entrepreneurship Development Program (SIEDP): an experiential learning program for surgery faculty to ideate and implement innovations in health care. *J Surg Educ*. 2018;75(4):935-941. https://doi.org/10.1016/j.jsurg.2017.09.017
31. Gardner AK, Timberlake MD, Dunkin BJ. Faculty development for the operating room: an examination of the effectiveness of an intraoperative teaching course for surgeons. *Ann Surg*. 2019; 269(1):184-190. https://doi.org/10.1097/sla.0000000000002468
32. Cheung M, Healy JM, Hall MR, Ozgediz D. Assessing interest and barriers for resident and faculty involvement in global surgery. *J Surg Educ*. 2018;75(1):49-57. https://doi.org/10.1016/j.jsurg.2017.06.031
33. Baxley EG, Lawson L, Garrison HG, et al. The Teachers of Quality Academy: A learning community approach to preparing faculty to

Copyright © 2020 Raper and Joseph. This is an open-access publication distributed under the terms of the Creative Commons Attribution license.

teach health systems science. *Acad Med*. 2016;91(12): 1655-1660. https://doi.org/10.1097/acm.0000000000001262

34. Berg JW, Appelbaum PS, Lidz CW, Parker LS. *Informed Consent: Legal Theory and Clinical Practice*. Oxford University Press; 2001.

35. Zimmermann CJ, Jhagroo RA, Wakeen M, et al. Opportunities to improve shared decision making in dialysis decisions for older adults with life-limiting kidney disease: a pilot study. *J Palliat Med*. 2020;23(5):627-634. https://doi.org/10.1089/jpm.2019.0340

36. Sturgess J, Clapp JT, Fleisher LA. Shared decision-making in peri-operative medicine: a narrative review. *Anaesthesia*. 2019;74(S1):13-19. https://doi.org/10.1111/anae.14504

37. Joseph J, Sicoutris C, Raper SE. Communication skills training for surgical inpatient advanced practice providers in an academic health-care system. *J Patient Exp*. 2020;7(1):42-48. https://doi.org/10.1177/2374373518809011

Received: March 18, 2020
Accepted: April 20, 2020
Published: October 1, 2020

Copyright © 2020 Raper and Joseph. This is an open-access publication distributed under the terms of the Creative Commons Attribution license.
